# An Argonaute protein traffics from nematode to mouse and is a vaccine against parasitic nematodes

**DOI:** 10.1038/s44319-025-00620-4

**Published:** 2025-12-09

**Authors:** Kyriaki Neophytou, Isaac Martínez-Ugalde, Thomas M Fenton, Elaine Robertson, Lewis J Strachan, Vignesh Jayaraman, Yvonne Harcus, Chanel M Naar, David Wright, Daniel R G Price, Ruby White, Michael J Evans, José Roberto Bermúdez-Barrientos, Hanchen Li, Rick M Maizels, Raffi V Aroian, Alasdair J Nisbet, Cei Abreu-Goodger, Amy H Buck

**Affiliations:** 1 Institute of Immunology and Infection Research, School of Biological Sciences, The University of Edinburgh, Edinburgh, EH9 3FL UK; 2 Institute of Ecology and Evolution, School of Biological Sciences, The University of Edinburgh, Edinburgh, EH9 3FL UK; 3 Department of Vaccines and Diagnostics, Moredun Research Institute, Edinburgh, EH26 0PZ UK; 4 Department of Disease Control, Moredun Research Institute, Edinburgh, EH26 0PZ UK; 5 University of Massachusetts Chan Medical School, Program in Molecular Medicine, Worcester, MA 01605 USA; 6 School of Immunity & Infection, University of Glasgow, Glasgow, G12 8TA UK; 7 Present Address: Department of Molecular Genetics, Faculty of Medicine, University of Toronto, Toronto, ON M5S 1A8 Canada; 8 Present Address: School of Immunity & Infection, University of Glasgow, Glasgow, G12 8TA UK; 9 Present Address: Cambridge Institute for Medical Research, University of Cambridge, Cambridge, CB2 0XY UK; 10 Present Address: Leiden University Center for Infectious Diseases, Leiden University Medical Center, Leiden, 2333ZA The Netherlands; 11 Present Address: Institute of Ecology and Evolution, School of Biological Sciences, The University of Edinburgh, Edinburgh, EH9 3FL UK; 12 Present Address: Cancer Research UK Scotland Institute, Switchback Road, Bearsden, Glasgow, G61 1BD UK

**Keywords:** Argonaute, Host–Pathogen, Extracellular RNA, RNA Interference, Nematode, Membranes & Trafficking, Microbiology, Virology & Host Pathogen Interaction, RNA Biology

## Abstract

Argonautes are ancient proteins with well-characterised functions in cell-autonomous gene regulation and genome defence, but less clear roles in non-cell-autonomous processes. Extracellular Argonautes have been reported across plants, animals and protozoa, yet their biochemical and functional properties remain elusive. Here, we demonstrate that an extracellular Argonaute (exWAGO) released by the rodent-infective nematode *Heligmosomoides bakeri* is detectable inside mouse cells during the natural infection. We show that exWAGO is released from *H. bakeri* in both vesicular and non-vesicular forms that have different resistances to proteolysis, different accessibilities to antibodies and associate with different subsets of secondary siRNAs. Using recombinant exWAGO protein, we demonstrate that non-vesicular exWAGO is internalised by mouse cells in vitro and that immunisation of mice with exWAGO confers partial protection against subsequent *H. bakeri* infection and generates antibodies that block exWAGO uptake into cells. Finally, we show that properties of exWAGO are conserved across Clade V nematodes that infect humans and livestock. Together, this work expands the context in which Argonautes function and illuminates an RNA-binding protein as a vaccine target for parasitic nematodes.

## Synopsis

A parasite-derived Argonaute protein, exWAGO, is secreted in association with siRNAs in vesicular and non-vesicular forms that both enter host cells. Vaccination with exWAGO confers partial protection against infection and generates antibodies that block non-vesicular exWAGO uptake by the host.

- The Clade V nematode *Heligmosomoides bakeri* secretes exWAGO in two forms that bind distinct siRNAs.
- exWAGO is internalised by mouse (host) cells in vivo.
- Vaccination with exWAGO reduces parasite burden and generates antibodies that block non-vesicular exWAGO uptake.
- exWAGO is highly conserved, abundantly expressed and secreted by parasitic nematodes that infect sheep and humans.

## Abstract (web summary)

A parasite-derived Argonaute protein, exWAGO, is secreted in vesicular and non-vesicular forms that enter host cells. Vaccination with exWAGO confers partial protection against infection and generates antibodies that block exWAGO uptake by the host.

## Graphical abstract

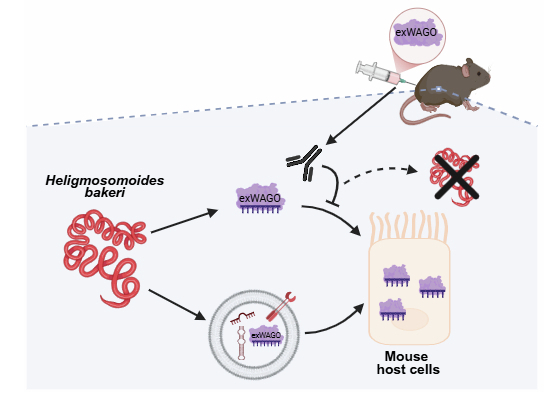

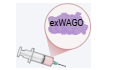

## Introduction

Argonautes are ancient proteins that work in partnership with small nucleic acid guides to regulate gene expression, control transposable elements (TEs) and defend cells against viruses (Swarts et al, 2014). In animals, there are two highly conserved clades of Argonautes: the AGO family that acts with microRNAs (miRNAs) or short interfering RNAs (siRNAs), and the PIWI family that acts with PIWI-interacting RNAs (piRNAs). Nematodes have evolved an additional clade of Argonautes, the Worm-specific Argonautes (WAGOs), which bind secondary siRNAs that are generally 22 nucleotides in length, start with a Guanosine (G) nucleotide and commonly have a 5’ triphosphate moiety due to their generation by RNA-dependent RNA polymerases (Gu et al, 2009; Seroussi et al, 2023; Yigit et al, 2006). Research in *Caenorhabditis elegans* suggests that WAGOs function to amplify or transmit an RNA interference (RNAi) response, for example, during environmental RNAi (amplifying responses against foreign RNA) or soma-to-germline transmission of RNAi (transgenerational inheritance) (Ketting and Cochella, 2020). However, many nematode species are parasitic, and how they might use Argonautes during infections inside diverse hosts has not been well explored (Buck and Blaxter, 2013).

In order to survive in their hosts, parasitic nematodes release excretory-secretory (ES) products that condition the host environment and immune response towards tolerance, also promoting chronic infections (Girgis et al, 2013; King and Li, 2018; Maizels, 2020). *Heligmosomoides bakeri* is a gastrointestinal nematode parasite of mice that serves as a model for intestinal worm infections, which are estimated to affect a quarter of the world’s human population (Reynolds et al, 2012; WHO, 2023) and are highly prevalent in domestic and wild animal species, causing substantial global health burdens and economic losses (Charlier et al, 2020). We previously showed that extracellular vesicles (EVs) are a component of *H. bakeri* ES (HES) and demonstrated that the parasite EVs have immune-suppressive properties in host cells (Buck et al, 2014; Coakley et al, 2017). We subsequently discovered that a specific WAGO protein (Hb-exWAGO) is present in these EVs in association with siRNAs (Chow et al, 2019). Yet whether EVs are the main carrier of siRNAs and exWAGO is not clear, nor is it known whether Hb-exWAGO is important during infection. Studies in plants have demonstrated that small RNA trafficking from fungal or oomycete pathogens into plant cells is important for pathogen survival and immune evasion (Qiao et al, 2023). Yet in these systems, the pathogen small RNAs hijack the Argonaute of the receiving host cell to function (Qiao et al, 2023). There is no example to date of an Argonaute from one species operating in another, despite a mounting body of literature across plants, protozoa and animals that Argonautes can be exported by cells in multiple forms (Arroyo et al, 2011; Chow et al, 2019; Garcia-Silva et al, 2013; Geekiyanage et al, 2020; He et al, 2021; Jeppesen et al, 2019; Karimi et al, 2022; Koch et al, 2025; McKenzie et al, 2016; Melo et al, 2014; Si et al, 2023; Zhang et al, 2019, 2021).

Here, we take advantage of vaccination as a method to target the environment-exposed form of Hb-exWAGO in *H. bakeri*. We show that vaccination with recombinant Hb-exWAGO significantly reduces parasite burden and that antibodies raised during infection block the uptake of non-vesicular Hb-exWAGO into cells in vitro. We also provide evidence of transfer of the Hb-exWAGO from nematode to mouse cells during the natural infection using immunohistochemistry. Finally, we show that the properties of exWAGO are conserved in Clade V parasitic nematodes that infect humans and livestock, including high expression in adult stages, presence in ES products and preference for binding secondary siRNAs. This work illuminates the functional capacity of an extracellular Argonaute in vivo and reveals a new vaccine candidate for an important and neglected class of pathogens where there are no vaccines for humans (Perera and Ndao, 2021), limited (and no recombinant) vaccine options in animals (Claerebout and Geldhof, 2020), and mounting resistance to anthelminthic drugs (Orr et al, 2019; Wit et al, 2021).

## Results

### Hb-exWAGO is released from *H. bakeri* in non-vesicular and vesicular forms that have distinct accessibilities to proteases and antibodies

**Figure 1:**
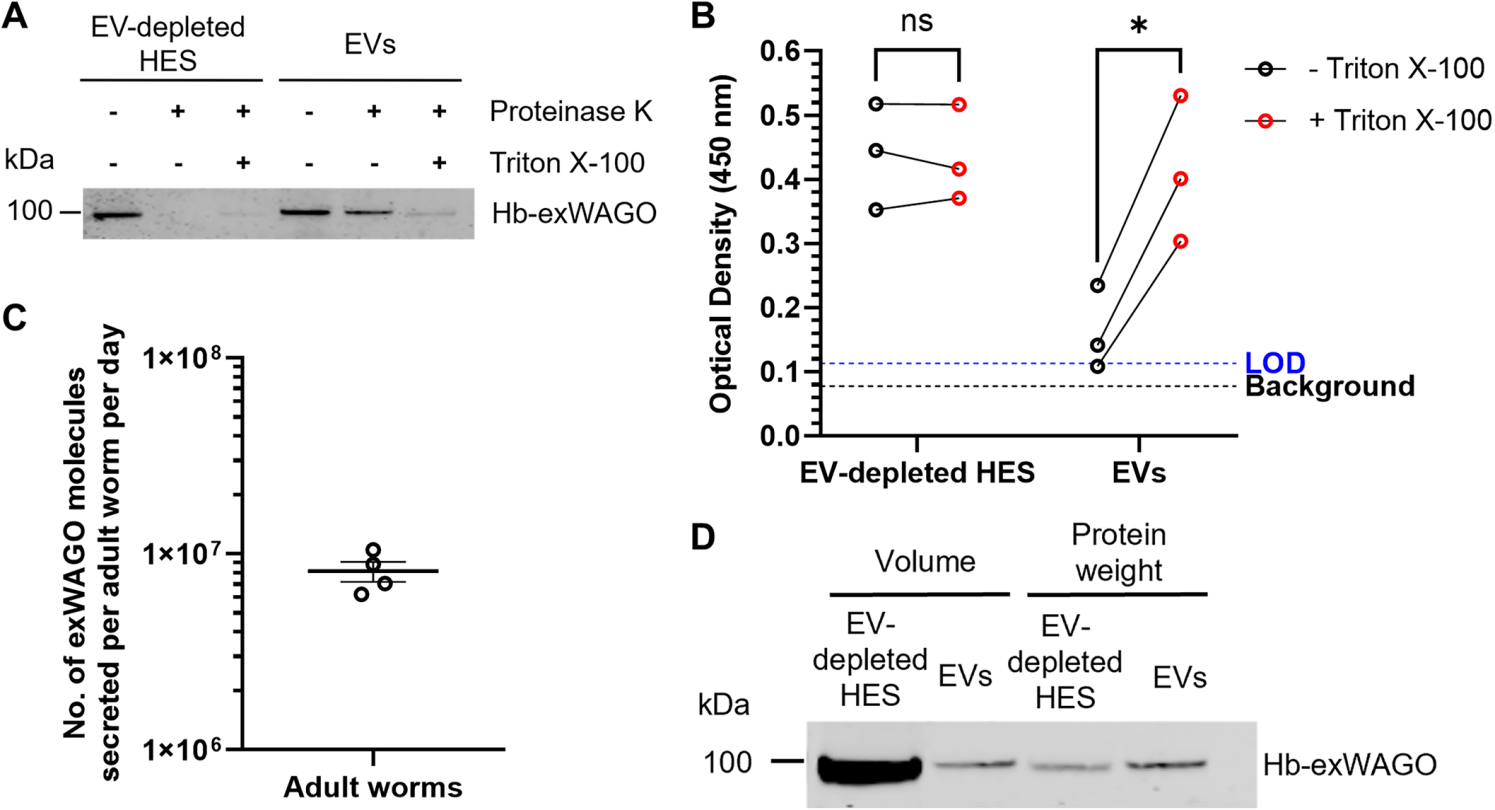
Hb-exWAGO is released in distinct vesicular and non-vesicular forms at high copy numbers. (**A**) Western blot of Hb-exWAGO in EV-depleted HES or EVs following incubation in the absence or presence of Proteinase K (5 µg/ml) and/or Triton X-100 (0.05%) as indicated with 2.0 µg total protein. (**B**) ELISA detection of Hb-exWAGO using equal protein amounts (0.2 µg) of EV-depleted HES or EVs in the presence or absence of Triton X-100 (0.05%) (*n* = 3 biological replicates). The limit of detection (LOD; blue dotted line) and background (no sample, black dotted line) are indicated. (**C**) Absolute copy number of Hb-exWAGO molecules present in HES, normalised per adult worm per day in culture based on a sample of 30 worms (mixed sex) in culture from 24 to 96 h post-harvest from mice (*n* = 4 biological replicates). (**D**) Western blot of Hb-exWAGO in EV-depleted HES and EVs following separation by ultracentrifugation from the same volume of HES starting material (protein yield from EV-depleted HES = 38.5 µg, EVs = 2.0 µg) or using equal protein quantities (2.0 µg of each). Data information: In (**B**), the data represent individual data points, and significance was determined using a two-way ANOVA (EV-depleted HES: *P* = 0.9588; EVs: *P* = 0.0271). (**C**) Data are presented as individual data points with the mean ± S.E.M. Source data are available online for this figure.

**Figure EV1:**
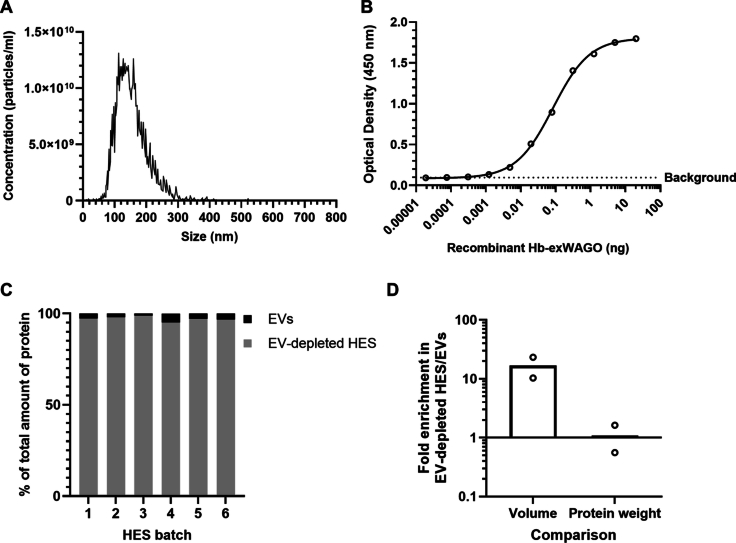
Quantification of exWAGO in EVs and EV-depleted HES. (**A**) Representative particle size distribution profile and quantification of *H. bakeri* EVs following ultracentrifugation. Data show the mean of *n* = 3 technical replicates. (**B**) Representative standard curve of Hb-exWAGO concentration based on ELISA using the recombinant Hb-exWAGO analysed using 4PL regression. Data show the mean of *n* = 2 technical replicates. (**C**) The amount of total protein quantified in EV-depleted HES and EVs following separation of HES by ultracentrifugation, shown as percentage of total protein of both fractions combined. Data are from biologically independent batches of HES collected from day 1–8 post-harvest of worms from mice (*n* = 6 biological replicates). (**D**) Fold-enrichment of Hb-exWAGO in EV-depleted HES compared to EVs following quantification of western blot band intensities. EV-depleted HES and EVs from the same starting material were analysed based on loading from equivalent starting sample volumes (left) or based on loading equivalent total protein (2.0 µg). Individual data points are shown with the mean (*n* = 2 biological replicates).

In order to isolate vesicular and non-vesicular forms of Hb-exWAGO, we used sequential centrifugation, filtration and ultracentrifugation of *H. bakeri* excretory-secretory products (HES). The expected yield and size of EVs were confirmed with Nanoparticle Tracking Analysis (Fig. EV1A), consistent with our previous reports (Buck et al, 2014; Simbari et al, 2016). Using western blot analysis, we found that Hb-exWAGO in EVs was largely protected from proteolysis following Proteinase K incubation in the absence of detergent, whereas Hb-exWAGO in EV-depleted HES was susceptible (Fig. 1A). We then developed an ELISA assay to test whether vesicular and non-vesicular Hb-exWAGO had different accessibilities to antibody capture. Using equal total protein inputs from EVs and EV-depleted HES, we detected an increase in the Hb-exWAGO signal when EVs were treated with detergent, whereas the Hb-exWAGO signal in EV-depleted HES remained unaffected (Fig. 1B). These data collectively demonstrate that Hb-exWAGO is present both inside and outside EVs, with each form having different protease sensitivities and antibody accessibilities. Using the ELISA assay in the presence of detergent with a recombinant Hb-exWAGO standard (Fig. EV1B), we further quantified that ~8.2 ± 1.0 × 10^6^ (mean ± S.E.M.) total copies of Hb-exWAGO are secreted per adult worm per day ex vivo (Fig. 1C). We note that EVs accounted for <5% of the total amount of protein found in HES products when purified by ultracentrifugation (Fig. EV1C), and western blot analysis confirmed that substantially more non-vesicular Hb-exWAGO is released by the parasites compared to vesicular Hb-exWAGO (Figs. 1D and  EV1D).

### Non-vesicular and vesicular Hb-exWAGO bind distinct subsets of secondary siRNAs

**Figure 2:**
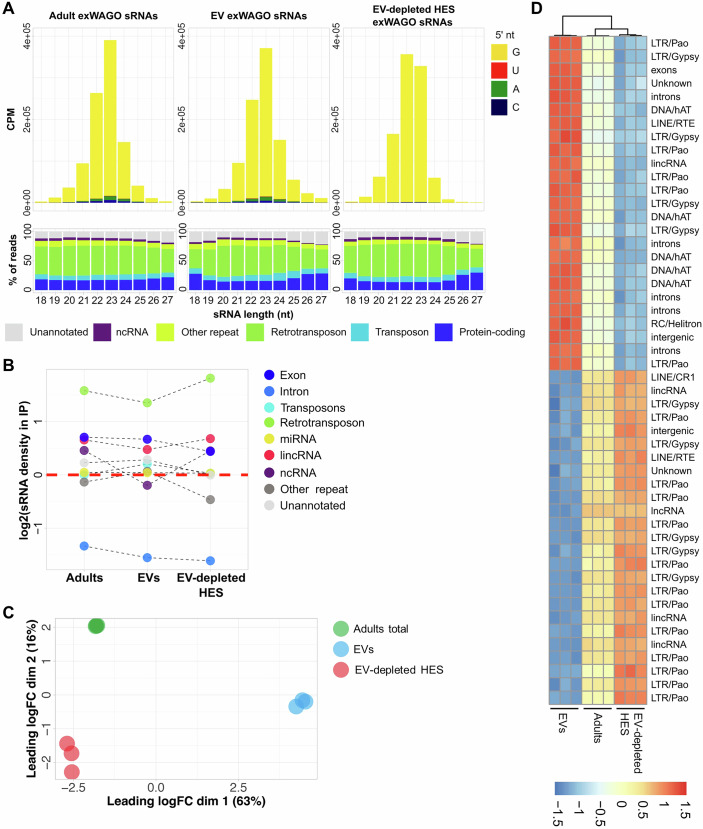
Non-vesicular and vesicular Hb-exWAGO bind distinct subsets of secondary siRNAs. (**A**) Top panels show the average length distribution and first nucleotide plots of sRNAs mapping to the *H. bakeri* genome following Hb-exWAGO immunoprecipitation from adult worms, EVs or EV-depleted HES (CPM = counts per million). Bottom panels show the percentage of sRNAs of each length that map to different annotated regions of the *H. bakeri* genome. (**B**) Hb-exWAGO preference for sRNAs derived from different annotations, corrected by the size of each annotation in the *H. bakeri* genome. The *y* axis shows the log_2_ (percent of mapped counts/annotated percent of genome). Uncorrected percentages are shown in Fig. EV2A for reference. (**C**) Multidimensional scaling (MDS) analysis of Hb-exWAGO sRNAs in adult worms, EVs and EV-depleted HES. Each dot represents an immunoprecipitation replicate, with distances related to the largest 500 log_2_(Fold-Change) values between each pair of dots. (**D**) Heatmap showing relative abundance (row-scaled Z-scores) for the 25 EV and 25 EV-dep HES regions with the highest average counts-per-million out of all differentially expressed (FDR < 1%) regions (highlighted in Fig. EV2B). Data information: All figures use the data from the same *n* = 3 biological replicates. Source data are available online for this figure.

**Figure EV2:**
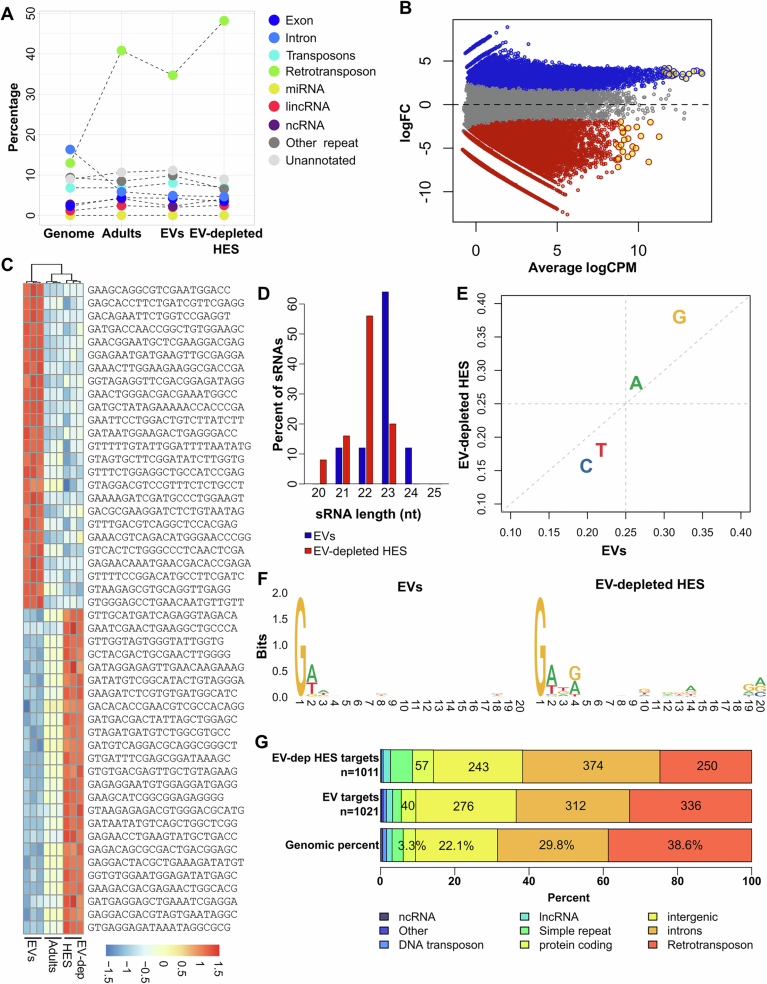
In-depth analysis of sRNAs bound to non-vesicular and vesicular exWAGO. (**A**) Average percentages of mapped reads assigned to each annotation category of the *H. bakeri* genome for adult, EV and EV-dep HES libraries. The first column shows the percentage of each annotation category in the genome, as a reference. (**B**) An MA plot representing the differential expression between vesicular and non-vesicular Hb-exWAGO forms. The *x* axis shows the average log_2_(CPM) and the *y* axis the log_2_(Fold-Change). Each dot represents an annotated region of the *H. bakeri* genome. The 10,778 regions with fold-changes significantly higher than 2 in the vesicular libraries are shown in dark blue; the 39,776 regions with fold-changes significantly higher than 2 in non-vesicular libraries are shown in dark red, all controlled for False-Discovery Rate < 1%; the 25 regions with highest CPM for each are highlighted in gold. (**C**) Heat map showing relative abundance (row-scaled Z-scores) for the sRNAs with the highest CPM from the 25 EV and 25 EV-dep HES regions highlighted in (**B**). (**D**) Length distribution, (**E**) total nucleotide frequency and (**F**) nucleotide preference across the first 20nt of the sRNAs shown in (**C**). (**G**) TargetFinder results using the first 20nt of the same sRNAs and the complete mouse genome, grouped according to annotation in the mouse genome of the predicted target sites. Genomic percent of the mouse genome is provided in the first column as a reference. There were 1021 target sites with penalty score <= 3 for the top 25 EV sRNAs, and 1011 for the top 25 EV-dep HES sRNAs. Data information: All figures use the data from the same *n* = 3 biological replicates.

We then carried out immunoprecipitation of Hb-exWAGO to determine whether both the vesicular and non-vesicular forms bind small RNAs. As shown in Fig. 2A (upper panel), Hb-exWAGO isolated from adult worms, EVs or EV-depleted HES all bind to small RNAs that are largely 22–23 nucleotides (nt) in length and start with a Guanosine, features that are consistent with secondary siRNAs (Chow et al, 2019). Consistent with our previous results using qRT-PCR of sRNAs bound to Hb-exWAGO in EVs, the majority of Hb-exWAGO-bound sRNAs derive from transposable elements (Chow et al, 2019) (Fig. 2A, lower panel). Since this could at least in part be explained by the high transposon content of the *H. bakeri* genome (Stevens et al, 2023), we performed a more in-depth analysis to address Hb-exWAGO binding preferences. After removal of non-specifically bound RNA (see Methods) and correcting for annotation coverage in the genome, we found that Hb-exWAGO preferentially binds to sRNAs derived from retrotransposons, followed by protein-coding genes and lncRNAs (Figs. 2B and  EV2A). Despite these similarities, when counting the number of sRNAs mapped to each genomic loci, vesicular and non-vesicular Hb-exWAGO forms are distinct, with the first dimension of an MDS (explaining ~63% of the variation) separating the vesicular replicates from adult and non-vesicular forms (Fig. 2C). To find which loci are related to this difference we performed a differential expression analysis (DEA) comparing vesicular and non-vesicular forms. Out of 70,564 queried genomic loci, 10,778 produce more Hb-exWAGO-bound sRNAs in the vesicular, while 39,776 produce more sRNAs in the non-vesicular form (False-Discovery Rate <1%, Fig. EV2B). Although transposons and retrotransposons as a whole produce similar amounts of sRNAs for these two Hb-exWAGO forms (Fig. 2A), individual copies (particularly of LTR/Pao, LTR/Gypsy and DNA/hAT−Tip100 superfamilies) are associated with one or the other (Fig. 2D; Dataset EV1). Even though the DEA was not performed for individual sRNA sequences, the most abundant sRNAs that map to each region are also more abundant in the expected Hb-exWAGO form (Fig. EV2C; Dataset EV1).

We next asked if any properties of the sRNA sequences correlate with the two distinct Hb-exWAGO forms. Already visible in the raw sequencing data (Figs. 2A and  EV2D), the non-vesicular form of Hb-exWAGO is bound to slightly shorter sequences which could reflect higher susceptibility to trimming of the sRNA outside of the EVs. We also note a minor bias in nucleotide composition with non-vesicular Hb-exWAGO sRNAs having more purines (G, A) than the vesicular Hb-exWAGO sRNAs (Fig. EV2E) but both showing a clear enrichment in a “GA” motif at the 5’ end (Fig. EV2F). Using TargetFinder (Fahlgren and Carrington, 2010) we find that both vesicular and non-vesicular Hb-exWAGO sRNAs have similar numbers of high-complementarity sites in the mouse genome including in retrotransposons and protein-coding genes (Fig. EV2G). Collectively, these data demonstrate that Hb-exWAGO exists in both vesicular and non-vesicular forms, that these are not contaminants of one another, and that both forms are released in complex with distinct populations of sRNAs.

### Hb-exWAGO is detected inside host cells in vivo and internalised by live cells in vitro

**Figure 3:**
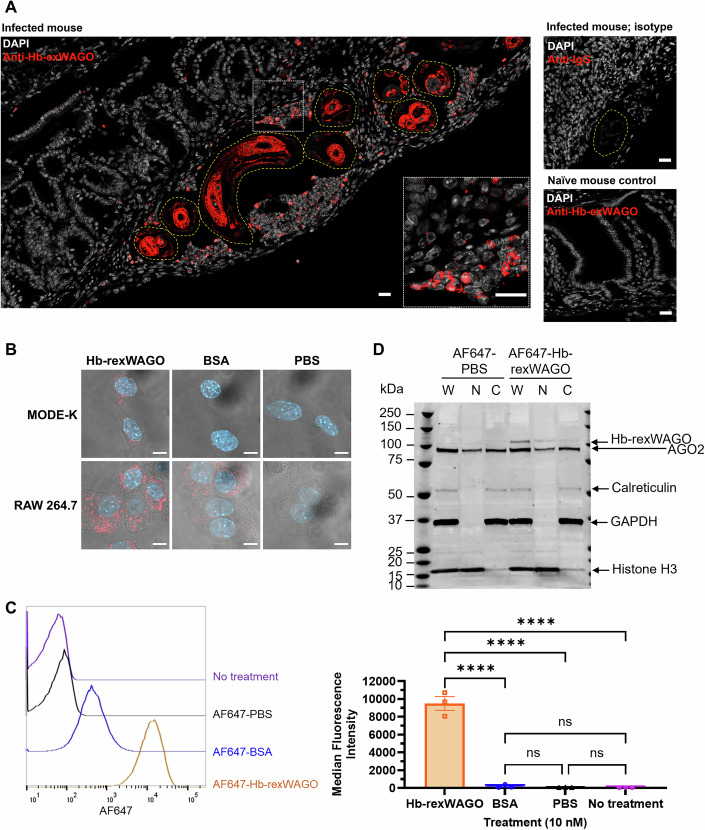
Detection of native Hb-exWAGO inside host cells in vivo and internalisation of recombinant Hb-exWAGO by live host cells in vitro. (**A**) Confocal microscopy images of mouse gut tissue 7 days post infection with *H. bakeri* compared to uninfected gut tissue. Overlay of anti-Hb-exWAGO or IgG isotype control staining (red) and DAPI (white). The outline of parasites is indicated with yellow dotted lines. White box indicates zoomed-in sections. Scale bars = 20 µm. (**B**) Confocal microscopy images of MODE-K and RAW 264.7 cells following incubation with 0.1 µM AF647-labelled recombinant Hb-exWAGO (Hb-rexWAGO), BSA or PBS for 4 h. Overlay of AF647 signal (red), DAPI (blue), and brightfield. Scale bars = 10 µm. (**C**) The Median Fluorescence Intensity (MFI) of the AF647 signal detected by flow cytometry analysis of MODE-K cells treated with 10 nM of AF647-labelled recombinant Hb-exWAGO (Hb-rexWAGO), BSA, PBS, or no treatment for 4 h. (**D**) Western blot analysis of MODE-K cells treated with 0.1 µM AF647-labelled Hb-rexWAGO or PBS for 4 h followed by subcellular fractionation. The blot shows the whole-cell lysate (W), nuclear (N) and cytoplasmic (C) fractions. GAPDH and Calreticulin are used as cytoplasmic markers and Histone H3 as a nuclear marker. Endogenous mouse AGO2 is also shown. Data information: In (**A**–**C**, left) data are representative of three biological replicates. In (**C**, right), individual data points are shown with the mean ± S.E.M. (*n* = 3; biological replicates) and were analysed using a repeated measures one-way ANOVA (Hb-rexWAGO vs BSA: *P* = 0.000008; Hb-rexWAGO vs PBS: *P* = 0.000007; Hb-rexWAGO vs no treatment: *P* = 0.000007; BSA vs PBS: *P* = 0.994101; BSA vs no treatment: *P* = 0.989276; PBS vs no treatment: *P* = 0.999928). Source data are available online for this figure.

**Figure EV3:**
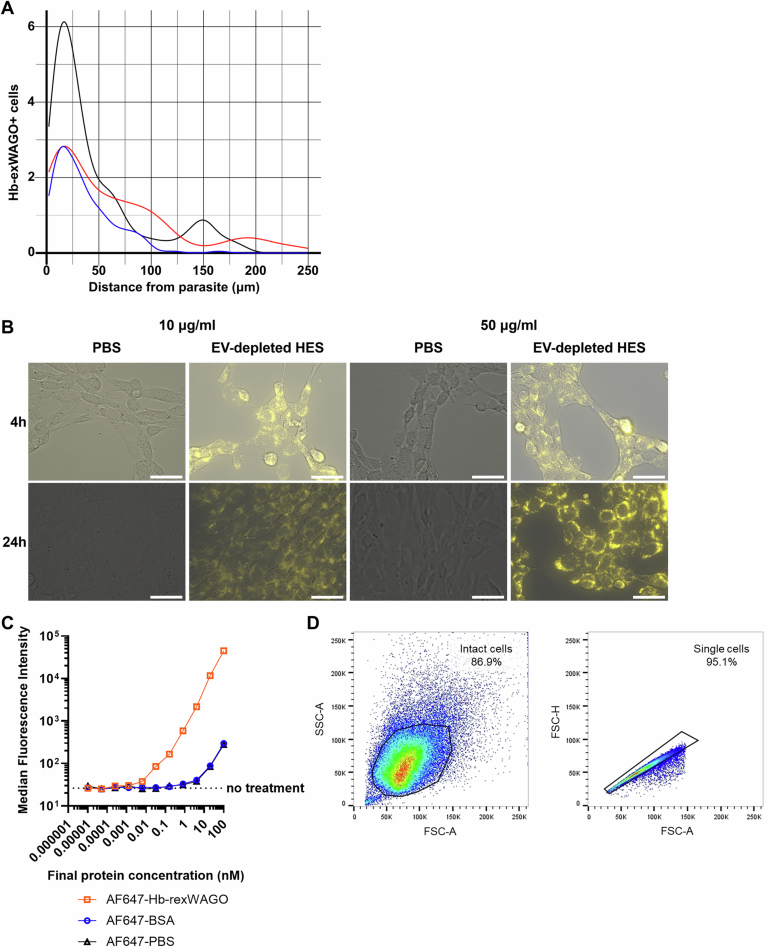
Detection of exWAGO in vivo and fluorescently-labelled proteins internalised by MODE-K cells. (**A**) Density plot of the distance calculated between Hb-exWAGO positive cells and the parasite (red and black lines are technical replicates; blue line is a biological replicate). (**B**) Brightfield and fluorescence composite microscopy images from MODE-K cells incubated with 10 or 50 µg/ml Cy-5-labelled EV-depleted HES or Cy-5-labelled PBS for 4 or 24 h. Scale bars = 50 µm. Data are representative of 2 biological replicates. (**C**) Dose response of AF647-labelled recombinant Hb-exWAGO (Hb-rexWAGO), BSA or PBS concentrations incubated with MODE-K cells for 4 h measured by flow cytometry (*n* = 1). (**D**) Gating strategy applied for selecting intact cells (left panel) followed by selection of single cells (right panel) applied for flow cytometry analysis.

The above data suggest that the adult worms release large quantities of Hb-exWAGO ex vivo. To test whether Hb-exWAGO is also released in vivo, we used immunohistochemistry of paraffin-embedded *H. bakeri*-infected mouse gut sections at 7 days post infection. Hb-exWAGO is detected inside the parasite, and also inside host immune cells that surround the worm (Fig. 3A) with the highest proportion of Hb-exWAGO positive cells being in close proximity to the worm (Fig. EV3A). These data show that substantial quantities of Hb-exWAGO are detected in vivo and inside host cells, but do not distinguish which form of Hb-exWAGO, vesicular and/or non-vesicular, is transmitted to host cells.

While we and others have shown uptake of parasite EVs by host cells, there is limited data on the capacity of non-vesicular nematode parasite proteins to enter live host cells, and no examples to date in this parasite model. We therefore first labelled EV-depleted HES with Cy-5 NHS Ester dye and tested whether EV-depleted HES proteins are internalised by mouse intestinal enterocyte epithelial MODE-K cells (Vidal et al, 1993). Signal was detected by 4 h post incubation and remained strong at 24 h with accumulation in the cytoplasm (Fig. EV3B). We then tested whether the recombinant Hb-exWAGO (Hb-rexWAGO) itself could be internalised. We compared uptake in the MODE-K cell line to a macrophage RAW 264.7 cell line expected to phagocytose extracellular material and included labelled bovine serum albumin (BSA) protein for comparison. As shown in Fig. 3B, we detect internalisation of recombinant Hb-exWAGO by both MODE-K and RAW 264.7 cells, whereas BSA is only internalised by the RAW 264.7 cell line. We further developed a Flow Cytometry assay with MODE-K cells to measure recombinant Hb-exWAGO uptake down to picomolar concentrations (Fig. EV3C). As shown in Fig. 3C, there is a clear shift in the Median Fluorescence Intensity in cells incubated with AF647-labelled Hb-rexWAGO and near-background signal from the AF647-labelled BSA and PBS controls (Figs. 3C and EV3D). We note that cells were trypsinised to ensure any signal detected comes from internalised labelled proteins and not those that could be associated to the surface. The internalisation of Hb-rexWAGO into MODE-K cells was also confirmed by fractionation of the cell lysate and western blot following incubation with AF647-labelled recombinant Hb-exWAGO. As shown in Fig. 3D, Hb-rexWAGO is detected in both cytoplasmic and nuclear fractions, similar to what is found for endogenous mouse AGO2. Taken together, our results indicate that large quantities of Hb-exWAGO are released by adult parasites and are detected inside host cells in vivo, and the non-vesicular form of Hb-exWAGO is able to enter multiple cell types in vitro, including epithelial cells.

### Vaccination with recombinant Hb-exWAGO confers partial protection against subsequent infection and generates antibodies that block Hb-exWAGO uptake into cells

To understand whether the non-vesicular form of Hb-exWAGO might be important during infection, we took advantage of the capacity to target this protein through vaccination with recombinant Hb-exWAGO. We used, as a benchmark, vaccination with total HES, which provides a cocktail of >360 proteins naturally released by the parasite (Buck et al, 2014; Hewitson et al, 2015). HES vaccination in mice was previously shown to result in sterile immunity (i.e. the parasite has been eliminated prior to reproduction in the host) with the observed effects attributed to both IgG1 antibody-mediated and Type 2 cellular responses (Hewitson et al, 2015).

**Figure 4:**
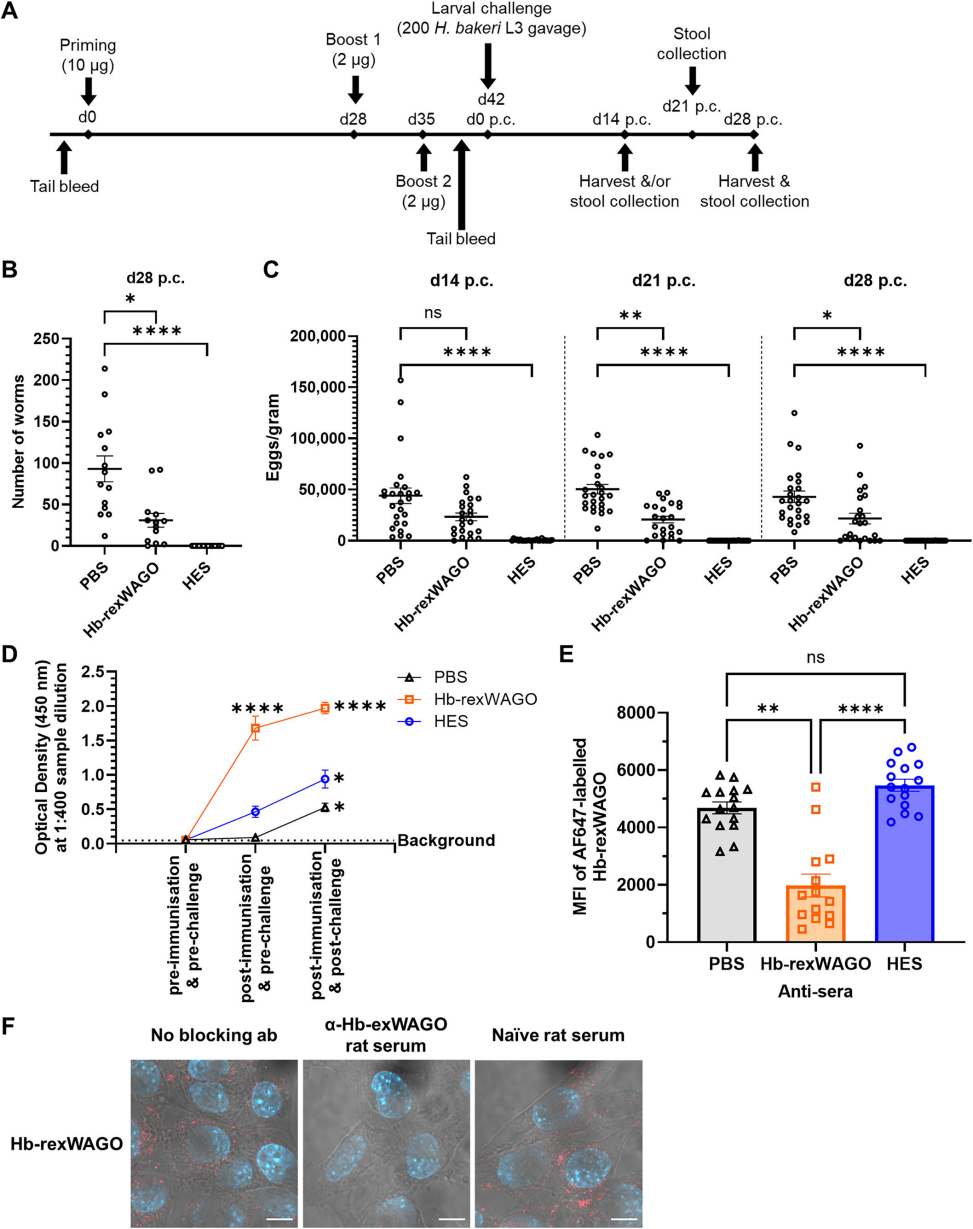
Vaccination with Hb-exWAGO generates partial protection against infection and generates antibodies that block Hb-exWAGO internalisation by mouse cells. (**A**) Schematic of the immunisation timeline. Mice were vaccinated with recombinant Hb-exWAGO (Hb-rexWAGO), HES, or PBS and then challenged with 200 L3-stage *H. bakeri* larvae. Post-challenge is denoted as p.c. (**B**) The number of adult worms 28 days post-challenge (p.c.) recovered in the small intestine following vaccination of mice and challenge with 200 L3 stage larvae (*n* = 13–14 mice per vaccination group). (**C**) The number of eggs per gram of faeces at 14, 21 and 28 days post-challenge (p.c.) (*n* = 23–25 mice per vaccination group). (**D**) ELISA detection of exWAGO-specific IgG1 antibodies in sera obtained from vaccinated mice pre-immunisation and pre-challenge (pooled serum from 4 to 5 mice per vaccination group, from one experiment), post-immunisation and pre-challenge (4–6 days after the second boost), and post-immunisation and 28 days post-challenge (*n* = 9–15 mice per vaccination group). Data show the mean optical density at 1:400 sample dilution ± S.E.M. (**E**) The Median Fluorescence Intensity (MFI) of AF647-labelled Hb-exWAGO in MODE-K cells treated with 10 nM labelled protein for 4 h and sera (1:250 dilution) from vaccinated mice 28 days post-challenge (*n* = 14–15 mice per vaccination group). (**F**) Confocal super-resolution microscopy images of MODE-K cells incubated with 0.1 µM AF647-labelled Hb-rexWAGO for 4 h in the presence of rat anti-exWAGO or naive serum (1:150 dilution). Overlay of AF647 signal (red), DAPI (blue), and brightfield. The brightfield channel only was adjusted per condition. Data are representative of three biological replicates. Scale bars = 10 µm. Data information: In (**B**, **C**, **E**), individual data points are shown with the mean ± S.E.M. Significance was determined using an unpaired Kruskal–Wallis test. For (**B**), PBS vs Hb-exWAGO worm counts d28 p.c.: *P* = 0.0168092; PBS vs HES worm counts d28 p.c.: *P* = 0.0000001, for (**C**) PBS vs Hb-rexWAGO egg counts d14 p.c: *P* = 0.1058407, d21 p.c.: *P* = 0.0041616, d28 p.c.: *P* = 0.0.0112558; PBS vs HES egg counts d14, d21 and d28 p.c.: *P *< 0.0000001, for (**E**) PBS vs HES: *P* = 0.252510, PBS vs Hb-rexWAGO: *P* = 0.005555, HES vs Hb-rexWAGO: *P* = 0.000005. (**D**) Significance was determined using a one-way ANOVA (post-immunisation & pre-challenge PBS vs post-immunisation & post-challenge PBS: *P* = 0.0168611; post-immunisation & post-challenge PBS vs post-immunisation & post-challenge HES: *P* = 0.0118812; post-immunisation & post-challenge HES vs post-immunisation & post-challenge Hb-rexWAGO and post-immunisation & pre-challenge Hb-rexWAGO vs post-immunisation & pre-challenge HES: *P* < <0.0000001). In (**B**–**E**), all data are pooled from three biologically independent experiments unless otherwise stated. Source data are available online for this figure.

**Figure EV4:**
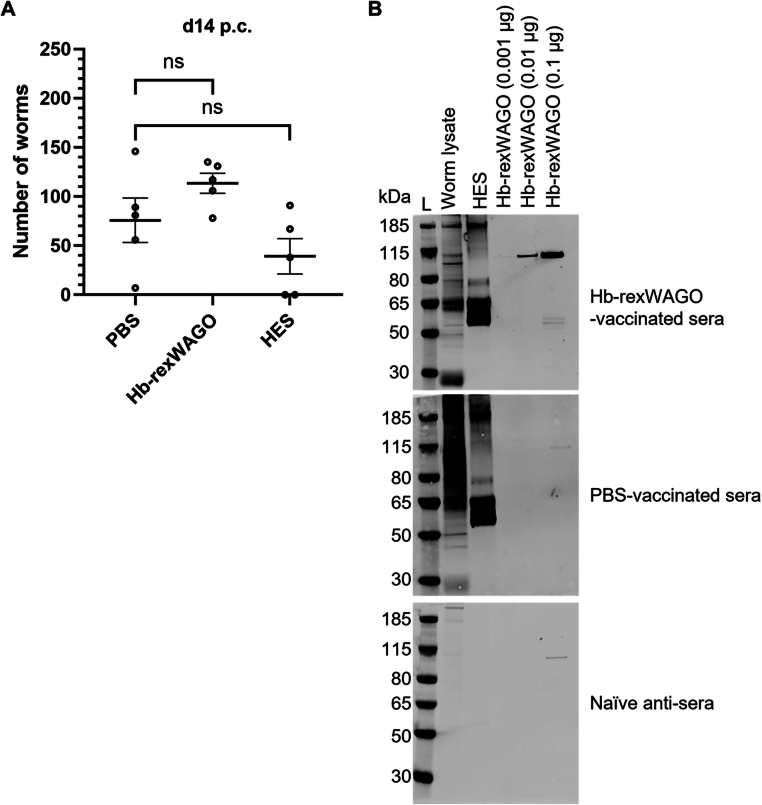
Parasite and serological responses to vaccine. (**A**) The number of adult worms 14 days post-challenge (p.c.) recovered in the small intestine following vaccination of mice with recombinant Hb-exWAGO (Hb-rexWAGO), HES, or PBS and challenge with 200 L3 stage larvae (*n* = 5 mice per vaccination group, from one experiment). (**B**) Western blot analysis of IgG responses using pooled sera from Hb-exWAGO- or PBS-vaccinated mice 28 days post-challenge (pool is from *n* = 5 mice in one experiment), or unvaccinated and uninfected mouse (referred to as naïve anti-sera, *n* = 1 mouse). Pooled sera were used as the primary antibody (1:1000 in 5% BSA/TBST) and blots were probed with goat anti-mouse IgG AF680 antibody (1:10,000 in 5% BSA/TBST). Adult worm lysate = 1 µg; HES = 1 µg; recombinant Hb-exWAGO = 0.001–0.1 µg, L = ladder. Data information: In (**A**), individual data points are shown with the mean ± S.E.M. Data were analysed using an unpaired Kruskal–Wallis test (PBS vs Hb-rexWAGO: *P* = 0.3814154; PBS vs HES: *P* = 0.2045549).

Mice were immunised with recombinant Hb-exWAGO, PBS (negative control) or HES (positive control) in Imject Alum adjuvant followed by challenge with *H. bakeri* L3 stage larvae (Fig. 4A). As expected from previous studies (Coakley et al, 2017; Hewitson et al, 2015), vaccination with HES confers sterile immunity as seen by a dramatic drop in the number of eggs per gram of faeces at 14 days post-challenge and clearance of worms by day 28 post-challenge compared to mice vaccinated with PBS (Figs. 4B,C and  EV4A). Strikingly, vaccination with recombinant Hb-exWAGO resulted in an average decrease of 67% in adult worm burdens compared to the PBS group at day 28 post-challenge (Fig. 4B). Consistent with the reduction in worm burden, there were also decreased egg burdens in the Hb-exWAGO-vaccinated group (Fig. 4C). Serum antibody responses show that vaccination with recombinant Hb-exWAGO elicited IgG and, in particular, IgG1 antibodies specific to Hb-exWAGO (Figs. 4D and  EV4B), consistent with known effects of alum adjuvant (Boyaka, 2017). Immunisation with HES also induced generation of Hb-exWAGO-specific IgG1 antibodies, as expected since Hb-exWAGO is present in HES, but these are much lower than in the Hb-exWAGO vaccine (Fig. 4D). Interestingly, we detected Hb-exWAGO-specific IgG1 antibodies post-challenge in mice immunised with PBS indicating that (at least some of) the Hb-exWAGO protein becomes accessible to the immune system via the natural secretion of Hb-exWAGO by the parasites and that lab mice naturally generate a low level of Hb-exWAGO-specific antibodies during infection, but this is significantly boosted by the vaccine (Fig. 4D).

To test whether antibodies could inhibit Hb-exWAGO uptake by cells, we used the Flow Cytometry assay with labelled recombinant Hb-exWAGO. As shown in Fig. 4E, treatment of MODE-K cells with sera from Hb-exWAGO vaccinated mice post-challenge inhibited uptake of AF647-labelled Hb-exWAGO compared to sera from mice vaccinated with HES and PBS. Reduction in the uptake of labelled Hb-exWAGO was also observed by confocal microscopy following treatment of MODE-K cells with anti-Hb-exWAGO antibodies compared to isotype controls (Fig. 4F). These data suggest an antibody-exposed form of Hb-exWAGO is important in vivo and demonstrate that vaccination generates functional antibodies against Hb-exWAGO that block its uptake into host cells.

### ExWAGO is secreted across Clade V nematodes that infect livestock and humans and shows conserved features in its association with 22 G siRNAs

**Figure 5:**
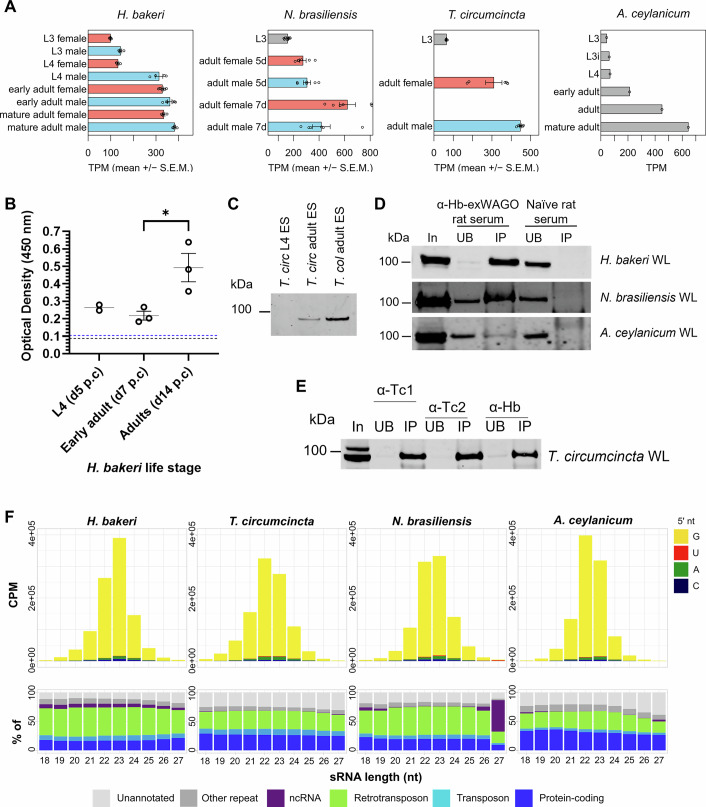
ExWAGO is highly expressed in adult worms across Clade V nematodes that infect livestock and humans and binds 22–23G small RNAs. (**A**) Expression levels of exWAGO at different developmental stages based on RNAseq data in *H. bakeri* (Data ref: Pollo et al, 2023) (*n* = 4–7 biological replicates per group), *N. brasiliensis* (Data ref: Ferguson et al, 2023; Data ref: Chandler et al, 2017) (*n* = 6–9 biological replicates per group), *T. circumcincta* (Data ref: NIH Bioproject PRJEB7677, 2015) (*n* = 6 biological replicates per group) and *A. ceylanicum* (Data ref: Schwarz et al, 2015) (*n* = 1 per group). Grey = no sex defined; red = female; blue = male; TPM = transcripts per million. Individual data points are shown with the mean ± S.E.M. where *n* > 2. (**B**) ELISA detection of Hb-exWAGO in HES collected from *H. bakeri* across different life stages in the presence of Triton X-100 detergent (0.05%). Data show the individual data points with the mean optical density (*n* = 2–3 biological replicates). Error bars represent the S.E.M., where *n* > 2. The Limit of Detection (LOD; dotted blue line) and background (no sample, black dotted line) are indicated. (**C**) Western blot analysis of exWAGO from the ES of *T. circumcincta* (*T. circ*) L4 and adults, and *T. colubriformis* (*T. col*) adults, probed using anti-*H. bakeri*-exWAGO antibody (6.0 µg lysate loaded per sample). (**D**) Western blot of exWAGO immunoprecipitations (IPs) using rat anti-Hb-exWAGO serum or naïve rat serum from worm lysates (WL) of *H. bakeri* (200 µg), *N. brasiliensis* (25 µg) and *A. ceylanicum* (25 µg). For each IP the volumes loaded were as follows: *H. bakeri* input (In) = 1%, unbound (UB) = 1%, eluate (IP) = 5% by volume; *N. brasiliensis* and *A. ceylanicum* In = 32%, UB = 2%, IP = 37.5% by volume. (**E**) Western blot of exWAGO immunoprecipitations using rat anti-Tc-exWAGO (α-Tc1 or α-Tc2) or anti-Hb-exWAGO serum from *T. circumcincta* (150 µg) worm lysate (WL). (In = 6.7%, UB = 2%, IP = 10% by volume). (**F**) Top: length distribution and first nucleotide (nt) plots of small RNA reads mapping to each parasite genome following exWAGO immunoprecipitation, polyphosphatase treatment and small RNA sequencing from *H. bakeri*, *N. brasiliensis*, *T. circumcincta* and *A. ceylanicum* adult worms. Data show the average length distributions (*H. bakeri*: *n* = 3 biological replicates; *N. brasiliensis*: *n* = 2 biological replicates; *T. circumcincta*: *n* = 2 biological replicates; *A. ceylanicum*: *n* = 2 technical replicates) (CPM = counts per million). Bottom: percentage of reads that map to different annotated regions of each parasite genome. Data information: (**B**) Significance was determined using an unpaired *t* test (*P* = 0.031819). Source data are available online for this figure.

A notable feature of Hb-exWAGO is its high conservation across Clade V gastrointestinal nematodes that infect livestock and humans (spanning 65–81% amino acid identity, Dataset EV2). To examine whether these exWAGO orthologues have similar properties to Hb-exWAGO, we first examined life stage expression of the exWAGO orthologues using publicly available transcriptome data from *H. bakeri*, rodent-infective *Nippostrongylus brasiliensis*, ruminant-infective *Teladorsagia circumcincta* and human-infective *Ancylostoma ceylanicum* nematodes. As shown in Fig. 5A and Dataset EV3, exWAGO expression is high in all the adult parasites, with lower levels in most larval stages across the four species. Using the ELISA assay, we also found that *H. bakeri* adults (d14 p.c.) release more Hb-exWAGO than larvae (d5 p.c.) and early adults (d7 p.c.) when using the same volume of ES collected from equal numbers of larvae or adults, as shown in Fig. 5B. Western blot analysis of ES from *T. circumcincta* also showed that more exWAGO is found in ES from adults compared to larvae (Fig. 5C). Furthermore, we detected exWAGO in the ES of the ruminant- and human-infective worm *Trichostrongylus colubriformis* and found it to be present in previously published proteomic analysis of the ES of these parasites (Fig. 5C; Dataset EV4) (Chow et al, 2019; Rooney et al, 2022; Uzoechi et al, 2023). Collectively, our data suggest that exWAGO is an abundant secreted protein expressed across the parasite developmental stages and is most highly expressed in adults.

**Figure EV5:**
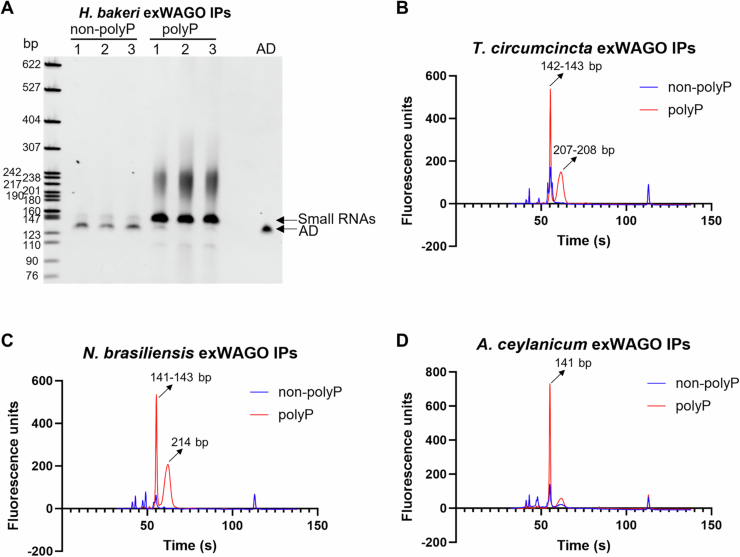
Analysis of small RNA library profiles treated with or without 5’ RNA polyphosphatase indicates exWAGO guides are 5’ triphosphorylated. (**A**) Size profile of the small RNA libraries generated following Hb-exWAGO immunoprecipitation from *H. bakeri* adult worms prior to size selection based on TBE 10% gel. 1–3 denote biological replicates. AD = adapter dimers; non-polyP = non-polyphosphatase treated; polyP = polyphosphatase treated. (**B**–**D**) Representative Bioanalyser High Sensitivity DNA analysis of the small RNA libraries following exWAGO immunoprecipitation from adult (**B**) *T. circumcincta* (*n *= 1 for non-polyP and *n* = 2 biological replicates for polyP), (**C**) *N. brasiliensis* (*n* = 2 biological replicates) and (**D**) *A. ceylanicum* (*n* = 1) worms. The data show the mean fluorescence units where replicates were available. The products enriched during polyphosphatase treatment are indicated. Non-polyP = non-polyphosphatase treated; polyP = polyphosphatase treated.

Given the high sequence conservation of exWAGO orthologues in these parasite species, we tested whether antibodies generated against recombinant Hb-exWAGO could capture exWAGO in the other parasites. Using western blot analysis, we show that exWAGO was readily immunoprecipitated from *A. ceylanicum*, *N. brasiliensis* and *T. circumcincta* (Fig. 5D,E). We then compared the profile of sRNAs bound to exWAGO in each species using small RNA sequencing and found that the sRNA profiles are extremely similar, showing the expected Guanosine as the first nucleotide, a length of 22–23 nucleotides and a propensity for mapping to transposable elements (Fig. 5F). Comparison between polyphosphatase-treated and untreated libraries also showed an increase in the amount of sRNAs ligated to the adaptors in response to 5’ RNA polyphosphatase treatment during the library preparation (Fig. EV5), consistent with the presence of a 5’ triphosphate group. Taken together, these data strongly point towards functional conservation of exWAGO orthologues in Clade V parasitic nematodes.

## Discussion

This work provides the first evidence that an Argonaute protein (assumed mainly to operate inside of cells) is a tractable vaccine candidate and suggests the non-vesicular form of exWAGO is functionally relevant during nematode parasite infections. Vaccine development remains challenging for parasitic nematodes, given the complex life cycles of nematodes and the numerous mechanisms for immunomodulation that they have co-evolved with their hosts over millions of years (Perera and Ndao, 2021). There are currently only two licensed anti-nematode vaccines for animal use, both of which are limited in scalability and rely on native antigens or irradiated larvae (Claerebout and Geldhof, 2020); there are no licensed vaccines for humans despite growing resistance to anthelmintic drugs (Perera and Ndao, 2021). One strategy in helminth vaccines is to target the proteins that the parasites use to modulate the immune system (Maizels, 2021). Work by ourselves and others has demonstrated that secreted RNAs are one type of immune evasion strategy used by nematodes (Buck et al, 2014; Ding et al, 2019; Liu et al, 2019; Tran et al, 2021), and we previously showed that Hb-exWAGO is released in complex with secreted siRNAs in EVs (Chow et al, 2019). Here we show a large proportion of Hb-exWAGO is released in a distinct non-vesicular form and that vaccination against this protein reduces subsequent burdens of infection (67% reduction in adult worm burdens). As a single recombinant protein, this is substantial compared to existing vaccines, which often use a cocktail of antigens (Britton et al, 2020), and its application could also be envisioned in multivalent strategies. For example, combining the exWAGO antigen with a surface antigen from EVs (Coakley et al, 2017) would be expected to block both vesicular and non-vesicular forms of exWAGO and its associated sRNAs from entering cells.

We hypothesise that once internalised by host cells, Hb-exWAGO-associated sRNAs modify host gene expression and alter the host environment to benefit the parasite, and that the efficacy of the vaccine is based on blocking non-vesicular Hb-exWAGO from carrying out this function. Consistent with this model, we show with immunohistochemistry that Hb-exWAGO is detected inside host cells during the infection and antibodies generated by the vaccine block Hb-exWAGO uptake into host cells. It is possible that some of the efficacy of the vaccine relates to recruitment of immune cells to sites of nematode presence; however, we note that many immunogenic HES proteins do not hold efficacy as vaccines.

Based on its higher expression and release by adults, we speculate that Hb-exWAGO plays an important role in the chronic infection stage and may contribute to parasite alteration of the epithelium, but it could also act earlier. The sRNAs bound to Hb-exWAGO have numerous sites of high complementarity to the mouse genome and many of the predicted targets fall within protein-coding genes but also other genetic elements, including TEs. Further biochemical understanding of Hb-exWAGO localisation and target interactions inside cells is needed to validate the specific genes and pathways targeted by vesicular Hb-exWAGO and non-vesicular Hb-exWAGO sRNAs. The literature around inter-species RNA-RNA interactions has focused on parasite sRNAs interacting with host protein-coding genes (Qiao et al, 2023); however, we do not rule out that host-encoded TEs or their remnants (within protein-coding genes or regulatory elements) could be targeted by the parasite sRNAs. In *C. elegans*, WAGOs can mediate gene expression at both transcriptional and post-transcriptional levels (Ketting and Cochella, 2020), and still little is known about the mechanism of gene silencing of the closest orthologs of Hb-exWAGO in *C. elegans*: SAGO-1, SAGO-2 and PPW-1 (30% amino acid identity, Dataset EV2). In mammals, most research on AGOs focuses on their roles in post-transcriptional gene regulation in the cytoplasm through interactions with 3’UTRs, however AGOs can also act in the nucleus and this might be particularly important during stress and infection conditions (Johnson and Corey, 2023; Ressel et al, 2024). We note that the Hb-exWAGO does not contain the catalytic DEDD/H tetrad involved in RNA slicing, and it is therefore expected to act in association with other proteins.

The dialogue around extracellular Argonautes has primarily focused on whether Argonautes are genuinely packaged within EVs or represent contaminants that co-purify (for example, if associated with the surface of the EV) (Weaver and Patton, 2020). We address this point specifically here with assays showing that Proteinase K sensitivity and antibody accessibility of the vesicular Hb-exWAGO occur only in the presence of detergent (in contrast to the non-vesicular form). We also show that the two forms of Hb-exWAGO bind to distinct subsets of sRNAs. A key finding from this study is that non-vesicular extracellular Argonautes (in addition to those within EVs) can also enter cells. A debate in the exRNA field is whether sufficient copies of exRNAs enter cells. Using a quantitative assay, we demonstrate that substantial (~10^7^) copies of Hb-exWAGO are released by each parasite per day, and our data suggest most of these copies derive from the non-vesicular form.

It will be of interest to understand the extent to which different Argonautes can enter different cells; at present, it is not clear if this is a property specific to exWAGO. The mechanism(s) by which non-vesicular Hb-exWAGO enters cells remains unknown; there is only one study in this area that reports the human AGO2 can be internalised into recipient cells via the receptor neuropilin-1 (Prud’homme et al, 2016). Why the parasite releases two forms of Hb-exWAGO is not clear; we show that Hb-exWAGO from EVs versus EV-depleted HES bind sRNA populations derived from different locations in the nematode genome, both of which are distinct from the sRNA population bound to Hb-exWAGO inside the nematode. This suggests potentially different secretion origins within the parasite and possibly different specific targets within the host. It is also possible that the two forms of Hb-exWAGO target similar host genes/pathways, and having both forms provides robustness to ensure sufficient Hb-exWAGO will find its way into the right host cells.

It remains unclear how the genomic loci that produce the exWAGO-bound sRNAs are selected to produce secreted sRNAs. Many of these loci contain non-autonomous LTR retrotransposons, which lack the coding regions necessary for transposition (preprint: Martínez-Ugalde et al, 2025). Further genomic analyses will shed light on the selective pressures driving their evolution. It is striking that LTR-derived sRNAs are also the key source of sRNAs involved in inter-species interactions between Oomycetes and plants, and these are reported as a new type of virulence factor (Porquier et al, 2021).

This work interfaces with an increasing number of reports suggesting extracellular Argonautes exist in multiple forms across plants and animals. In humans, these have been reported within EVs (Bukong et al, 2014; Mantel et al, 2016; McKenzie et al, 2016) as well as non-vesicular complexes including exomeres and supermeres (Jeppesen et al, 2019; Zhang et al, 2019, 2021), and other non-vesicular forms (Arroyo et al, 2011; Geekiyanage et al, 2020; Turchinovich et al, 2011). Given the ubiquity of extracellular Argonautes including in humans, our work points to a need of better understanding the targeting properties and function of both vesicular and non-vesicular forms of Argonautes across living systems.

## Methods

**Table:** Reagents and tools table

Reagent/resource	Reference or source	Identifier or catalogue number
**Experimental models**		
*Heligmosomoides bakeri*	Professor JM Behnke (University of Nottingham, UK)	
*Nippostrongylus brasiliensis*	Dr Bridget Ogilvie (MRC National Institute of Medical Research, London, UK)	
*Teladorsagia circumcincta*	Isolate derived from anthelmintic-susceptible UK *T. circumcincta* population and maintained by passage in sheep for >25 years (McIntyre et al, 2025)	MTci2
*Trichostrongylus colubriformis*	Isolate derived from anthelmintic-susceptible UK *T. colubriformis* population and maintained by passage in sheep for >20 years.	MTco1
*Ancylostoma ceylanicum*	Professor John Hawdon	
C57BL/6, female (*M. musculus*)	Bred in-house at a University of Edinburgh, UK-licensed establishment	
CBA x C57BL/6 F1, male (*M. musculus*)	Bred in-house at a University of Edinburgh, UK-licensed establishment	
Syrian hamsters, male (*Mesocricetus auratus*)	Envigo	HsdHan:AURA
Sprague–Dawley rats, male (*Rattus norvegicus*)	Harlan Olac (now Envigo)	Hsd:Sprague–Dawley SD
Sheep, mixed sex (*Ovis aries*)	Texel cross lambs, bred onsite at Moredun Research Institute, UK	
MODE-K cell line	Dr Dominique Kaiserlian	
RAW 264.7 cell line	ATCC	TIB-71
**Primary antibodies**		
Rabbit anti-Hb-exWAGO; polyclonal; used at 1:4000 in 5% BSA with 0.1% TBST for western blot analysis	Eurogentec, as described in Chow et al, 2019	
Mouse anti-Hb-exWAGO; clone C5F7; monoclonal; used at 1:2000 in 3% milk with 0.1% TBST for western blot analysis	This study; generated in-house as per Hewitson et al, 2011	
Rat anti-Hb-exWAGO antiserum; polyclonal; used at 1:300 for native exWAGO detection by ELISA, at 1:167 for blocking rexWAGO uptake, and for immunoprecipitations	Generated in-house; described in Chow et al, 2019	
Rat anti-Tc-exWAGO antiserum; polyclonal; used for immunoprecipitations	This study; generated in-house	
Rabbit anti-Hb-exWAGO UE3R2; polyclonal; used at 5.0 µg/ml for native exWAGO detection by ELISA; used at 2.5 µg/ml for immunohistochemistry	Sino Biological	
Rabbit IgG isotype control; polyclonal; used at 2.5 µg/ml for immunohistochemistry	Thermo Fisher Scientific	02-6102
Naive rat serum; polyclonal; used at 1:167 for blocking recombinant Hb-exWAGO uptake	This study; generated in-house	
Mouse anti-Hb-exWAGO antisera; polyclonal; used at 1:250 for blocking recombinant Hb-exWAGO uptake	This study	
Mouse anti-HES antisera; polyclonal; used at 1:250 for blocking recombinant Hb-exWAGO uptake	This study	
Mouse anti-PBS antisera; polyclonal; used at 1:250 for blocking recombinant Hb-exWAGO uptake	This study	
Mouse anti-mouse AGO2; monoclonal; used at 1:4000 in 3% milk with 0.1% TBST for western blot analysis	InVivo BioTech Services GmbH	DO-2 17F1-C1
Rabbit anti-calreticulin; used at 1:2000 in 3% milk with 0.1% TBST for western blot analysis	Cell Signaling Technology	2891S
Rabbit anti-GAPDH (14C10); monoclonal; used at 1:2000 in 3% milk with 0.1% TBST for western blot analysis	Cell Signaling Technology	2118S
Rabbit anti-Histone H3 (D1H2) XP®; monoclonal; used at 1:2000 in 3% milk with 0.1% TBST for western blot analysis	Cell Signaling Technology	4499S
**Secondary antibodies**		
Goat anti-rabbit IgG (H + L) Secondary Antibody, DyLight™ 800 4X PEG; used at 1:10,000 for western blot analysis	Thermo Fisher Scientific	SA5-35571
Goat anti-mouse IgG (H + L) Highly Cross-Adsorbed Secondary Antibody, Alexa Fluor™ 680; used at 1:10,000 for western blot analysis	Thermo Fisher Scientific	A21058
Goat anti-rabbit IgG-HRP-linked; used at 1:2000 for native exWAGO detection by ELISA	Cell Signaling	7074S
Goat anti-rabbit IgG (H + L) Highly Cross-Adsorbed Secondary Antibody, Alexa Fluor™ 647; used at 1:2000 for immunohistochemistry	Thermo Fisher Scientific	A21245
Goat anti-mouse IgG1, human ads-HRP; used at 1:2000 for IgG1 detection by ELISA	SouthernBiotech	1070-05
**Chemicals, enzymes and other reagents**		
Recombinant Hb-exWAGO protein	Sino Biological	
Recombinant Tc-exWAGO protein	Sino Biological	
Recombinant Proteinase K solution	Thermo Fisher Scientific	AM2546
Phenylmethanesulfonyl fluoride solution (PMSF)	Sigma Aldrich	93482-50 ml
ProLong™ Gold Antifade Mountant	Thermo Fisher Scientific	P36930
TMB 2-Component Microwell Peroxidase Substrate Kit	SeraCare	5120-0047
Cy5 NHS Ester dye	AAT Bioquest	151
Alexa Fluor™ 647 NHS Ester dye	Thermo Fisher Scientific	A37573
Ibidi mounting medium	Ibidi	50001
Imject™ Alum adjuvant	Thermo Fisher Scientific	77161
Dynabeads™ Protein G	Thermo Fisher Scientific	10003D
Stainless steel beads (5 mm)	Qiagen	69989
cOmplete™, mini protease inhibitor cocktail	Roche	4693124001
PhosSTOP™, phosphatase inhibitors	Roche	4906845001
RNasin® ribonuclease inhibitor, recombinant	Promega	N2515
RNA 5’ Polyphosphatase	Lucigen	RP8092H
CleanTag™ small RNA library preparation Kit	TriLink	L-3206
Bioanalyzer High Sensitivity DNA kit	Agilent	5067-4626
Precision Plus Protein™ all blue prestained protein standards	Bio-Rad	1610373
**Software**		
Image Studio Lite v5.2	LI-COR	
Image Lab v6.1.0 build 7	Bio-Rad	
ImageJ 1.52n	Fiji	
SkanIt software v2.4.5.9	Thermo Fisher Scientific	
Prism v10.1.2	GraphPad	
EVOS Analysis software v1.5.1479.304	Thermo Fisher Scientific	
Zen Blue v3.3, v3.5 and v3.6	Zeiss	
FlowJo software v10	BD	
Zetaview software v8.05.14SP7	Particle Metrix	
2100 Expert software vB.02.10.SI764	Agilent	
R v4.1.3, v4.5.0, v4.5.1	R Core Team, 2025	
FastQC v.0.12.1	https://www.bioinformatics.babraham.ac.uk/projects/fastqc/	
MultiQC v.1.14	Ewels et al, 2016	
Kraken	Davis et al, 2013	
Pullseq	Available at: https://github.com/bcthomas/pullseq	
ShortStack v.3.8	Johnson et al, 2016	
edgeR	Robinson et al, 2010	
miniprot	Li, 2023	
BLASTp	Altschul et al, 1990	
cutadapt v.4.9	Martin, 2011	
STAR v.2.7.10b	Dobin et al, 2013	
Rsubread	Liao et al, 2019	
DGEobj.utils	Thompson et al, 2022	
Biostrings	Pagès et al, 2025 https://bioconductor.org/packages/release/bioc/html/Biostrings.html	
pheatmap	Kolde, 2025 https://cran.r-project.org/web/packages/pheatmap/index.html	
ggseqlogo	Wagih, 2024 https://cran.r-project.org/web/packages/ggseqlogo/index.html	
TargetFinder	Fahlgren and Carrington, 2010	
Ensembl	Dyer et al, 2025	
Gencode	Mudge et al, 2025	
miRBase	Kozomara et al, 2019	
UCSC	Perez et al, 2025	
ggplot2	Wickham, 2016 https://ggplot2.tidyverse.org	
GenomicRanges v1.61.1	Lawrence et al, 2013	
**Other**		
5 kDa Vivaspin	Cytiva	28-9323-59
NuPAGE™ Bis-Tris mini protein gels, 4–12%, 1.0–1.5 mm	Thermo Fisher Scientific	NP0321BOX
Novex™ TBE gels, 6%	Thermo Fisher Scientific	EC6265BOX
Immobilon® -FL PVDF membrane	Millipore	IPFL00010
Zeba™ spin desalting columns, 7 K MWCO	Thermo Fisher Scientific	89882
SuperFrost Plus™ Adhesion slides	Epredia	J1800AMNZ
18-well ibidi-treat µslides	Ibidi	81816
McMaster Egg Counting Chamber	CellPath	RSW-0611240
Odyssey® CLx imaging system	LI-COR	
ChemiDoc MP imaging system	Bio-Rad	
Varioskan™ Flash	Thermo Fisher Scientific	
EVOS M7000 Imaging System	Thermo Fisher Scientific	
LSM 980 microscope with Airyscan 2 detector	Zeiss	
MACSQuant® Analyzer 10 flow cytometer	Miltenyi Biotec	
TissueLyser II	Qiagen	
Optima XPN-100 Ultracentrifuge	Beckman Coulter	
SW 40 Ti swinging-bucket rotor	Beckman Coulter	331301
14 mL Polypropylene Tube, 14 x 95 mm	Beckman Coulter	331374
Qubit® 3.0 Fluorometer	Thermo Fisher Scientific	
ZetaView® Twin Nanoparticle Tracking Analysis	Particle Metrix	
2100 Bioanalyzer	Agilent	
Illumina NextSeq 2000	Illumina	
Illumina NovaSeq 6000	Illumina	

### Ethics statement

All the mice used in this project were bred by the in-house facilities at The University of Edinburgh. Experimental procedures were executed under a UK Home Office licence P635073CF as approved by the University of Edinburgh Animal Welfare and Ethical Review Body (AWERB) and were carried out by qualified personnel in accordance with the UK Home Office guidelines. The experiments involving sheep were performed under the terms of a UK Home Office licence (PP5774688) using procedures ratified by the Moredun Research Institute AWERB and were carried out by suitably qualified personnel in accordance with the UK Home Office guidelines. All animal experiments involving rats were performed under UK Home Office licence and approved by the University of Glasgow AWERB. All housing and care of laboratory animals used in the hookworm study conform to the National Institutes of Health (NIH, USA). Guide for the Care and Use of Laboratory Animals in Research (see 18-F22) and all requirements and all regulations issued by the United States Department of Agriculture (U.S.D.A.), including regulations implementing the Animal Welfare Act (P.L. 89–544) as amended (see 18-F23).

All mice housed for experimental purposes were kept according to the UK Animals (Scientific Procedures) Act 1986, following the Home Office Code of Practice.

### Recombinant exWAGO proteins

The recombinant *H. bakeri* exWAGO (Hb-rexWAGO) and *T. circumcincta* exWAGO used in this publication were produced by Sino Biological in insect cells. The recombinant Hb-exWAGO is 107.3 kDa and is provided in PBS (pH 7.0), 10% glycerol, and 0.5 mM TCEP. The recombinant *T. circumcincta* exWAGO is 106.8 kDa and is provided in 20 mM PB, 300 mM NaCl, pH 7.0, 10% glycerol, 0.5 mM TCEP, and 0.5 mM PMSF. The recombinant proteins were designed to have 3x FLAG tags (underlined) and a PreScission cleavage site (underlined and bold), followed by a 6x His tag (underlined and italicised) at the N-terminus as shown below.

The recombinant Hb-exWAGO protein sequence is as follows: MDYKDDDDKDYKDDDDKDYKDDDDKAL**LEVLFQGP**ASG*HHHHHH*SGGGGSMDQLKTGMGQLSVGAVALPEKRSPGGIGNKVDFVTNLTELSLKPNVPYYKYDIRMYIVYKGNDALEHLKELTKQTKDDFPEQERKSAAVAVYKHLCKTYKDVFLPDGALLYDRAAVLFSAQRQLKLDGEEKQFMLPASVVSSAGPDATGIRVVIKKVKDQFQVTSNDLSKAVNVRDMERDKGILEVLNLAVSQKGYMETSQFVTYGSGVHYLFDHRALGFRDNELPELMDGKYMGIGLTKSVKVLEGDSGKGNSAFVVTDVTKGAFHVDEQNLMEKISQMSIFFDQRTGQSSFNAKNAMQPFNQKAILQQIKGLYVRTTYGKKKTFPIGNLAAAANALKFQTADGAQCTVEQYFKKHYNIQLKYPGMFTVSERHNPHTYYPVELLTVAPSQRVTLQQQTPDQVASMIKASATLPQTRLHQTKIMKDALDITPRNHNLATAGISVANGFTAVSGRVLPSPRIAYGGNQILRPVDNCKWNGDRSVFLEPAKLTNWAVCVTLTQQDARRLQIKEYISRVEMRCRNRGMQVDPVAEVFTLKHQTFDGLKEWYASQKQKNRRYLMFITSDGIKQHDSIKLLEVEYQIVSQEIKGSKVDAVVTKNQNQTLDNVVAKINMKLGGVNYNVMLGVKNDDKAFSWLNDKDRMFVGFEISNPPALSKVEIERGASYKMPSVLGWGANCAGNHQQYIGDYVYIQPRQSDMMGAKLSELIVDILKRFRAATTIAPRHIVLYFSGISEGQFSLVTDTYMRAVNTGIASLSPNYKPSVTAVAVSKDHNERIYKTNISGNRATEQNIPPGTVIDTKIVSPVINEFYLNSHSAFQGTAKTPKYSLLADNSKIPLDVIEGMTHGLCYLHEIVTSTVSVPVPLIVADRCAKRGHNVYIANSNQGEHSVNTIDEANAKLVNDGDLKKVRYNA.

The recombinant *T. circumcincta* exWAGO protein sequence is as follows: MDYKDDDDKDYKDDDDKDYKDDDDKAL**LEVLFQGP**ASG*HHHHHH*SGGGGSMADQLSGGMGKLSVAAVALPEKRAPGSLGTKLDFVTNLTGIKLKPNVPYYKYDVRMYIVYKGNDGREVLKELTKQTKDDFPEQERKMAAVAIYKHLVKSYKDIFPQDGQFFYDRAAVLFSAQREMKLGGPEKVITLPASLSPTAGSDAAGIRVVIKKVTDGYQVTSNDLMKAVNVRDCERDKGILEVLNLAVSQKGYMETSQFVTYGTGVHYLYDHRALGFRDNELPDLMDGKYMGIGLTKAVKVLEGDQGKSASAFVVTDVTKGAFHIDEQNLLEKISQMSIFFDPRTGQSTFSVKAAMQPHNMKSILQLIKGLYVRTTYGRKRTFPIGNLAAAPNALKLQTSDGVQCTIEQYFKKQYNVQLKYPGLFTVSERHNPHNYYPVELLTVAPSQRVTLQQQTPDQVASMIKASATLPSNRLHQTKVMKEALDITPRNAKLASAGINVEDGFTTVPGRVLPTPTILYGGSQTLKPVDNCKWNGDRSRFLEPAQLTNWAVCATLTQNDARRLQIKDYVARVESRCRAKGMQVEAAAEIFTLTKQNFDGLREFYAAQKKKNRKYLLFITSDGIKQHDLIKLLEVEYQIVSQEVKGSKVDSVMFKNQNQTLDNVIAKINMKLGGVNYNVVLGSKPNDPASKWLNDKDRLFVGFEISNPPALSKMEIERGATYKMPSVLGWGANCAANPQHYIGDYVYIKPRQSDMMGAKLSELIVEILKKFRGATSLAPRHIVLYFSGISEGQFSLVTDTYMKAINTGITSLSANYRPSVTALAVSKDHNERLYKSNISGSRANEQNIPPGSVVDTKIVSPVINEFYLNSHSAFQGTAKTPKYSLLADDSKIPLDVIEGMTHGLCYLHEIVTSTVSVPVPLIVADRCAKRGHNIFIANSNLGSAAVSSIEEANEKLVNHGELEKVRYNA.

### Parasite life cycle and collection of excretory/secretory products *H. bakeri*

For maintaining the life cycle of *H. bakeri*, CBA × C57BL/6 F1 male mice were infected with 400 *H. bakeri* L3 stage larvae by oral gavage and adult worms were harvested from the small intestine 14 days post-challenge. Adult worms were extensively washed and cultivated in serum-free media (RPMI 1640 supplemented with 1.2% glucose, 5 U/ml penicillin, 5 µg/ml streptomycin, 2 mM L-glutamine and 1% gentamycin) as described in Johnston et al (2015). For the collection of the excretory/secretory products from *H. bakeri* (HES), conditioned media were collected on days 4 and 8 post-culture; however, the first 24 h of culture media was first removed to reduce any potential host contamination. The HES collected was spun (300 rcf, 5 min, room temperature) to remove eggs, filtered (0.22-μm Millex-GP filter), and stored at −20 °C until required. HES (~50–60 ml per batch of HES; batch is defined as the parasites collected and pooled from one group of infected mice) was concentrated to <12.5 ml using a 5 kDa Vivaspin (3000 rcf, 4 °C) (Cytiva). Concentrated HES was then subjected to ultracentrifugation (100,000 rcf, 70 min, 4 °C) in polyallomer centrifuge tubes (Beckman Coulter) in a SW 40 Ti swinging-bucket rotor (Beckman Coulter). The supernatant (SUP, also known as “EV-depleted HES”) was collected and concentrated to a final volume of 0.4–1.0 ml after buffer exchange in >40 ml of PBS using a 5 kDa Vivaspin (3000 rcf, 4 °C) (GE Healthcare). The EV pellet was washed twice with 12.5 ml of cold PBS and spun as before. PBS washes were discarded, and the EV pellet was resuspended in 120–150 μl PBS. The protein concentration of EVs and EV-depleted HES was measured using the Qubit Protein Assay kit (Thermo Fisher Scientific), and the material was stored at −80 °C until required. The number of particles in the purified EVs was measured using the ZetaView Nanoparticle Tracking Analysis (Particle Metrix) as per the manufacturer’s instructions. This study adheres to MISEV2023 guidelines (Welsh et al, 2024) and guidelines for helminth-derived EVs as per White et al, (2023).

### *N. brasiliensis*

The *N. brasiliensis* life cycle was maintained by infection of Sprague–Dawley male rats with 3000 infective L3 larvae. Adult worms were obtained 8 days later from small intestinal tissues in a Baermann apparatus as described in Lawrence et al (1996). Worms were washed extensively in PBS, and stored at −80 °C until required.

### *A. ceylanicum*

*A. ceylanicum* hookworm life cycle was maintained in hamsters (Hu et al, 2012, 2018). Adult worms were collected from the gut of hamsters at day 20 post-infection. Following extensive washes with Hank’s Balanced Salt Solution, worms were frozen at −80 °C until used.

### *T. circumcincta* and *T. colubriformis*

For maintaining the life cycle of *T. circumcincta and T. colubriformis*, helminth-free lambs (< 6 months old) were orally infected with L3 stage parasites (McIntyre et al, 2025). For the generation of L4 and adult parasites animals were orally challenged with 150,000 or 20,000 L3 stage larvae, respectively. Parasites at L4 stage (7 days post-infection) and adult stage (28 days post-infection) were harvested from either the sheep gastric stomach (for *T. circumcincta*) or the first 3 m of the small intestine (for *T. colubriformis*). Worms were washed three times in PBS and then cultured in RPMI 1640 medium (Thermo Fisher Scientific) containing 20 mM HEPES pH 7.5, 1% (*w*/*v*) d-glucose, 2 mM L-glutamine, 1000 U/ml penicillin, 1000 μg/ml streptomycin, 200 μg/ml gentamycin and 25 μg/ml amphotericin B, at 37 °C in 5% CO_2_. Culture-conditioned media were harvested every 24 h and replaced with fresh media, with worms maintained in culture for 120 h post collection. At each supernatant collection, parasite viability was confirmed on the basis of structural integrity and motility.

Following collection, the culture supernatants were clarified by centrifugation at 300 rcf for 10 min at 4 °C, filtered through a 0.2-μm Minisart syringe filter (Sartorius) and stored at −70 °C. For processing of excretory-secretory (ES) products, culture supernatants from all collections were pooled, then concentrated and buffer exchanged into PBS using 10-kDa MWCO Amicon Ultra-15 Centrifugal Filter Units (Millipore Sigma), following the manufacturer’s guidelines. Protein concentration was determined using the Pierce™ BCA protein assay (Thermo Fisher Scientific) with bovine serum albumin standards, and aliquots of ES products were stored at −70 °C prior to use.

### Cell culture

The immortalised intestinal epithelial MODE-K cell line (derived from the small intestine of C3H/He female mice) was kindly provided by Dominique Kaiserlian (French Institute of Health and Medical Research) and were cultured at 37 °C with 5% CO_2_ in DMEM medium supplemented with foetal bovine serum (10%), L-glutamine (1%), penicillin/streptomycin (1%), sodium pyruvate (1%) and non-essential amino acids (1%) (Vidal et al, 1993). The murine macrophage RAW 264.7 cell line (ATCC) was cultured at 37 °C with 5% CO_2_ in DMEM medium supplemented with foetal bovine serum (10%), L-glutamine (1%), and penicillin/streptomycin (1%). The cell lines have been regularly tested for mycoplasma contamination.

### Proteinase K treatment

*H. bakeri* EVs and EV-depleted HES (2.0 μg of protein) were treated with or without Triton X-100 treatment (0.05%, 30 min, on ice), followed by Proteinase K digestion (5 μg/ml, 30 min, 37 °C) (Epicentre) as appropriate. The reaction was quenched by addition of phenylmethanesulfonyl fluoride (5 mM, 1 h, on ice) (Sigma). Samples were then prepared for western blot analysis.

### Western blot analysis

Protein samples were reduced and separated on 4–12% Bis-Tris NuPAGE SDS gels (Thermo Fisher Scientific). The separated proteins were transferred to an Immobilon-FL PVDF membrane (Millipore) by wet transfer (100 V, 105 min). The membrane was blocked and then incubated with the primary antibody (4 °C, overnight, on roller). For detection of Hb-exWAGO protein, a polyclonal rabbit antibody generated and purified against the peptide TKQTKDDFPEQERK (Eurogentec) was used at 1:4000 in 5% BSA/0.1% TBST (Chow et al, 2019), unless otherwise specified. For the detection of exWAGO orthologues in ES from *T. circumcincta* and *T. colubriformis*, a mouse monoclonal antibody (clone C5F7) generated in-house as described in Hewitson et al (2011) against recombinant Hb-exWAGO was used at 1:2000 in 3% milk/0.1% TBST. Following incubation with primary antibodies, the membrane was incubated (1 h, room temperature) with goat anti-rabbit IgG DyLight 800 (Thermo Fisher Scientific, SA5-35571) or goat anti-mouse IgG AF680 secondary antibodies (Thermo Fisher Scientific, A21058). The signal was visualised using the Odyssey CLx imaging system (LICOR) and band intensities were quantified using ImageJ (Fiji) as described in Stael et al (2022).

### ELISA for the detection and quantification of native Hb-exWAGO protein

Secreted Hb-exWAGO from EVs, EV-depleted HES and total HES were measured using ELISA. Immuno plates (Thermo Fisher Scientific) were coated with polyclonal rat anti-Hb-exWAGO antiserum capture antibody (raised against recombinant Hb-exWAGO in-house) in 0.06 M carbonate buffer (4 °C, overnight) and blocked (4% BSA/TBS, 37 °C, 2 h). Samples were lysed as required with 0.05% Triton X-100 (30 min, on ice), and plated (4 °C, overnight) in dilution buffer (1% BSA/TBST). For detection, the polyclonal rabbit UE3R2 anti-exWAGO primary antibody (produced by Sino Biological against recombinant Hb-exWAGO) was used (5 μg/ml in dilution buffer, 2 h, room temperature) followed by incubation with goat anti-rabbit IgG-HRP (Cell Signalling, 7024S) secondary antibody (1:2000 dilution, 37 °C, 1 h, in the dark). The reaction was developed using TMB substrate buffer (SeraCare), stopped (10% phosphoric acid), and the optical density was read at 450 nm on the Varioskan Flash (Thermo Fisher Scientific). Samples were analysed in technical duplicates, and wells with buffer only (no sample) were used to determine the background signal.

For quantification of Hb-exWAGO, a standard curve with recombinant Hb-exWAGO with 0.05% Triton X-100 in dilution buffer was prepared in a fourfold serial dilution (from 0.01907 pg to 20 ng) and analysed using a 4PL regression (GraphPad Prism). The amount of Hb-exWAGO was interpolated using the optical density values obtained in the ELISA assay. The limit of detection was calculated from 5 biologically independent experiments (each containing at least two technical replicates) as the mean of blanks + (standard deviation of blanks * 3.3) and the limit of quantification was calculated as the mean of blanks + (standard deviation of blanks * 10).

For the detection of Hb-exWAGO secreted at different *H. bakeri* life stages, female C57BL/6 mice were infected with 200 L3 stage larvae by oral gavage. At day 5, 7 and 14 post-challenge mice were culled, and larvae or adult worms were collected from the gut wall or lumen of the small intestine, respectively. Larvae were washed 5× with Hanks’ Balanced Salt Solution supplemented with penicillin–streptomycin (1%). Adult worms were treated with gentamycin (1 mg/ml) in Hanks’ Balanced Salt Solution supplemented with penicillin and streptomycin (1%). Worms were then visually inspected for motility and any obvious injuries and were cultivated in groups of 20 (3 biological replicates per group) in a 48-well plate with 500 µl serum-free culture media. The first 24 h of culture media was removed and replenished. HES was collected and replenished 2 and 4 days after culture. For the ELISA assay, day 2 and 4 HES were pooled by equal volume and tested directly (i.e., no PBS exchange), while culture media-only samples were used as a negative control. Samples were tested in technical quadruplicates, and a biological replicate from L4 stage larvae (d5 p.c.) was excluded from analysis due to large variation between the technical replicates performed.

For quantification of Hb-exWAGO secreted by adult worms harvested at day 14 post infection: Worms were cultivated in groups of 30 (15 male and 15 female as determined by visual inspection) in a 24-well plate with 400 µl serum-free culture media. The first 24 h of culture media was removed, replenished, and HES was collected 4 days post culture. The HES collected was tested directly (i.e., no PBS exchange) by ELISA, and culture media-only samples were used as negative controls. Samples were analysed in technical duplicates.

### Uptake of non-vesicular proteins

To examine if non-vesicular proteins are internalised by epithelial cells in vitro, proteins in EV-depleted HES or PBS were labelled using a Cy5 NHS Ester dye (AAT Bioquest, 151) according to the manufacturer’s instructions at a 15:1 (dye to protein) molar ratio. Unconjugated dye was removed using the Zeba 7 K MWCO spin columns (Thermo Fisher Scientific, 89882) as per the manufacturer’s instructions. The labelled EV-depleted HES and PBS were stored (−80 °C, dark) until required. MODE-K cells (1.5 × 10^5^) were seeded in a 24-well plate with 400 μl MODE-K media overnight. The cells were treated with Cy5-labelled EV-depleted HES at a final concentration of 10 μg/ml or 50 μg/ml or with an equivalent volume of Cy5-labelled PBS as a negative control for 4 or 24 h. The cells were washed twice with PBS, supplemented with fresh media, and imaged on the EVOS M7000 Imaging system (Invitrogen) with the EVOS LED Light Cube Cy5 2.0 using the EVOS 60x objective, fluorite, LWD. Data were analysed on the EVOS Analysis software v1.5.1479.304.

### Immunohistochemistry (IHC)

To detect Hb-exWAGO in vivo, C57BL/6 female mice were infected with 200 L3 stage larvae by oral gavage. At day 7 of infection, mice were culled, and the upper half of the small intestine was removed for preparation into a gut roll. Fat was trimmed away from the gut wall, and the intestines were rolled inside-out onto wooden skewers. The intestines were fixed for 30 min in 4% PFA (diluted from 16% methanol-free PFA, VWR). The intestines were cut lengthwise and rolled onto a new skewer as ‘gut rolls’, then incubated for a further 5 h in fresh 4% PFA. The gut rolls were washed twice in PBS and then stored overnight in 70% ethanol before processing sequentially in 80% ethanol (30 min), 95% ethanol (30 min, twice), 100% ethanol (30 min, twice), xylene (30 min, twice), and paraffin wax (40 min at 60 °C), before embedding in paraffin wax. 10 µm sections were cut from the resulting blocks, which were mounted on Superfrost Plus slides (VWR), dried overnight, baked for one hour at 60 °C and stored at 4 °C. Wax was removed for staining by washing sequentially in xylene (3 min, twice), xylene/ethanol mixed 1:1 (3 min), 100% ethanol (3 min, twice), 95% ethanol (3 min), 70% ethanol (3 min), 50% ethanol (3 min), before a final rinse with water. Antigen retrieval involved a 30-min treatment in citrate buffer (Abcam) in a 98 °C water bath, before a further 20-min cool-down outside the water bath. Slides were washed in TBS and then blocked using TBS buffer with 1% BSA and 0.1% Tween-20. For Hb-exWAGO detection, 2.5 µg/mL of rabbit anti-Hb-exWAGO antibody UE3R2 or the polyclonal rabbit IgG isotype-matched control antibody (Thermo Fisher Scientific, 02-6102) were incubated on the slides overnight in TBS buffer with 0.1% Tween-20 at 4 °C. After washing three times in TBS buffer with 0.1% Tween-20, Alexa Fluor 647 anti-rabbit secondary antibody (diluted 1:2000 in TBS with 0.1% Tween-20) (Thermo Fisher Scientific, A21245) was incubated on the slides for two hours at room temperature. DAPI diluted 1:1000 was added to the slides and washed off twice in TBS before mounting in ProLong Gold (Thermo Fisher Scientific). The slides were imaged on a Zeiss LSM 980 microscope with Airyscan 2 detector, using the Zeiss Plan Apochromat 20×0.8 NA Air M27 objective, with 405 and 639 nm lasers. AF647 signal was excited at 653 nm and detected at 668 nm. DAPI staining was excited at 353 nm and detected at 465 nm. Data were acquired using the Zen Blue 3.5 (Zeiss) and processed using the Airyscan processing setting SuperResolution 4.7 (2D, SR, Manual) with the Zen Blue 3.3 software (Zeiss). For data shown in Fig. 3A, images were acquired using 1.8 scan zoom and after processing they are 16 bit depth with 7553 × 3867 pixels (for the Infected mouse with anti-Hb-exWAGO staining) or 2024 × 2024 pixels (for the Infected mouse with anti-IgG and Naive mouse control with anti-Hb-exWAGO staining).

The number of Hb-exWAGO+ cells was counted using QuPath (Bankhead et al, 2017). Initially, all the images were loaded, and the nuclei were segmented based on DAPI staining using the Cellpose model “cyto3” (Stringer and Pachitariu, 2025) with the following parameters pixel size: 0.15, flow threshold = 0.6 and cell median diameter = 35 on a full image annotation. An object classifier was then created and trained using 4 random images for detecting Hb-exWAGO using the RandomTrees Classifier to detect positive cells for Hb-exWAGO. Annotations were made around the parasite and the distance between each Hb-exWAGO classified cell and the parasite was calculated using the QuPath function “Distance to annotation”. The raw counts were then plotted using ggplot2 ‘s geom_density function (Wickham, 2016) in RStudio (v4.5.0) (R Core Team, 2025).

### Uptake and blockade of recombinant Hb-exWAGO

To test whether non-vesicular Hb-exWAGO is internalised by epithelial and macrophage cells in vitro, equimolar amounts of recombinant Hb-exWAGO, BSA and PBS were labelled with AF647 NHS Ester dye (Thermo Fisher Scientific, A37573) according to the manufacturer’s instructions at a 15:1 (dye to protein) molar ratio. Unconjugated dye was removed using the Zeba 7 K MWCO spin columns (Thermo Fisher Scientific, 89882) as per the manufacturer’s instructions. The labelled proteins and PBS were stored (-80 °C, dark) until required.

To assess the uptake of AF647-labelled Hb-exWAGO by mouse cells using confocal microscopy, 1.0 × 10^4^ MODE-K or RAW 264.7 cells were seeded in an 18-well ibidi-treat µ-slide (Ibidi, 81816) in 100 µl culture media overnight. The cells were incubated (37 °C with 5% CO_2_) with 0.1 µM Hb-exWAGO, BSA, or PBS labelled with AF647 NHS Ester for 4 h. For blocking the uptake of Hb-exWAGO in MODE-K cells, anti-Hb-exWAGO rat serum (produced in-house against recombinant Hb-exWAGO as described in Chow et al (2019)) or naïve rat serum were added at a 1:150 final dilution mixed with the labelled protein. Following incubation, the cells were washed twice with PBS, fixed with 4% PFA (10 min), washed again, stained with DAPI, washed again, and mounted using the ibidi mount medium (Ibidi, 50001). Slides were stored (4 °C, dark) and imaged within three days of preparation. The samples were illuminated with transmission light, the 405 and 639 nm lasers and imaged on the Zeiss laser scanning confocal microscope LSM 980 with Airyscan 2 detector using the Zeiss 63×1.4 NA Oil DIC M27 objective. AF647 signal was excited at 653 nm and detected at 668 nm. DAPI staining was excited at 353 nm and detected at 465 nm. Transmitted light was captured on confocal mode using the T-PMT transmission detection module. Data were acquired using 2.1 scan zoom (Figs. 3B and 4F) using the Zen Blue 3.5 (Zeiss) and processed using the airyscan processing setting SuperResolution (2D, Auto) the Zen Blue 3.3 and 3.6 software (Zeiss). Following processing, the images are 16-bit depth and 1793 × 1793 pixels.

To assess the uptake and blocking of AF647-labelled recombinant Hb-exWAGO by mouse cells using flow cytometry, 0.8 × 10^5^ MODE-K cells were seeded in a 24-well plate in 500 µl culture media overnight. The cells were incubated (37 °C with 5% CO_2_) with 10 nM of AF674-labelled Hb-exWAGO, BSA, or PBS for 4 h. For blocking the uptake of recombinant Hb-exWAGO in MODE-K cells, sera obtained from Hb-exWAGO-, HES-, or PBS-vaccinated mice were added to the cells at a 1:250 final dilution directly prior to the addition of AF647-Hb-exWAGO. Following incubation, the cells were washed twice with PBS, trypsinised (5 min, at 37 °C with 5% CO_2_), resuspended in 5% FBS/PBS and centrifuged (400 rcf, 5 min, room temperature). The cells were resuspended in 0.5% FBS/PBS and analysed on the MACSQuant Analyser 10 (Miltenyi Biotec) using the R1 channel. Data were analysed using FlowJo software v10.

### Nuclear fractionation

To interrogate the localisation of internalised exWAGO, we employed nuclear fractionation using an adapted protocol from Burke and Sullivan (2017). MODE-K cells (0.2 × 10^6^ cells/ml) were seeded in 1 ml of media in a 12-well plate overnight and treated with 0.1 μM of AF647-labelled recombinant Hb-exWAGO or PBS for 4 h. The cells were washed twice with PBS, harvested, and further washed with PBS. The cells were then lysed in 50 μl of CSKT buffer (10 mM PIPES pH 6.8, 100 mM NaCl, 300 mM sucrose, 3 mM MgCl_2_, 1 mM EDTA, 1 mM DTT, 0.5% Triton X-100) containing protease inhibitors (Roche; 1 tablet per 10 ml) on ice for 10 min. The nuclei were pelleted (5,000 rcf, 5 min, 4 °C), and the supernatant containing the cytoplasmic fraction was collected and mixed with an equal volume of SDS lysis buffer (1% SDS, 2% β-mercaptoethanol). The pelleted nuclei were washed once with 100 μl of CSKT buffer, resuspended in 50 μl of water and lysed in 50 μl of SDS lysis buffer. All samples were boiled (95 °C, 10 min) and vortexed (30 s). Equal volumes of whole-cell lysate, cytoplasmic and nuclear fractions were analysed using western blot analysis.

### Immunisation of mice

For vaccine experiments, female C57BL/6 mice between 7 and 12 weeks old were immunised intraperitoneally with recombinant Hb-exWAGO or PBS or HES in Imject alum adjuvant (Thermo Fisher Scientific) at 1:1 volume ratio (total intraperitoneal injection volume was 200 μl). Mice were primed (10 μg) and boosted (2 μg) twice at 28 and 35 days post priming as described in Hewitson et al (2015). Seven days following the second boost, the mice were challenged with 200 *H. bakeri* L3 stage larvae by oral gavage. Mice were culled 14 and 28 days post-challenge by CO_2_ overdose or by intraperitoneal administration with Dolethal (30 mg, Vetoquinol). We note that a prolonged period of 3 months between priming and the first boost occurred for one of the three biologically independent experiments due to COVID-19 interruptions. No randomisation or blinding was used, and no animals were excluded from analysis.

The small and large intestines were carefully removed, stretched, and the number of adult worms counted. The number of eggs was measured using the McMaster Egg Counting Chamber (CellPath) following floatation with saturated sodium chloride solution as described in Camberis et al (2003). The blood serum was acquired via tail bleed or for euthanised mice it was collected using cardiac puncture. The blood was incubated (1–2 h, room temperature) and centrifuged (5000 rcf, 5 min, room temperature). The supernatant containing the serum was centrifuged again as before and stored at -80 °C until required.

### ELISA for measuring Hb-exWAGO-specific antibody responses

Hb-exWAGO-specific serum antibody responses were measured using ELISA. Plates were coated with 0.05 μg of recombinant Hb-exWAGO (Sino Biological) in 0.06 M carbonate buffer (4 °C, overnight), blocked (4% BSA/TBS, 37 °C, 2 h), and blood serum was plated in a twofold serial dilution (4 °C, overnight) in dilution buffer (1% BSA/TBST). Hb-exWAGO-specific IgG1 antibodies were then detected using the goat anti-mouse IgG1-HRP (SouthernBiotech, 1070-05) (1:2000 dilution, 37 °C, 1 h, in the dark). The reaction was developed using TMB substrate buffer (SeraCare), stopped (10% phosphoric acid), and the optical density was read at 450 nm on the Varioskan Flash (Thermo Fisher Scientific). Samples were analysed in technical duplicates, and wells without a sample were used to determine the background signal.

### exWAGO immunoprecipitation

exWAGO was immunopurified using polyclonal rat anti-exWAGO serum (generated in-house against the *H. bakeri* or *T. circumcincta* recombinant exWAGO protein) or naive rat serum antibodies conjugated to Protein G magnetic beads (Thermo Fisher Scientific, 10003D). In all immunoprecipitations, *H. bakeri* anti-exWAGO rat serum was used except for pull-downs from *T. circumcincta* worms, where *T. circumcincta* anti-exWAGO rat serum was used as this was available. Beads were washed five times in cold Binding Wash Buffer (PBS, 0.02% Tween-20), and the antibodies were then conjugated on the washed beads by incubation (2 h, 4 °C, rotating wheel). Unconjugated antibody was then removed, and the beads were equilibrated with the appropriate lysis buffer three times.

To immunoprecipitate exWAGO and its orthologues from adult worms, nematodes were lysed by bead beating (5 mm steel bead, Qiagen) using the Tissue Lyser II (Qiagen) in pre-cooled cartridges for 2 min at 30 Hz twice in worm lysis buffer (150 mM NaCl, 10 mM Tris.HCl, 0.5 mM EDTA, 0.5% NP40) containing protease and phosphatase inhibitors (1 tablet per 5 ml, Roche), and 200 U/ml RNase inhibitors (Promega). Unlysed material was removed by centrifugation (16,100 rcf, 10 min, 4 °C). The supernatant containing lysed worms was quantified using the Qubit Protein Assay kit (Thermo Fisher Scientific). Per immunoprecipitation, 150 μg of adult worm lysate protein was used.

To identify the small RNAs associated with the vesicular and non-vesicular Hb-exWAGO, we immunoprecipitated Hb-exWAGO from EVs (17 μg of protein) and EV-depleted HES (170 μg of protein) derived from corresponding batches. We used 10X more EV-depleted HES by protein weight to account for lower amounts of exWAGO recovered in immunoprecipitations from EV-depleted HES compared to EVs. EVs or EV-depleted HES were lysed (20 min, on ice) in TBS/0.05% Triton X-100 with protease inhibitors (1 tablet per 10 ml, Roche) and 200 U/ml RNase inhibitors (Promega).

The lysate was incubated with the antibody-conjugated beads (45 min, 4 °C, rotating wheel). The beads were washed with cold Low Salt buffer (50 mM Tris.HCl pH 7.5, 300 mM NaCl, 5 mM MgCl_2_, 0.5% Triton X-100 and 2.5% glycerol), followed by two washes with cold High Salt buffer (50 mM Tris.HCl pH 7.5, 800 mM NaCl, 10 mM MgCl_2_, 0.5% Triton X-100 and 2.5% glycerol) for 5 min at 4 °C, using a rotating wheel. The beads were washed once more with Low Salt buffer, followed by a cold PNK buffer wash (50 mM Tris.HCl pH 7.5, 50 mM NaCl, 10 mM MgCl_2_, 0.5% Triton X-100). For western blot analysis proteins were eluted in denaturing buffer, whereas for small RNA sequencing analysis the RNA was eluted directly in Qiazol (700 μl, 5 min, room temperature) (Qiagen).

### Small RNA libraries

RNA in Qiazol (Qiagen) was spiked with 7 μl of 10 pM RT4 synthetic spike (CUUGCGCAGAUAGUCGACACGA) and extracted using the miRNA Serum/Plasma kit (Qiagen) according to the manufacturer’s instructions. The purified RNA was treated with RNA 5’ Polyphosphatase (Lucigen) according to the manufacturer’s instructions. The reaction was terminated by ethanol precipitation (−70 °C, overnight), and the precipitated RNA was resuspended in 2.5 μl of nuclease-free water and 2.5 μl of Buffer 1 (TriLink, L-3206). The small RNA libraries were prepared using the CleanTag Small RNA Library Preparation Kit (TriLink, L-3206) according to the manufacturer’s instructions using half reaction volumes. All libraries were generated using 1:12 dilution of 5’ and 3’ adaptors with 20 amplification cycles. The profile of each small RNA library prior to sequencing was assessed using either Novex 6% TBE acrylamide gels (Thermo Fisher Scientific) or the High Sensitivity DNA Bioanalyser chip (Agilent). Pooled libraries were size selected (140–220 bp for libraries made from adult worms or 140–180 bp for EV and EV-depleted HES libraries) using gel purification to remove adaptor dimers. The libraries were sequenced on the Illumina NextSeq 2000 platform by the Edinburgh Clinical Research Facility or on the Illumina NovaSeq 6000 by Edinburgh Genomics using single-end 100 base pair reads.

### Small RNA bioinformatic analysis

The raw sequencing data were checked using FastQC v.0.12.1 (https://www.bioinformatics.babraham.ac.uk/projects/fastqc/) and MultiQC v.1.14 (Ewels et al, 2016) with no issues identified. The 3’ adapter sequence (TGGAATTCTCGGGTGCCAAGG) was removed using the Reaper tool from the Kraken package (Davis et al, 2013), and 18-27 nt reads were retrieved using Pullseq (available at: https://github.com/bcthomas/pullseq). ShortStack v.3.8 (Johnson et al, 2016) was then used to align reads to their respective genome, allowing up to one mismatch and weighted assignment of multi-mapping reads (--nohp --mincov 5 --pad 1 --mismatches 1 --mmap u --bowtie_m all --ranmax ‘none’). Count matrices were prepared across all samples, as well as plots showing the length distribution and first nucleotide preference, using the 01.featureCounts.R, 02.get_fn_mtx.R and 03.fn_long_cpm.R R scripts (available at: https://github.com/imu93/exwago_vaccine). Count values were transformed to counts per million (CPM) using the cpm function from the edgeR R package (Robinson et al, 2010). The data are deposited in the NCBI Sequence Read Archive (SRA) under the Bioproject ID PRJNA1200757.

### Differential expression analysis of Hb-exWAGO immunoprecipitation from EVs and EV-depleted HES

Count matrices were first filtered to remove regions with less than five CPMs using the filterByExpr function from edgeR (Robinson et al, 2010). Prior to comparing the sRNA populations bound by Hb-exWAGO in EVs and EV-depleted HES, we performed individual differential expression analyses by comparing each IP against the unbound fraction of the same sample. Normalisation factors were estimated using the expression levels of sense-stranded rRNA fragments. These were then used to fit negative binomial generalised log-linear models with the glmFit function from edgeR (Robinson et al, 2010). The glmTreat test was applied to identify regions significantly enriched in each secreted Hb-exWAGO form (fold-change threshold = 2). Next, we used the union of differentially expressed regions from the Hb-exWAGO comparisons to perform a second differential expression analysis between EV- and EV-depleted HES-enriched regions. Prior to fitting negative binomial generalised log-linear models, we performed a multidimensional scaling analysis using all IP-enriched regions with the plotMDS function from edgeR (Robinson et al, 2010) and default parameters, to assess similarities between samples. For differential expression analysis, normalisation factors were estimated using Trimmed Mean of M-values (TMM) normalisation, followed by glmFit and glmTreat (fold-change threshold = 2) to identify regions enriched in each Hb-exWAGO form (FDR 1%). The top 25 regions (by logCPM) were selected for each exWAGO form, and the most abundant sRNA (by logCPM) was selected from each region. Nucleotide frequencies were calculated using the alphabetFrequency function from Biostrings (Pagès et al, 2025). Heatmaps were plotted in R (R Core Team, 2025) using the pheatmap package (Kolde, 2025) and sequence logos with ggseqlogo (Wagih, 2024).

### Target predictions against the mouse genome

To predict targets to the mouse genome (GRCm39 Ensembl release 114) (Dyer et al, 2025) for exWAGO-bound sRNAs, we used TargetFinder (Fahlgren and Carrington, 2010), allowing a maximum penalty score of 3. To avoid bias due to length differences between EV and EV-depleted HES Hb-exWAGO sRNAs, we only used the first 20 nt of each sRNA. To query each predicted target site, we took the GTF annotation from Ensembl (Dyer et al, 2025), added tRNAs from Gencode (Mudge et al, 2025), miRNAs from miRBase (Kozomara et al, 2019) and repeats from UCSC (Perez et al, 2025). Introns were identified in protein-coding and lncRNA genes using an ad hoc script in R (R Core Team, 2025) with functions from GenomicRanges 1.61.1 (Lawrence et al, 2013). We then removed overlaps with the following preference: rRNA > miRNA > tRNA > Other ncRNA > protein-coding exons > lncRNA exons > repeats > introns > any remaining annotation. “Other ncRNA” = misc_RNA, scaRNA, scRNA, snoRNA, snRNA, sRNA. This allowed us to assign a single annotation to each target site. This non-overlapping annotation was also used to calculate the percentage of the mouse genome covered by each category.

### Identification of exWAGO orthologues and protein sequence alignment

Given the release of new genome assemblies for Strongylida parasites since the curation of exWAGO annotations (Chow et al, 2019), gene and protein exWAGO annotations of each genome were updated using miniprot (Li, 2023). Where available, the curated exWAGO protein was used. In cases where no curated exWAGO for that species was available, the sequence of the most closely related species was used for gene and protein prediction (e.g. *T. colubriformis* and *T. circumcincta*). Amino acid identity was further calculated using BLASTp (Altschul et al, 1990) against Hb-exWAGO annotated in the nHp.2.0 assembly (Chow et al, 2019), as shown in Dataset EV2 (Data ref: NIH Bioproject PRJEB15396, 2016; Herzog et al, (2024); Data ref: Herzog et al, (2024); Schwarz et al, 2015; Data ref: Schwarz et al, 2015;  Xu et al, (2019); Data ref: Xu et al, (2019); Data ref: NIH Bioproject PRJEB494, 2018; Data ref: NIH Bioproject PRJEB76626, 2024; Laing et al, (2013); Data ref: Laing et al, 2013; Data ref: NIH Bioproject PRJNA1007425, 2023; Data ref: NIH Bioproject PRJNA994163, 2023; Data ref: NIH Bioproject PRJNA72579, 2014; McIntyre et al (2025); Data ref: McIntyre et al, 2025; Data ref: NIH Bioproject PRJEB73699, 2024; C. elegans Sequencing Consortium (1998); Data ref: C. elegans Sequencing Consortium, 1998).

### RNA sequencing data analysis

RNA-seq data were downloaded from NCBI SRA for the following species: *H. bakeri* (Pollo et al, 2023; Data ref: Pollo et al, 2023), *N. brasiliensis* (Ferguson et al, 2023; Data ref: Ferguson et al, 2023; Chandler et al, 2017; Data ref: Chandler et al, 2017), *T. circumcincta* (Teladorsagia_circumcincta_transcriptomics (2015); Data ref: NIH Bioproject PRJEB7677, 2015) and *A. ceylanicum* (Schwarz et al, 2015; Data ref: Schwarz et al, 2015). FastQC v.0.12.1 (https://www.bioinformatics.babraham.ac.uk/projects/fastqc/) and MultiQC v.1.14 were used for quality control (Ewels et al, 2016). Although almost all libraries showed no quality issues, those corresponding to the *N. brasiliensis* PRJNA994163 (Data ref: Ferguson et al, 2023) showed high proportions ( ~ 15%) of Clontech SMART Primer II sequence and Illumina universal adapter. Thus, for these libraries, an extra trimming step was implemented with cutadapt v.4.9 (Martin, 2011) and parameters -g ^AAGCAGTGGTATCAAC -G ^AAGCAGTGGTATCAAC -m.

The selected RNA-seq reads were aligned to their respective genome using STAR v.2.7.10b (Dobin et al, 2013) with the gtf gene annotation files to define junctions (--sjdbGTFfile gtf_annotation_file --clip3pAdapterSeq AGATCGGAAGAGCACACGT AGATCGGAAGAGCGTCGTG --outFilterMismatchNmax 3 --outFilterScoreMinOverLread 0.3). After alignment, gene expression was quantified using the featureCounts function from the Rsubread R package (Liao et al, 2019) (allowMultiOverlap= TRUE, requireBothEndsMapped = TRUE, countChimericFragments = FALSE, useMetaFeatures=TRUE, fraction = TRUE). The resulting raw counts were normalised into Transcripts Per Million (TPM) using the convertCounts function from the DGEobj.utils R package (Thompson et al, 2022). Each biological replicate was treated individually. If more than one orthologue was identified, the gene locus with the highest mapped reads and highest percentage amino acid identity was used (Datasets EV2 and EV3).

### Statistical analysis

Data were checked for normality using the Shapiro-Wilk test. For detection of Hb-exWAGO via ELISA, optical density values from EVs and EV-depleted HES treated with and without detergent were normally distributed and were analysed using a two-way ANOVA with Šídák’s correction for selected comparisons. For the detection of Hb-exWAGO secreted by *H. bakeri* worms at different life stages, optical values were normally distributed and analysed using an unpaired two-tailed *t* test. The median fluorescence values from flow cytometry uptake assays were normally distributed and analysed using a repeated measures one-way ANOVA with Tukey’s multiple comparisons correction. Worm counts and faecal egg counts data were not normally distributed and were analysed using an unpaired Kruskal–Wallis test using uncorrected Dunn’s test for selected comparisons. For the detection of Hb-exWAGO-specific IgG1, optical values from 5 out of 6 groups compared were normally distributed. Upon assessing QQ plot there were no clear deviations from the expected normality, hence the data were analysed using one-way ANOVA with selected comparisons with Šídák’s multiple comparisons test. Labelled recombinant Hb-exWAGO uptake inhibition data were not normally distributed and were analysed using an unpaired Kruskal–Wallis test with Dunn’s multiple comparisons test. Data analyses were performed using GraphPad Prism software version 10.1.2. Significance between two groups is denoted using asterisks (ns = non-significant, *P  <  0.05, **P  <  0.01, ***P  <  0.001, ****P  <  0.0001).

## Supplementary information

Peer Review File

Dataset EV1

Dataset EV2

Dataset EV3

Dataset EV4

Source data Fig. 1

Source data Fig. 2

Source data Fig. 3

Source data Fig. 4

Source data Fig. 5

Expanded View Figures

## Data Availability

The small RNA sequencing data are deposited to the National Center for Biotechnology Information (NCBI) Sequence Read Archive (SRA) under the Bioproject ID PRJNA1200757: https://www.ncbi.nlm.nih.gov/sra/?term=PRJNA1200757. R scripts for analysis of the small RNA libraries are available at https://github.com/imu93/exwago_vaccine. The source data of this paper are collected in the following database record: biostudies:S-SCDT-10_1038-S44319-025-00620-4.
