# Supplementary figures and images for: An Argonaute protein traffics from nematode to mouse and is a vaccine against parasitic nematodes
Source: EMBO Rep. 2025 Dec 9;27(2):311–40. doi: 10.1038/s44319-025-00620-4 (PMC12852730; doi:10.1038/s44319-025-00620-4)

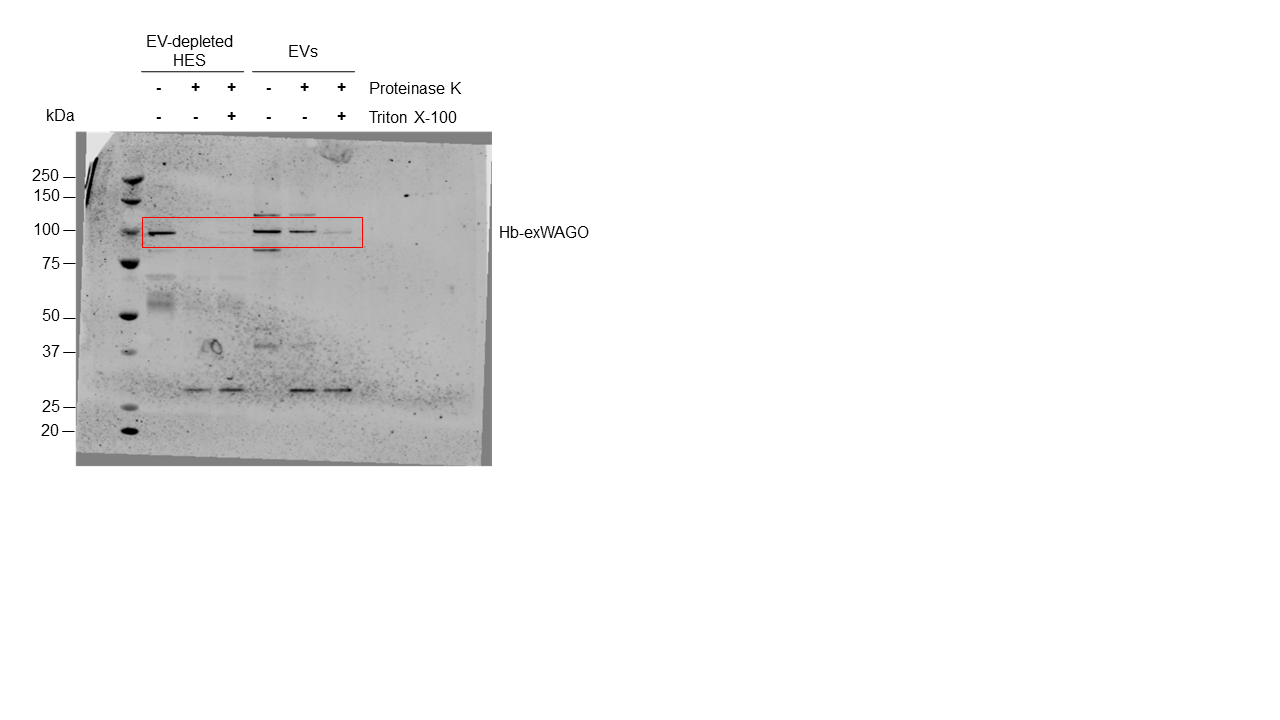

Supplement: Supplementary file 6 — Source data Fig. 1 [file 44319_2025_620_MOESM6_ESM.zip › Figure 1/1A/EMBOR-2025-61666V_1A_western anti-exWAGO.tif.tif]

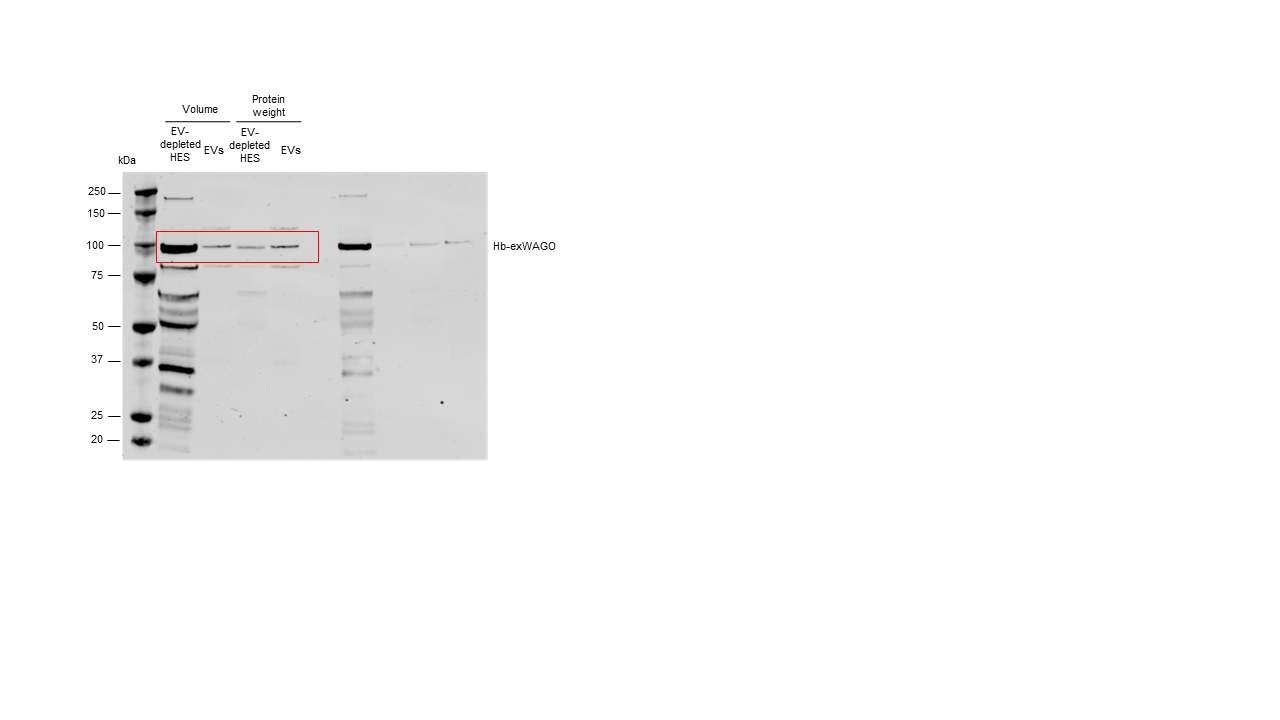

Supplement: Supplementary file 6 — Source data Fig. 1 [file 44319_2025_620_MOESM6_ESM.zip › Figure 1/1D/EMBOR-2025-61666V_1D_western anti-exWAGO.tif.tif]

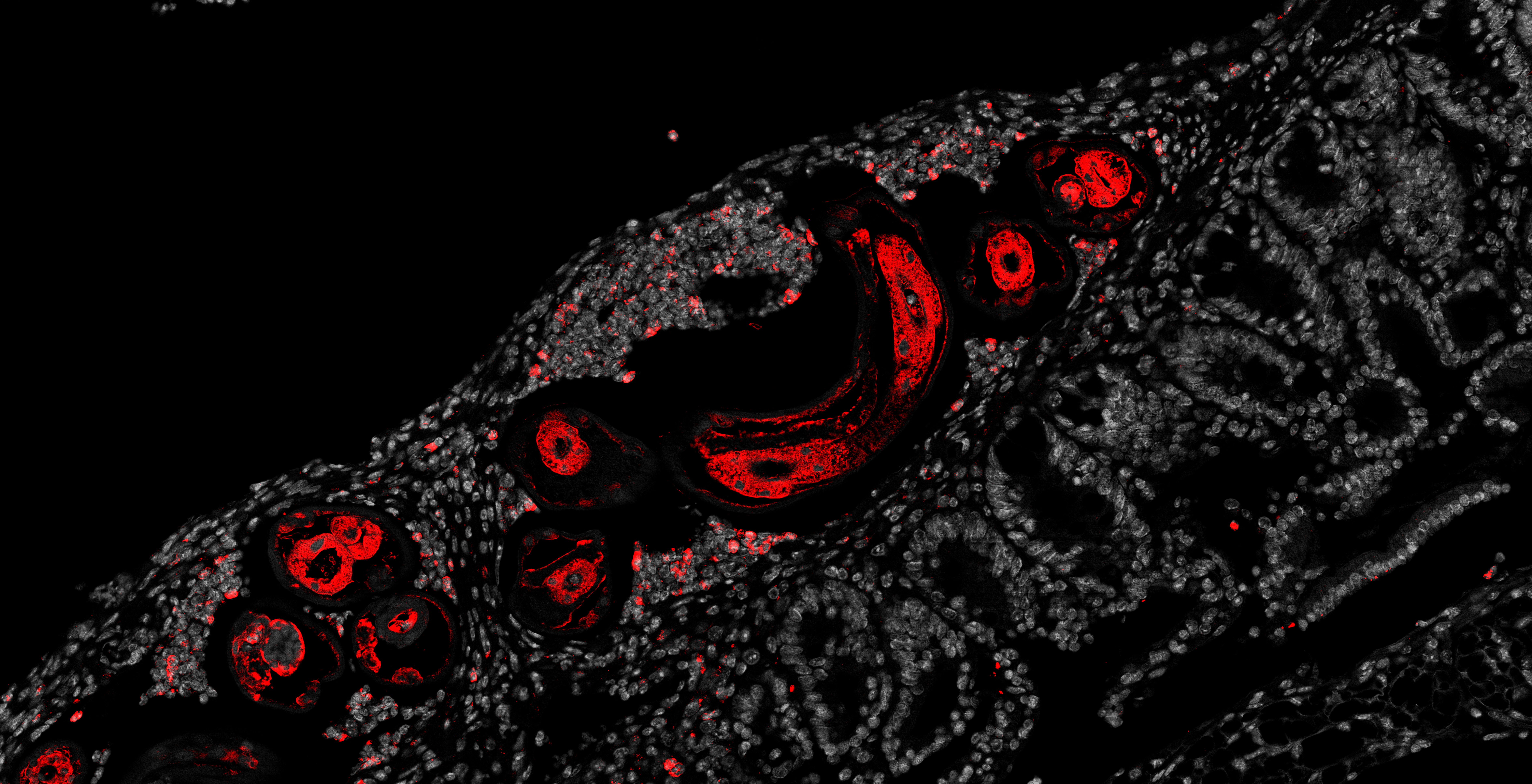

Supplement: Supplementary file 8 — Source data Fig. 3 [file 44319_2025_620_MOESM8_ESM.zip › Figure 3/3A/EMBOR-2025-61666V_Infected mouse_anti-Hb-exWAGO.tif.tif]

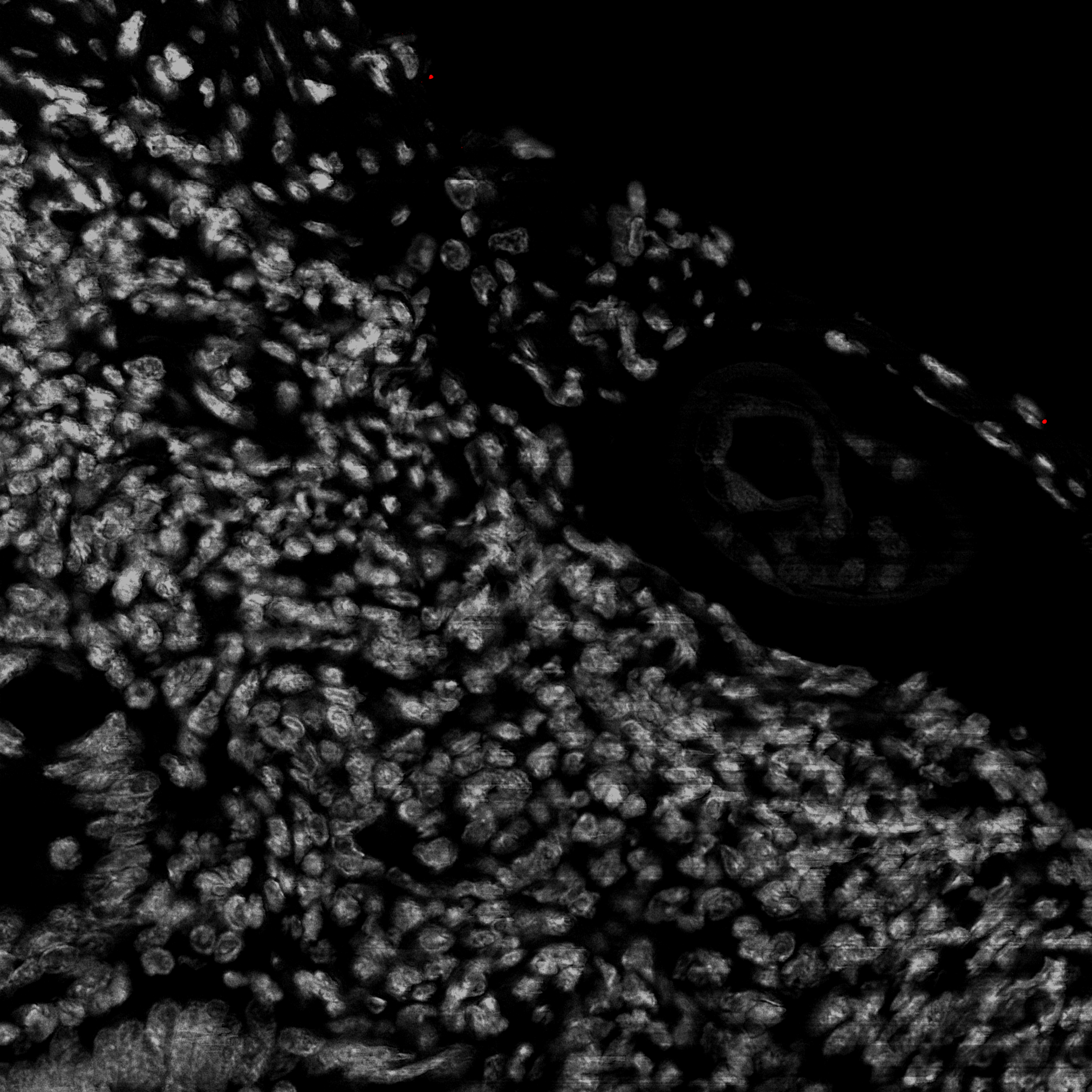

Supplement: Supplementary file 8 — Source data Fig. 3 [file 44319_2025_620_MOESM8_ESM.zip › Figure 3/3A/EMBOR-2025-61666V_Infected mouse_anti-IgG.tif.tif]

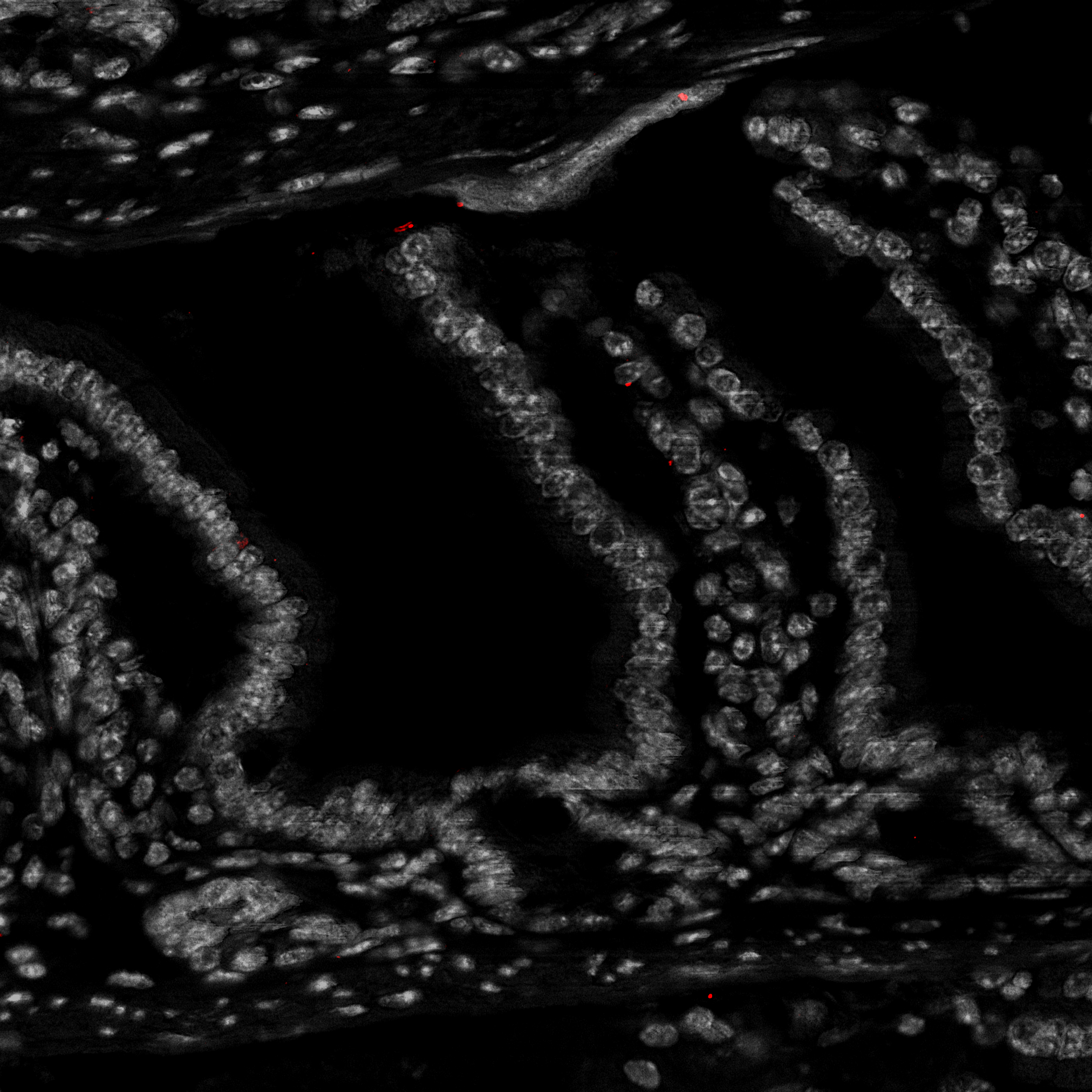

Supplement: Supplementary file 8 — Source data Fig. 3 [file 44319_2025_620_MOESM8_ESM.zip › Figure 3/3A/EMBOR-2025-61666V_Naive mouse_anti-Hb-exWAGO.tif.tif]

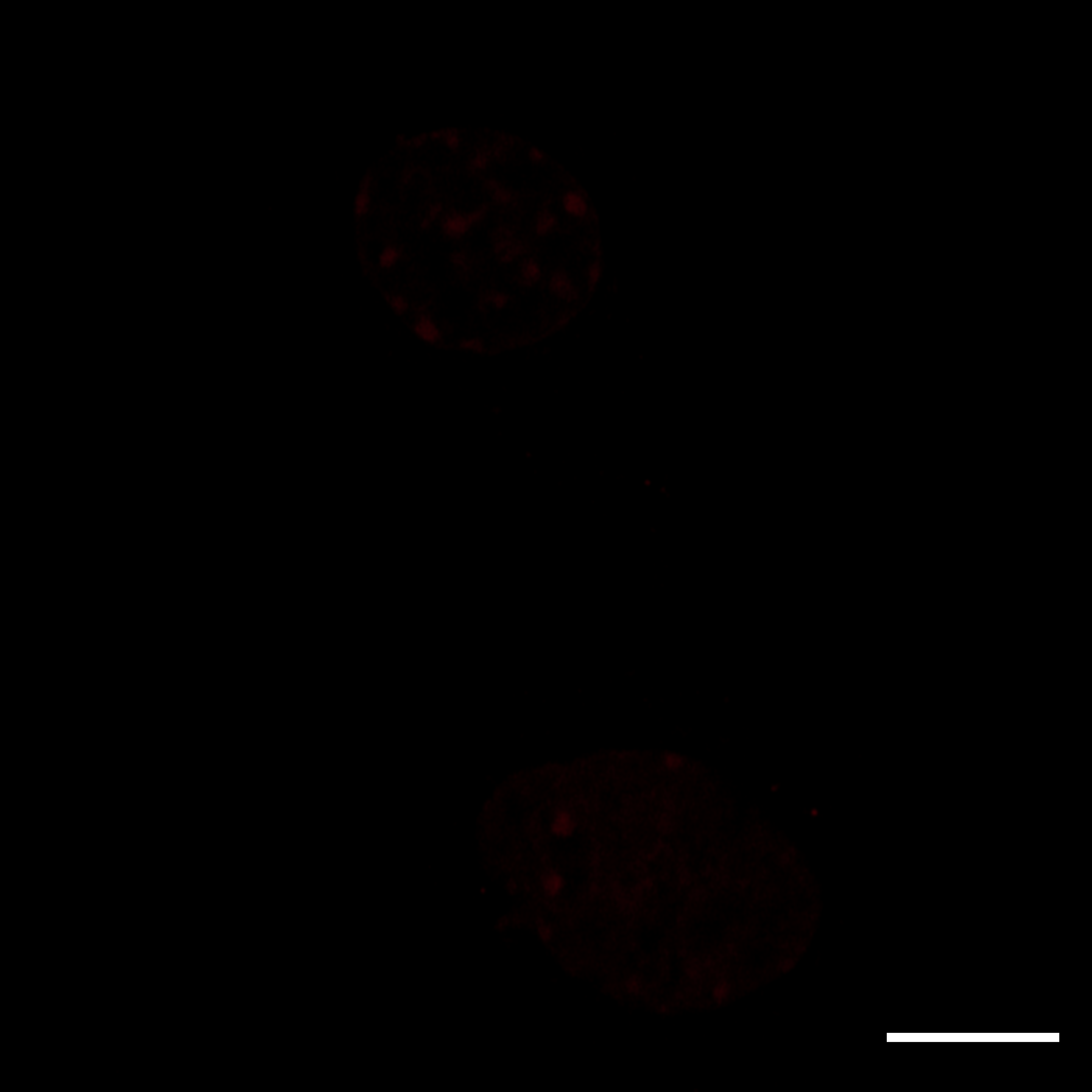

Supplement: Supplementary file 8 — Source data Fig. 3 [file 44319_2025_620_MOESM8_ESM.zip › Figure 3/3B/EMBOR-2025-61666V_MODEK_BSA_AF647-T1.tif.tif]

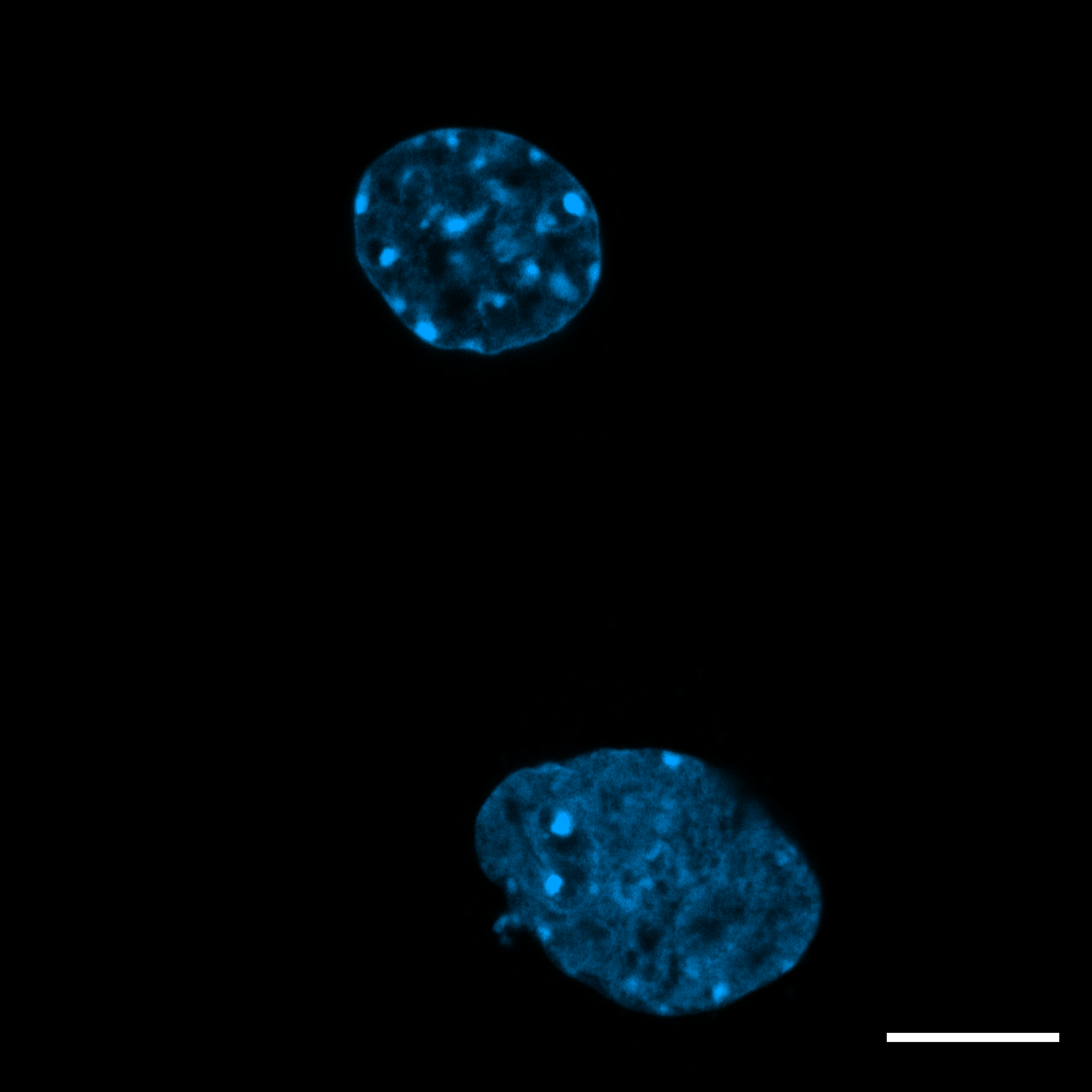

Supplement: Supplementary file 8 — Source data Fig. 3 [file 44319_2025_620_MOESM8_ESM.zip › Figure 3/3B/EMBOR-2025-61666V_MODEK_BSA_DAPI-T3.tif.tif]

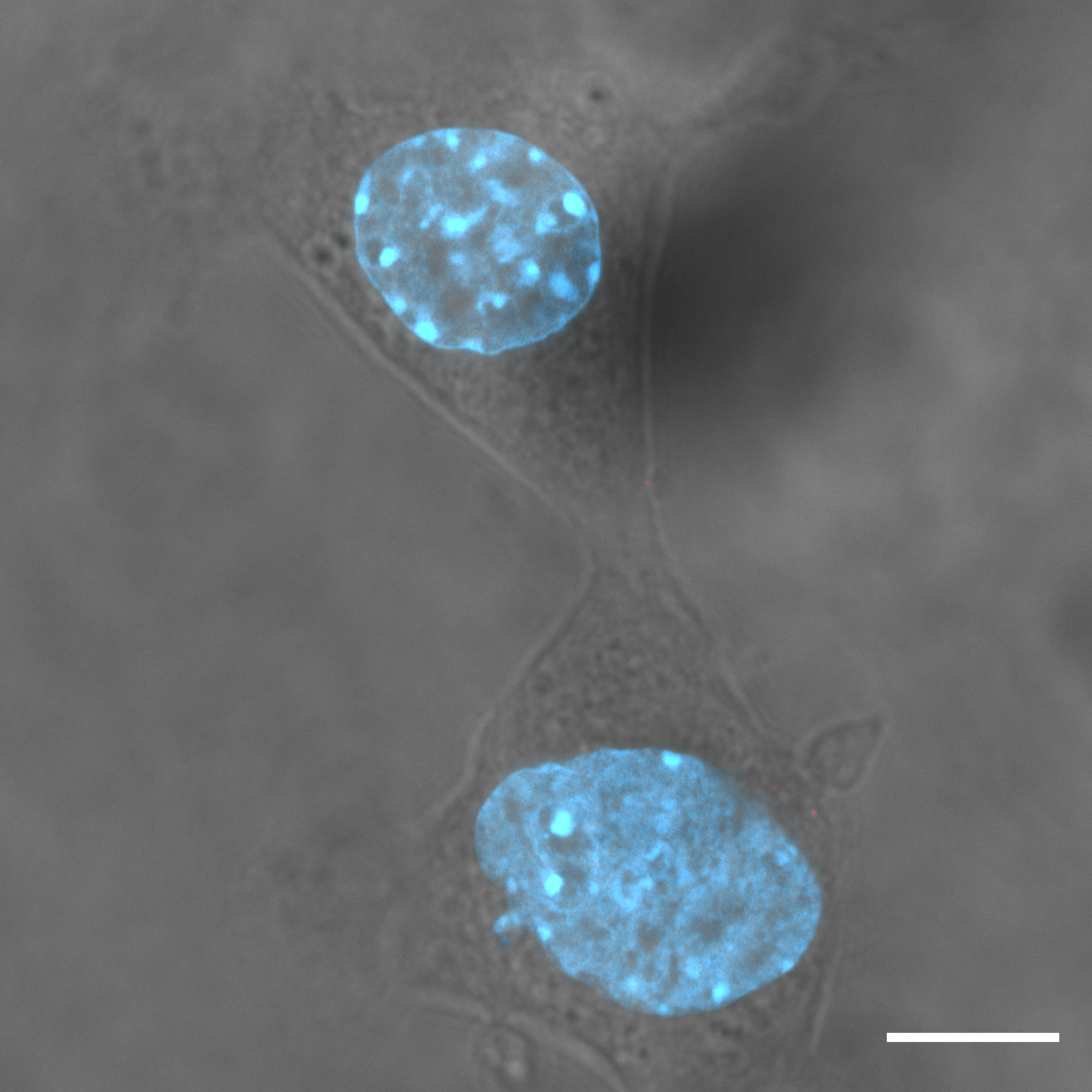

Supplement: Supplementary file 8 — Source data Fig. 3 [file 44319_2025_620_MOESM8_ESM.zip › Figure 3/3B/EMBOR-2025-61666V_MODEK_BSA_Merge.tif.tif]

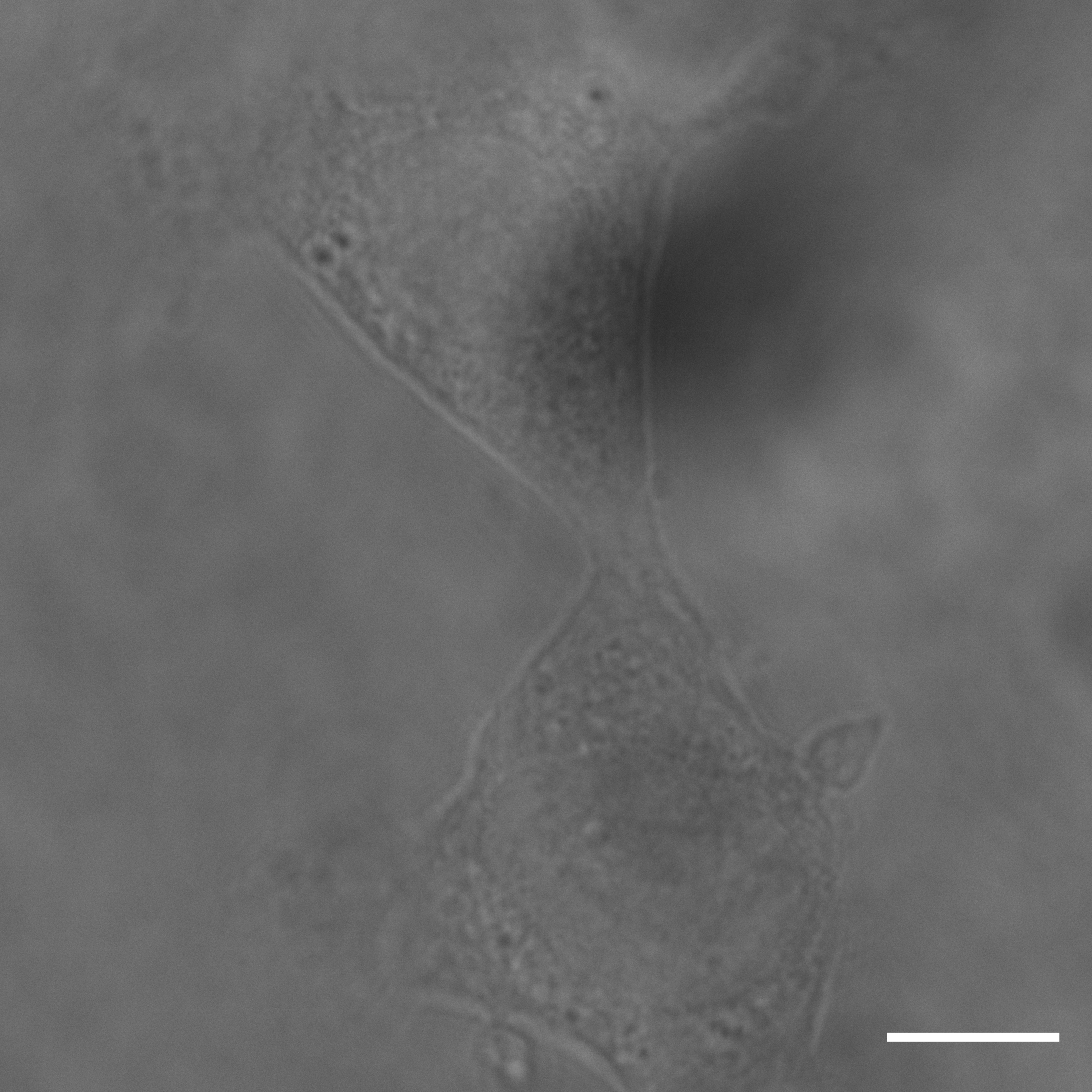

Supplement: Supplementary file 8 — Source data Fig. 3 [file 44319_2025_620_MOESM8_ESM.zip › Figure 3/3B/EMBOR-2025-61666V_MODEK_BSA_T-PMT-T4.tif.tif]

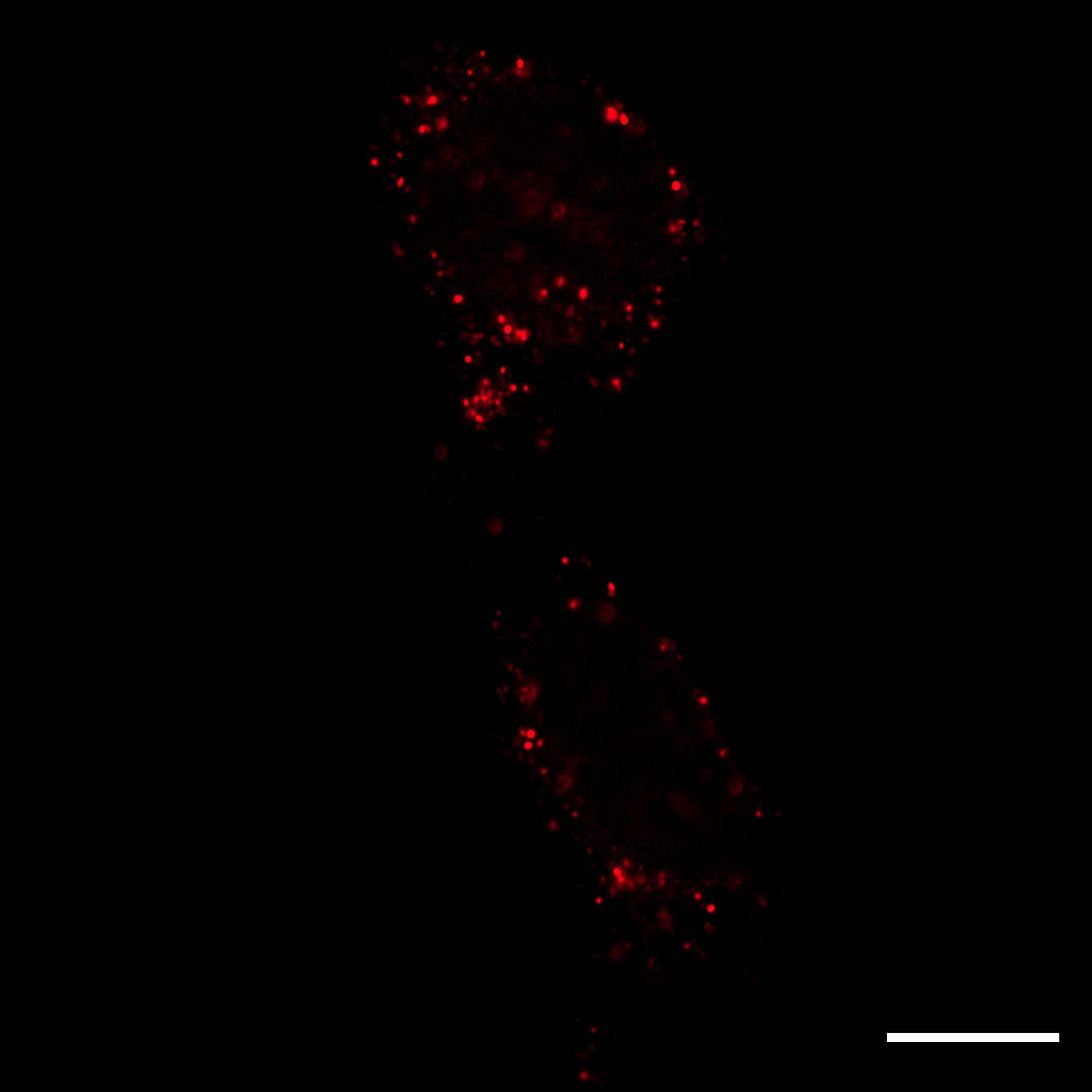

Supplement: Supplementary file 8 — Source data Fig. 3 [file 44319_2025_620_MOESM8_ESM.zip › Figure 3/3B/EMBOR-2025-61666V_MODEK_Hb-rexWAGO_AF647-T1.tif.tif]

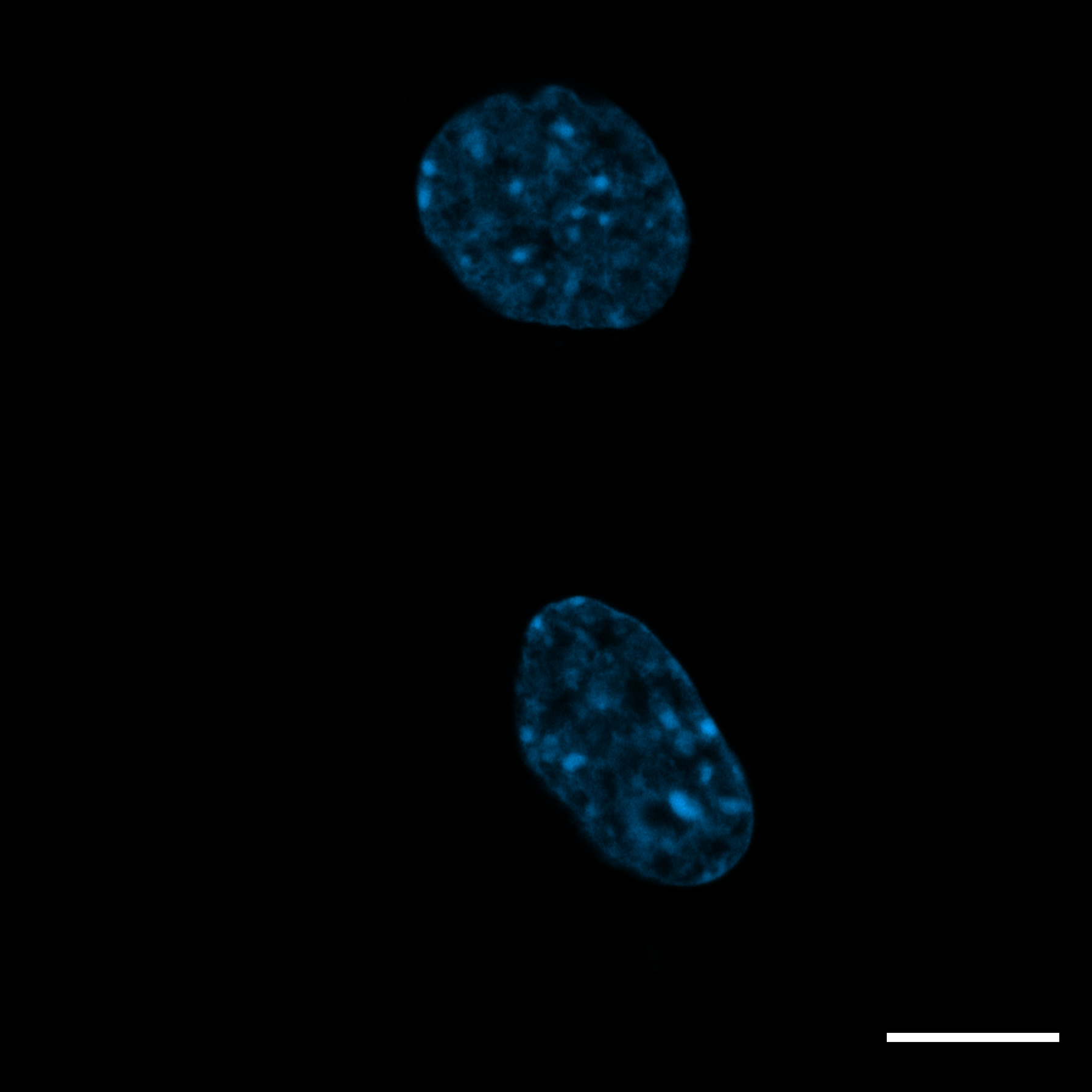

Supplement: Supplementary file 8 — Source data Fig. 3 [file 44319_2025_620_MOESM8_ESM.zip › Figure 3/3B/EMBOR-2025-61666V_MODEK_Hb-rexWAGO_DAPI-T3.tif.tif]

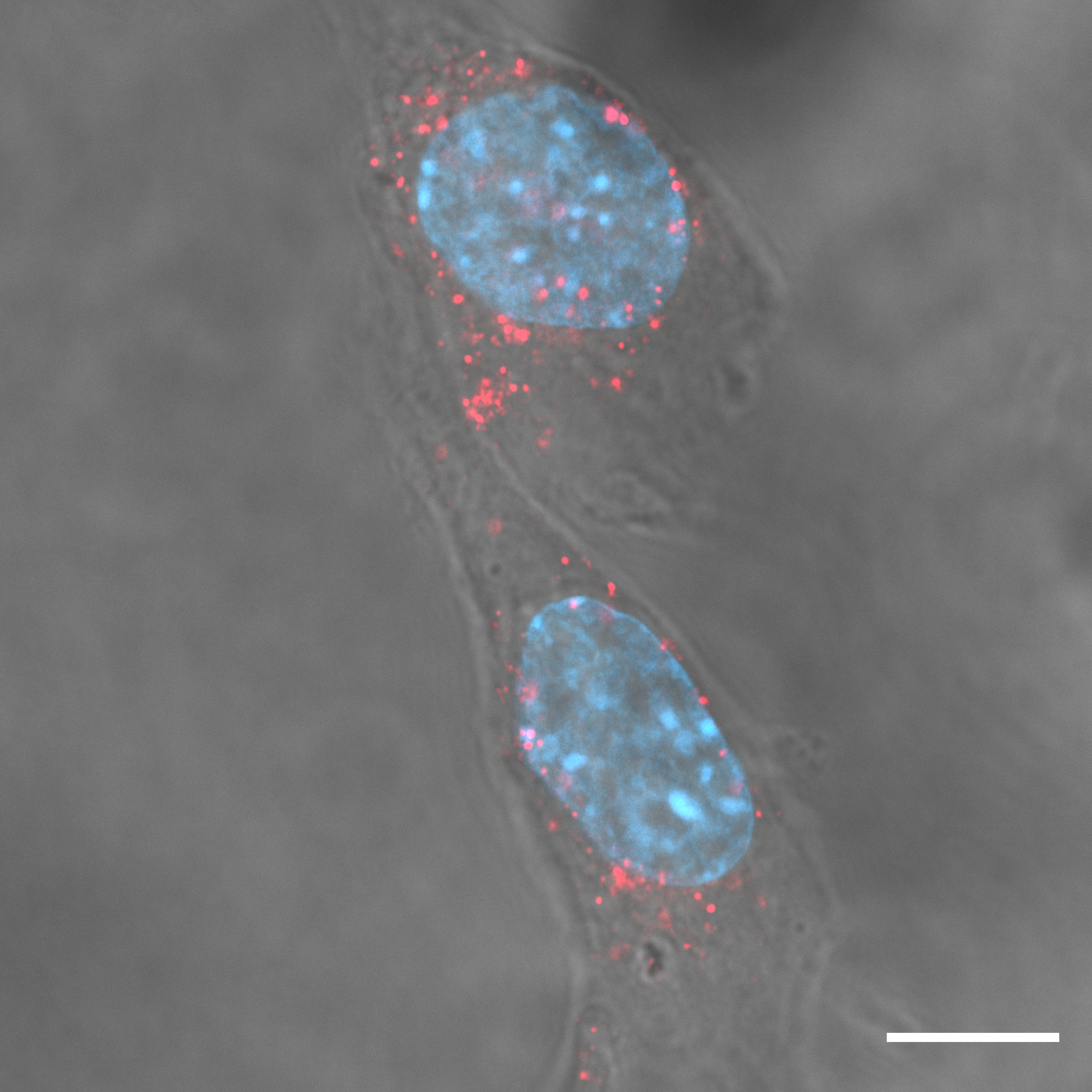

Supplement: Supplementary file 8 — Source data Fig. 3 [file 44319_2025_620_MOESM8_ESM.zip › Figure 3/3B/EMBOR-2025-61666V_MODEK_Hb-rexWAGO_Merge.tif.tif]

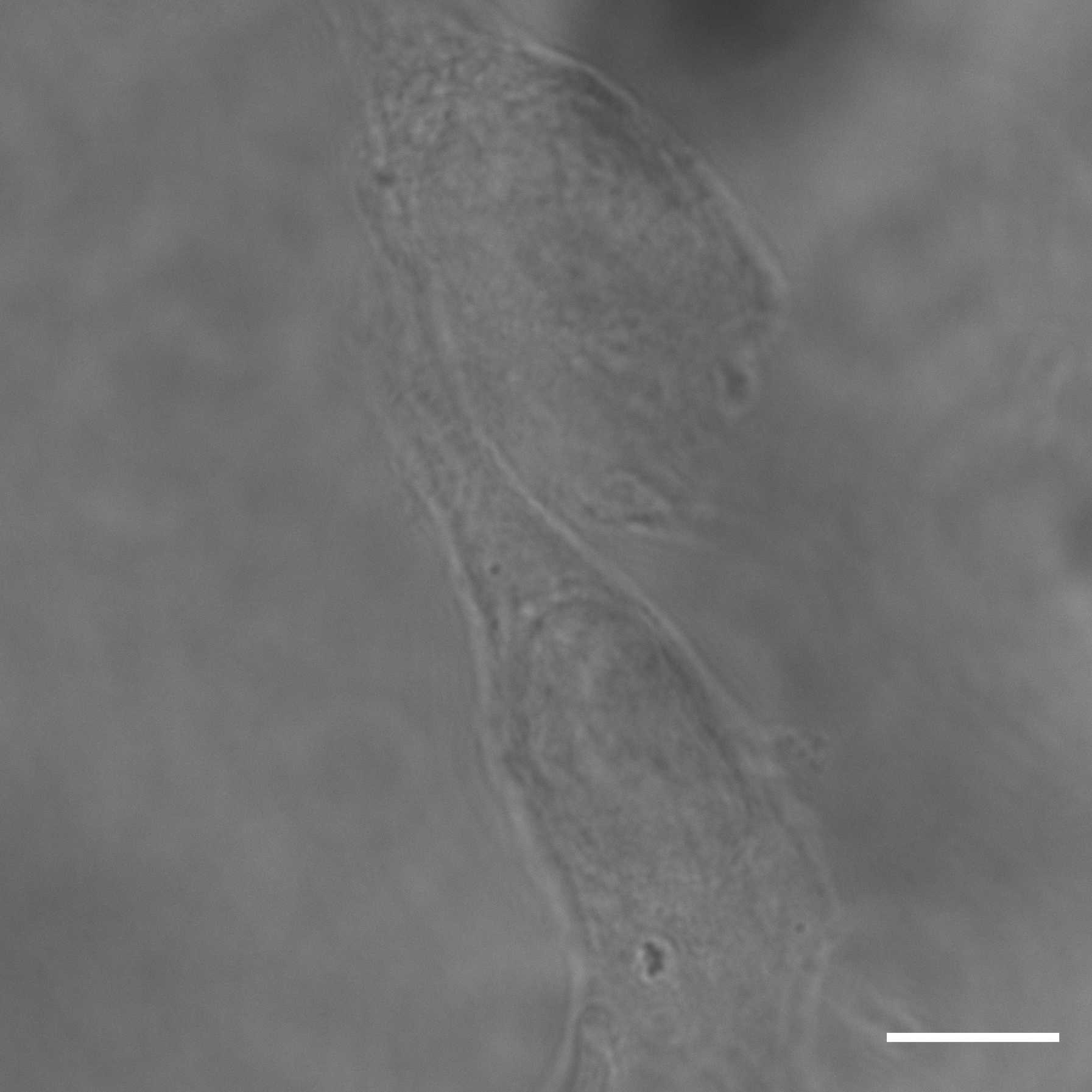

Supplement: Supplementary file 8 — Source data Fig. 3 [file 44319_2025_620_MOESM8_ESM.zip › Figure 3/3B/EMBOR-2025-61666V_MODEK_Hb-rexWAGO_T-PMT-T4.tif.tif]

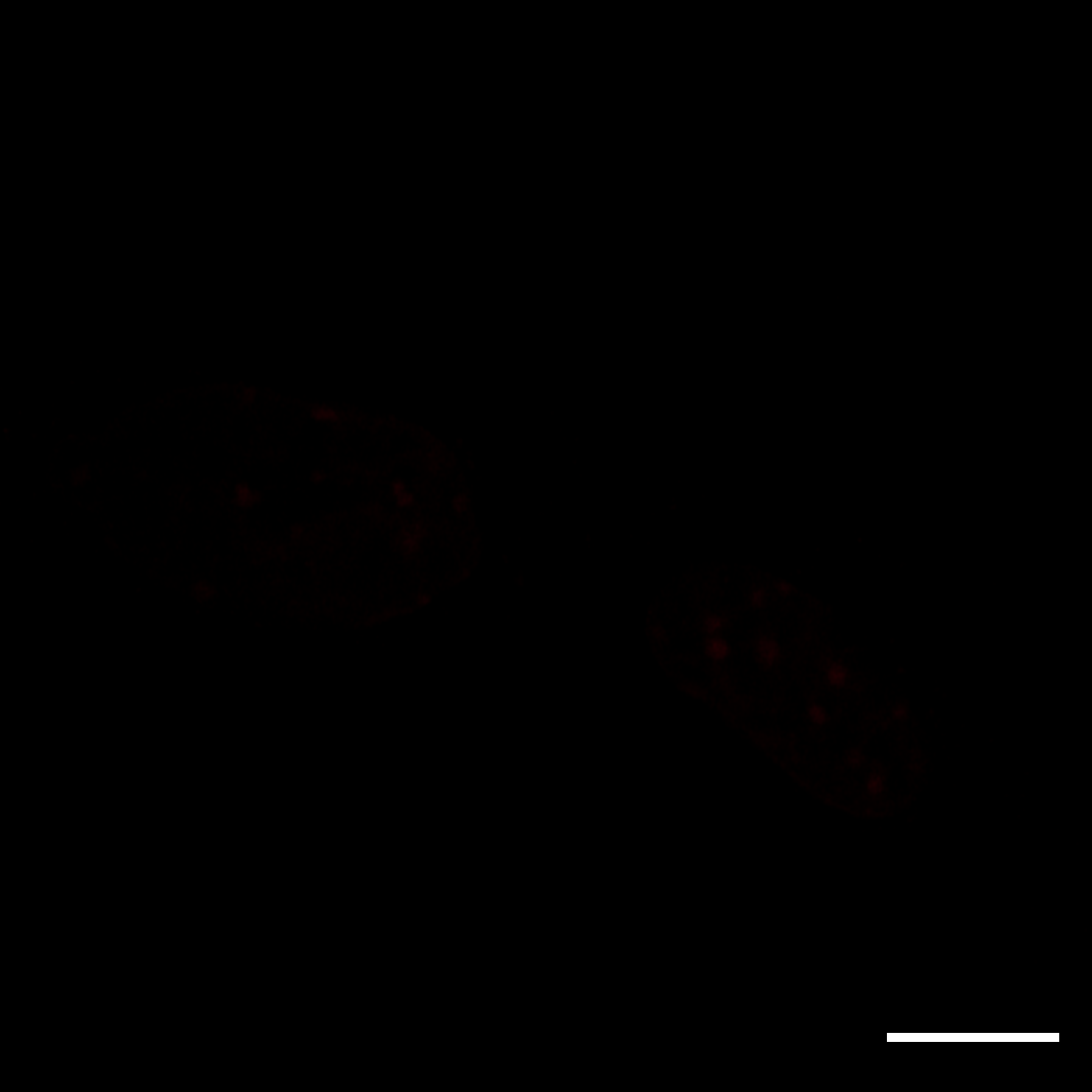

Supplement: Supplementary file 8 — Source data Fig. 3 [file 44319_2025_620_MOESM8_ESM.zip › Figure 3/3B/EMBOR-2025-61666V_MODEK_PBS_AF647-T1.tif.tif]

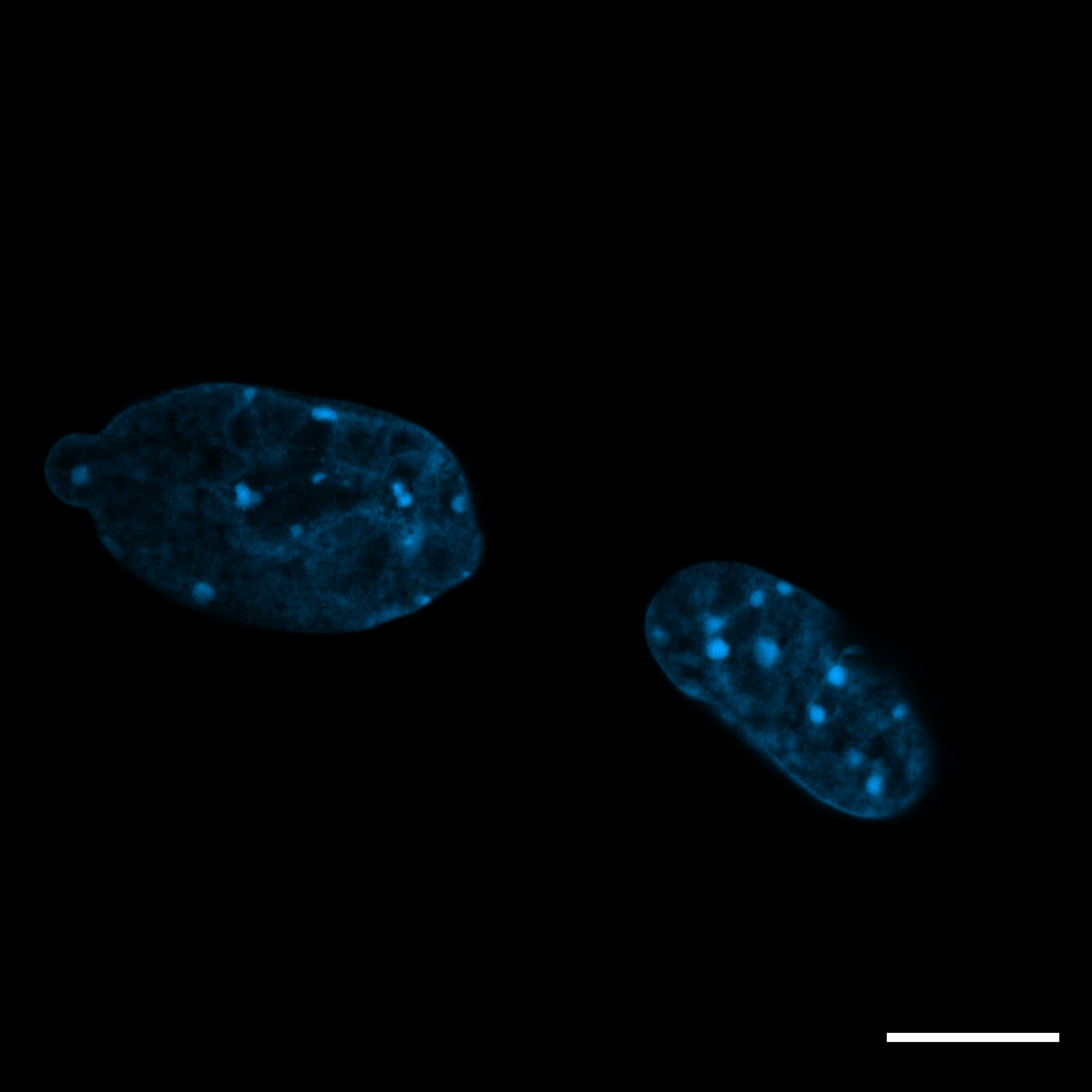

Supplement: Supplementary file 8 — Source data Fig. 3 [file 44319_2025_620_MOESM8_ESM.zip › Figure 3/3B/EMBOR-2025-61666V_MODEK_PBS_DAPI-T3.tif.tif]

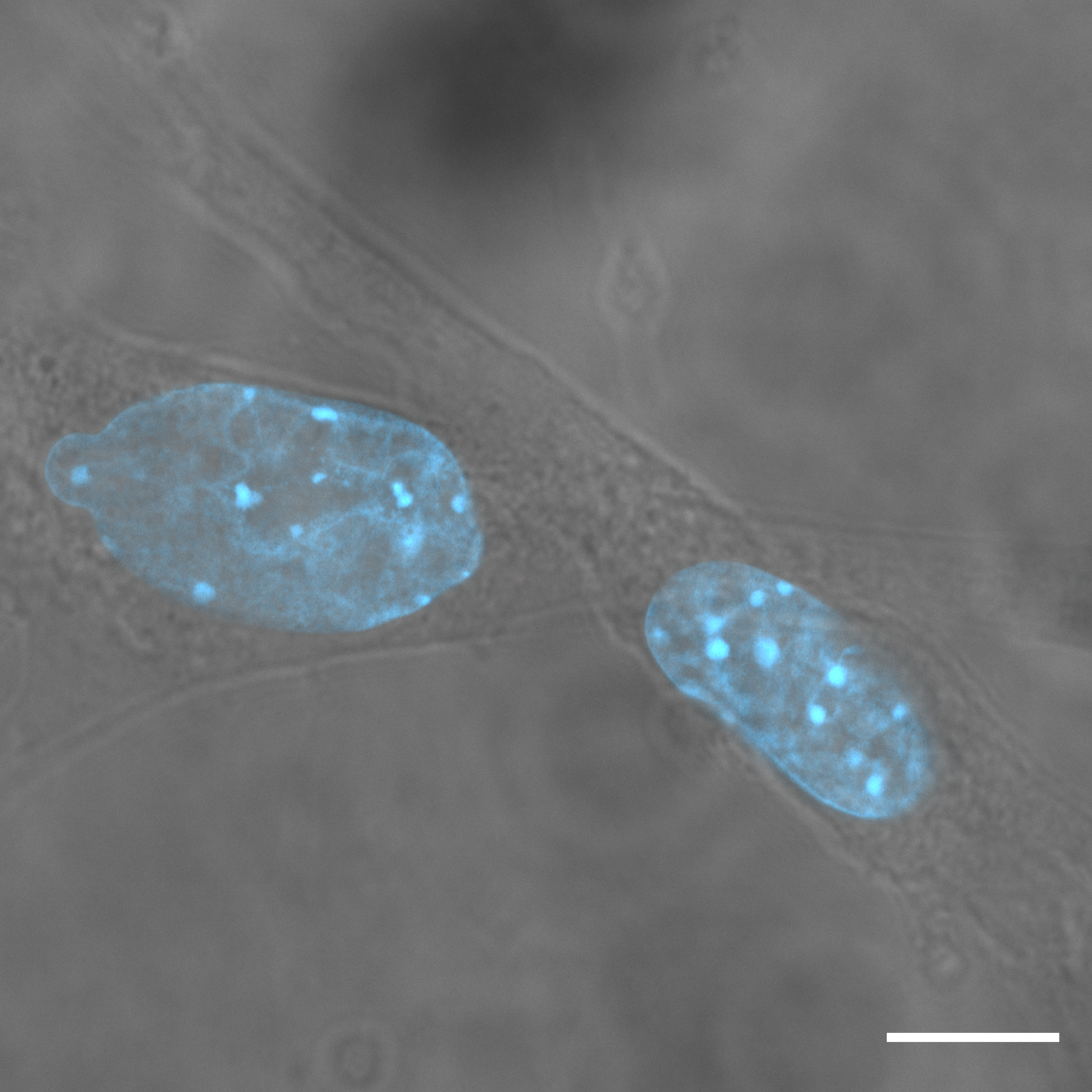

Supplement: Supplementary file 8 — Source data Fig. 3 [file 44319_2025_620_MOESM8_ESM.zip › Figure 3/3B/EMBOR-2025-61666V_MODEK_PBS_Merge.tif.tif]

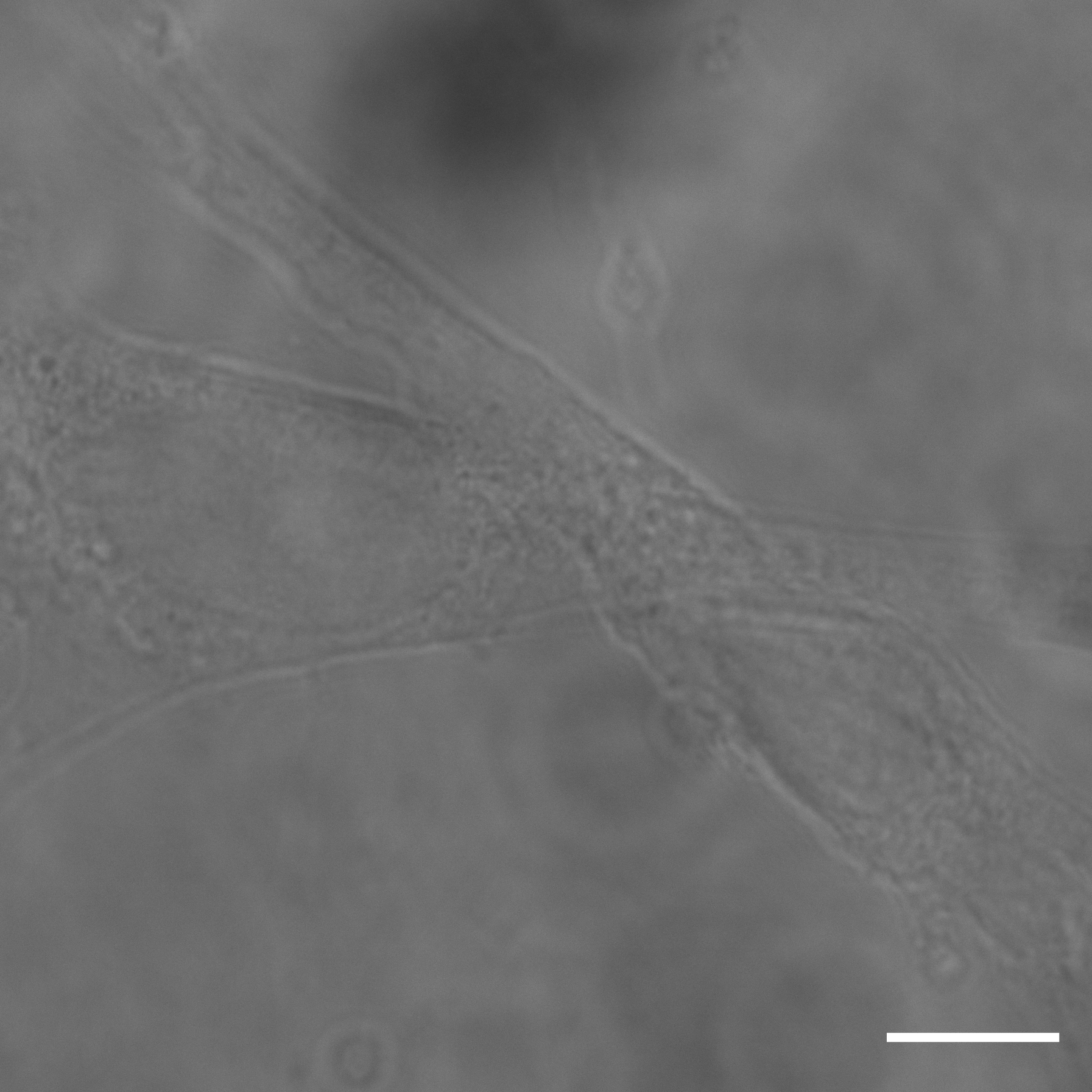

Supplement: Supplementary file 8 — Source data Fig. 3 [file 44319_2025_620_MOESM8_ESM.zip › Figure 3/3B/EMBOR-2025-61666V_MODEK_PBS_T-PMT-T4.tif.tif]

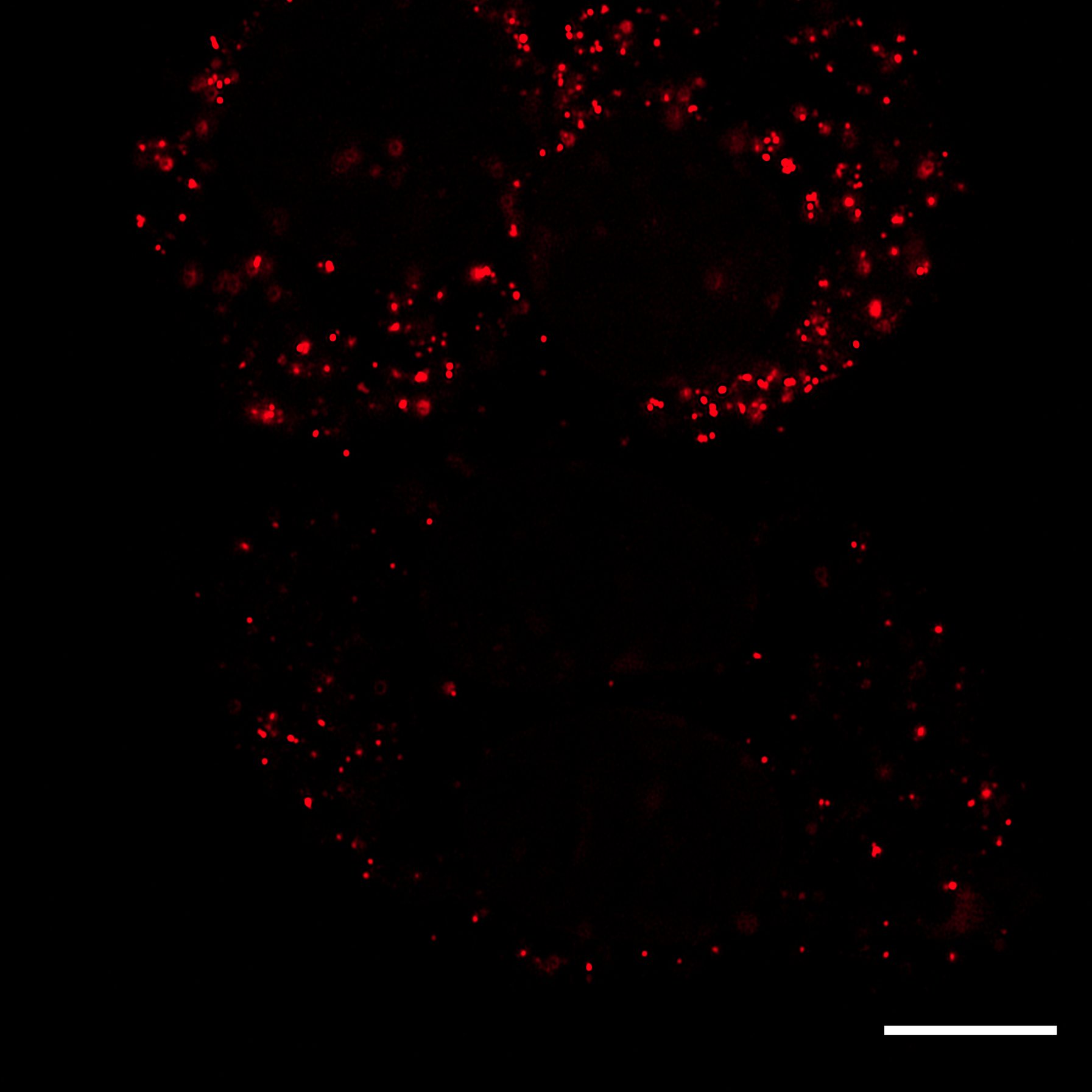

Supplement: Supplementary file 8 — Source data Fig. 3 [file 44319_2025_620_MOESM8_ESM.zip › Figure 3/3B/EMBOR-2025-61666V_RAW_BSA_AF647-T1.tif.tif]

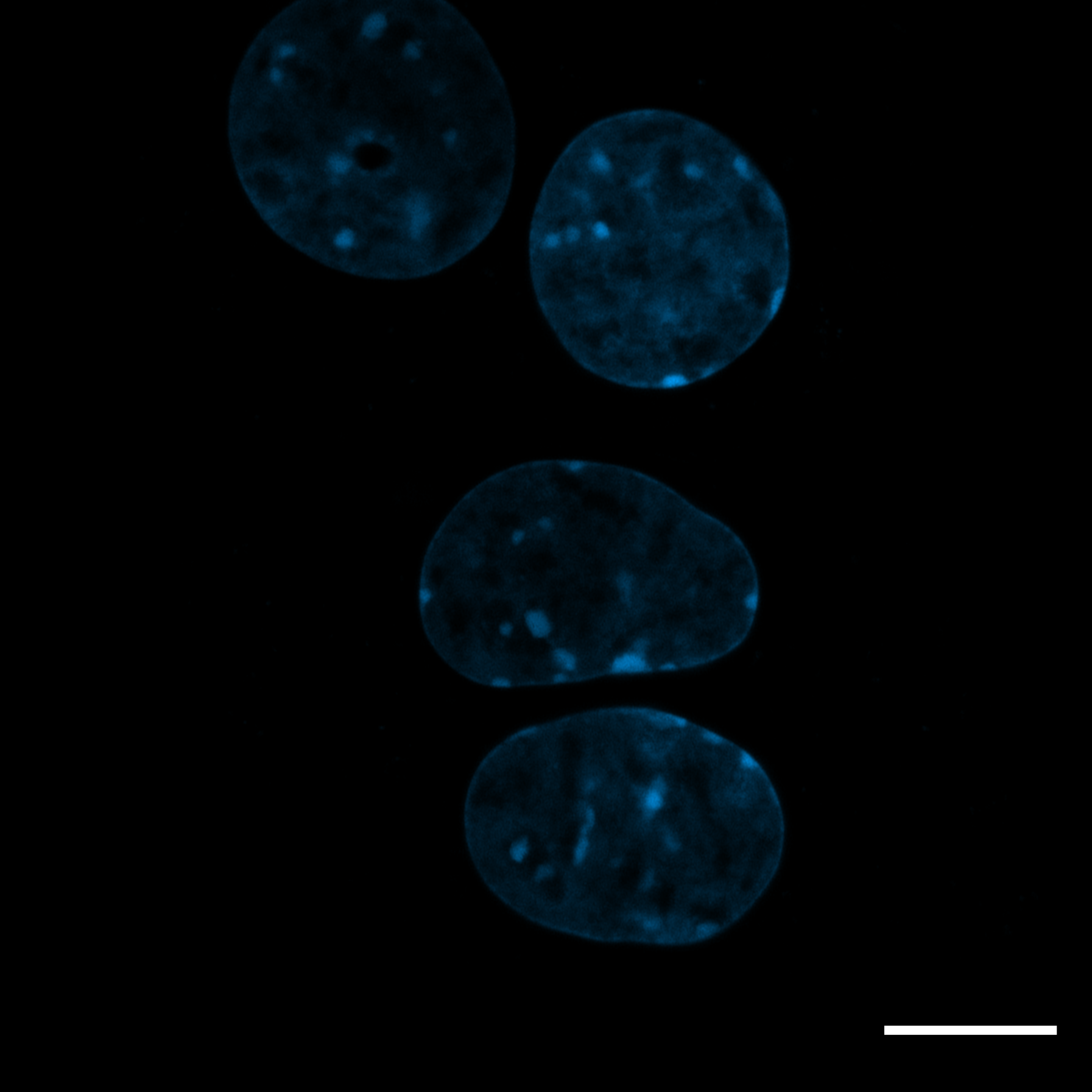

Supplement: Supplementary file 8 — Source data Fig. 3 [file 44319_2025_620_MOESM8_ESM.zip › Figure 3/3B/EMBOR-2025-61666V_RAW_BSA_DAPI-T3.tif.tif]

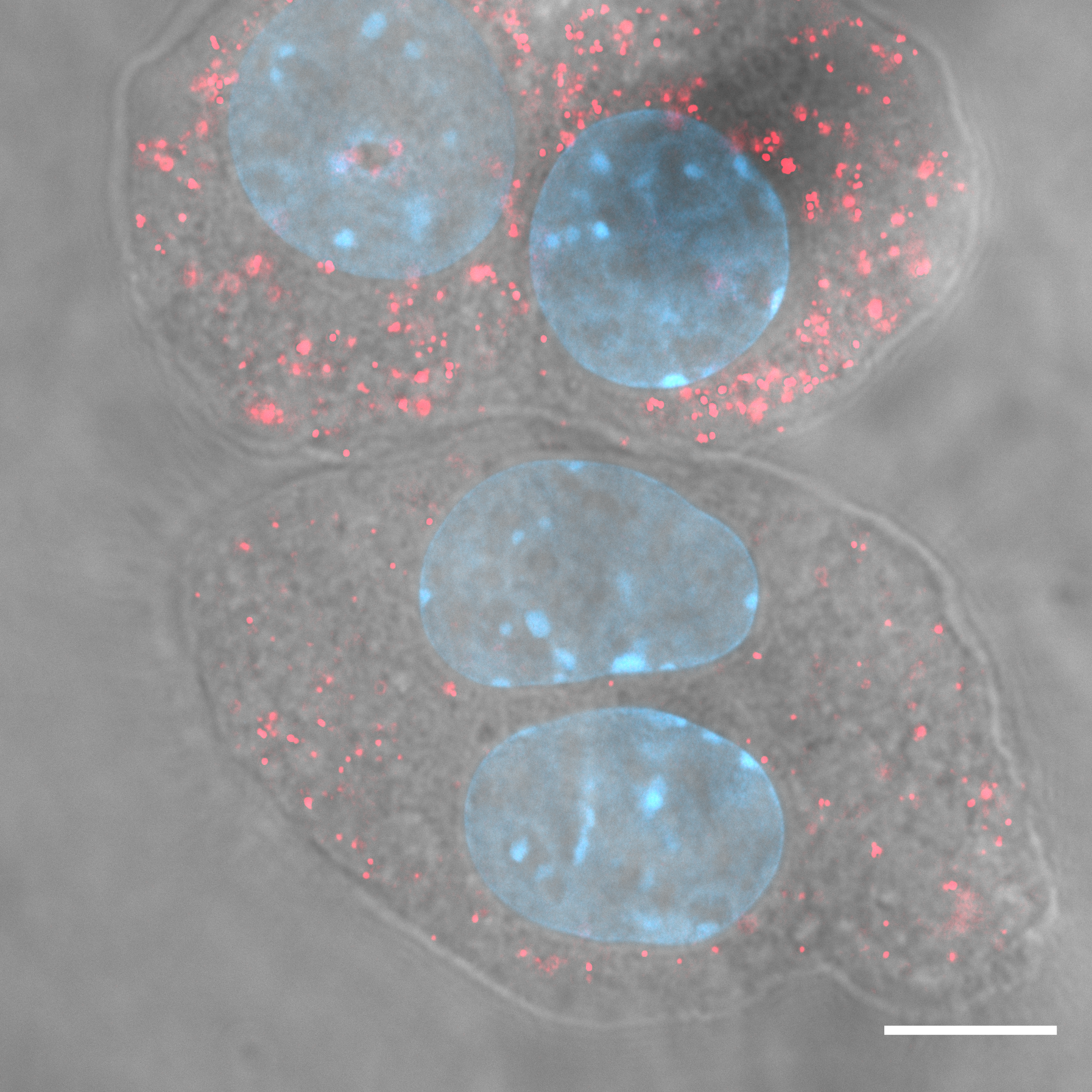

Supplement: Supplementary file 8 — Source data Fig. 3 [file 44319_2025_620_MOESM8_ESM.zip › Figure 3/3B/EMBOR-2025-61666V_RAW_BSA_Merge.tif.tif]

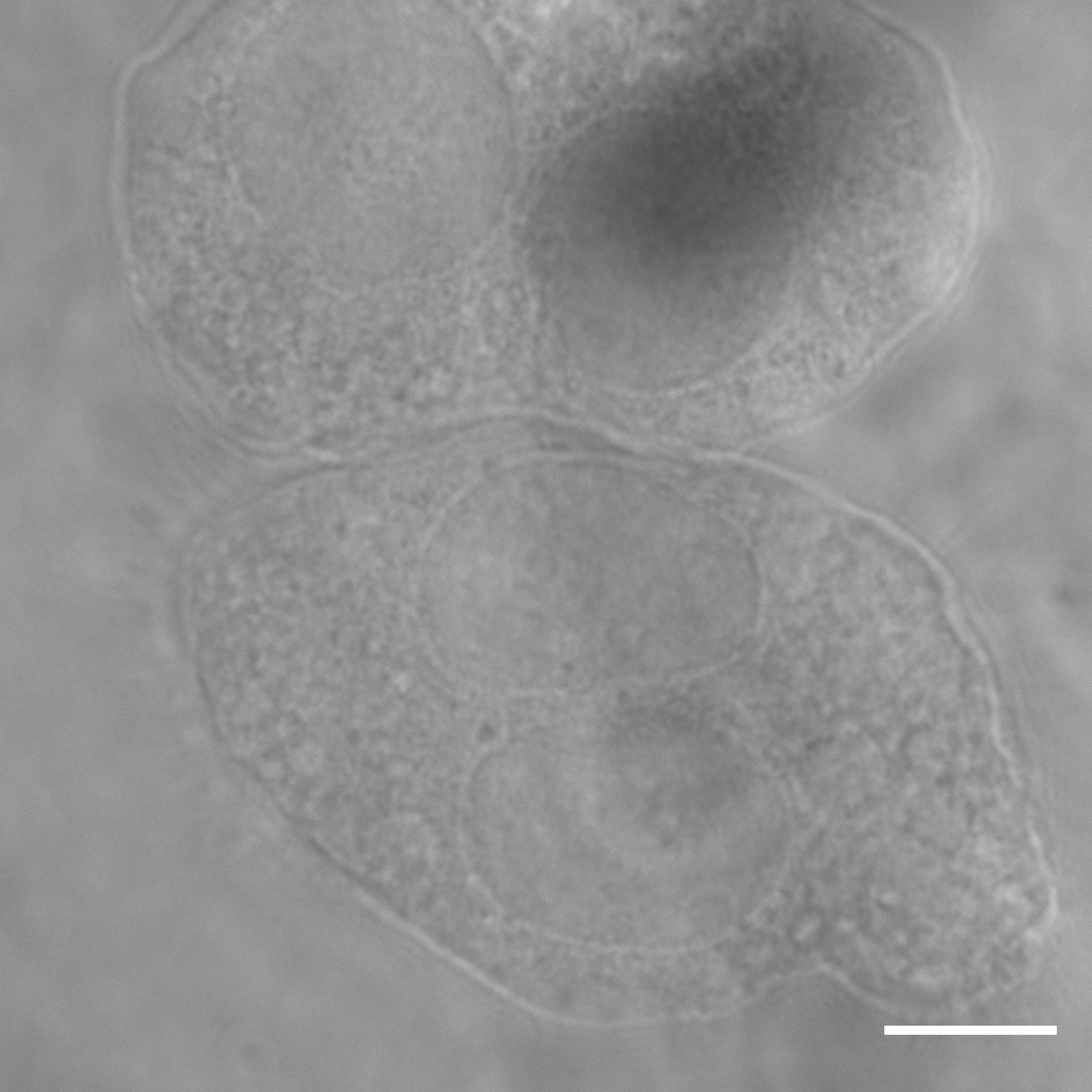

Supplement: Supplementary file 8 — Source data Fig. 3 [file 44319_2025_620_MOESM8_ESM.zip › Figure 3/3B/EMBOR-2025-61666V_RAW_BSA_T-PMT-T4.tif.tif]

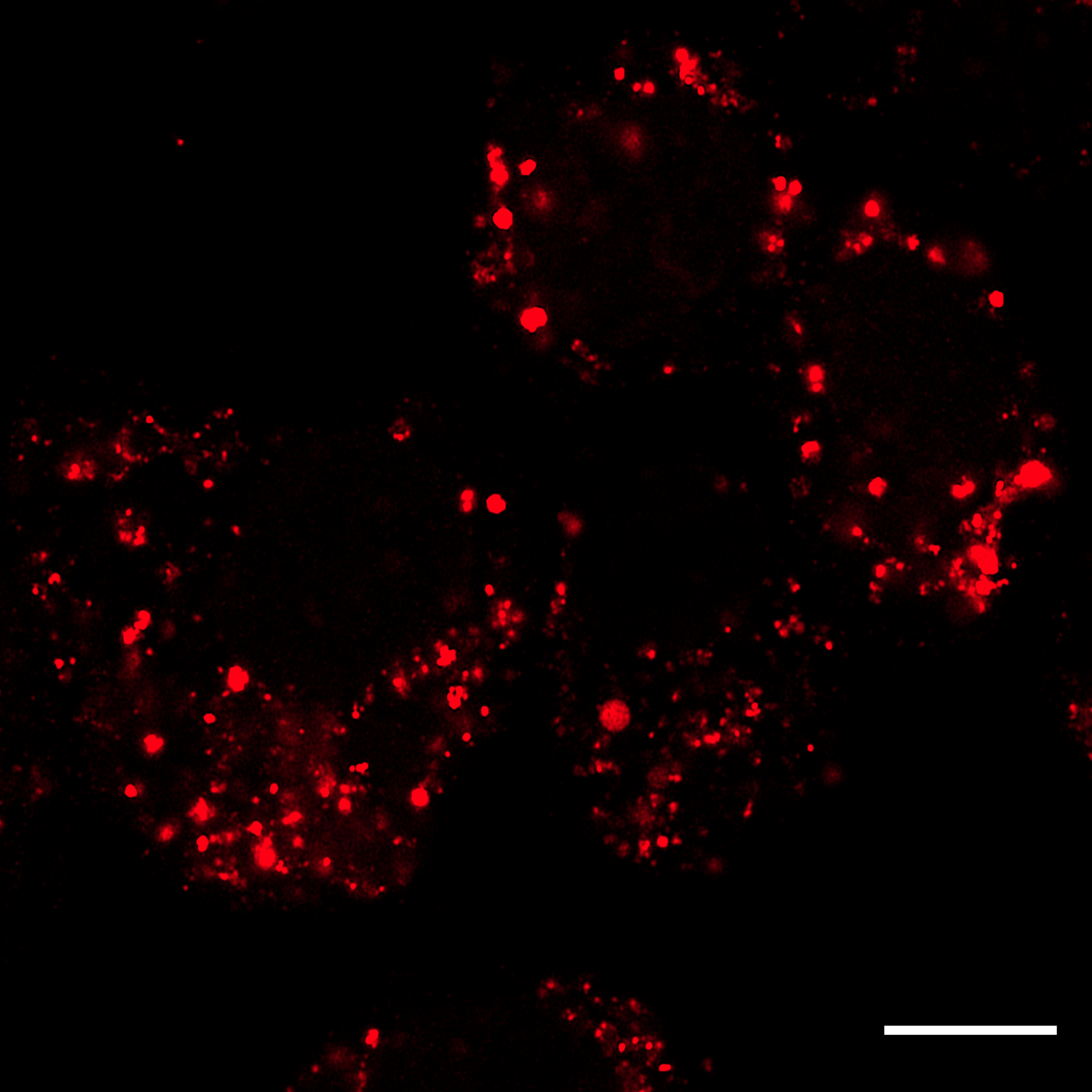

Supplement: Supplementary file 8 — Source data Fig. 3 [file 44319_2025_620_MOESM8_ESM.zip › Figure 3/3B/EMBOR-2025-61666V_RAW_Hb-rexWAGO_AF647-T1.tif.tif]

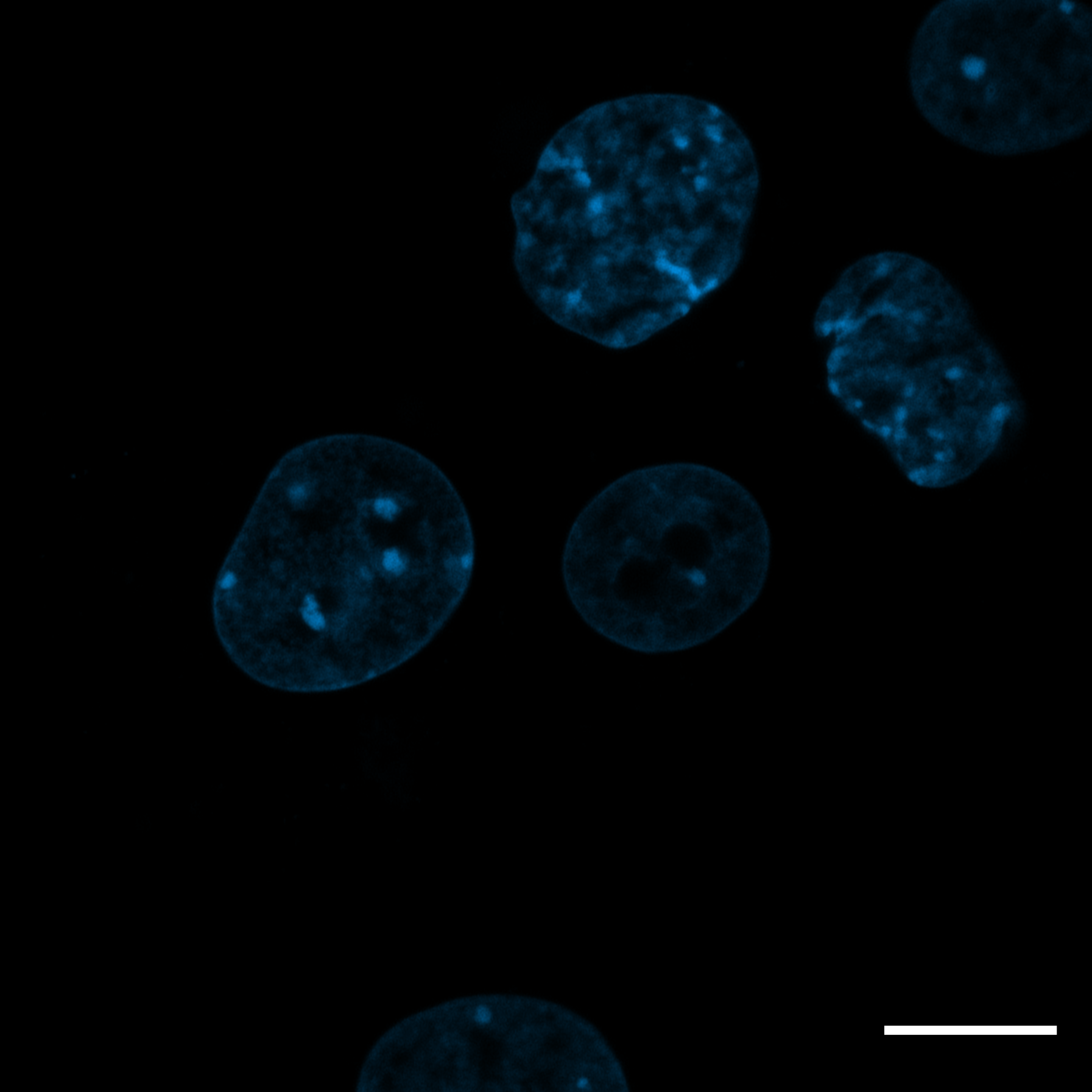

Supplement: Supplementary file 8 — Source data Fig. 3 [file 44319_2025_620_MOESM8_ESM.zip › Figure 3/3B/EMBOR-2025-61666V_RAW_Hb-rexWAGO_DAPI-T3.tif.tif]

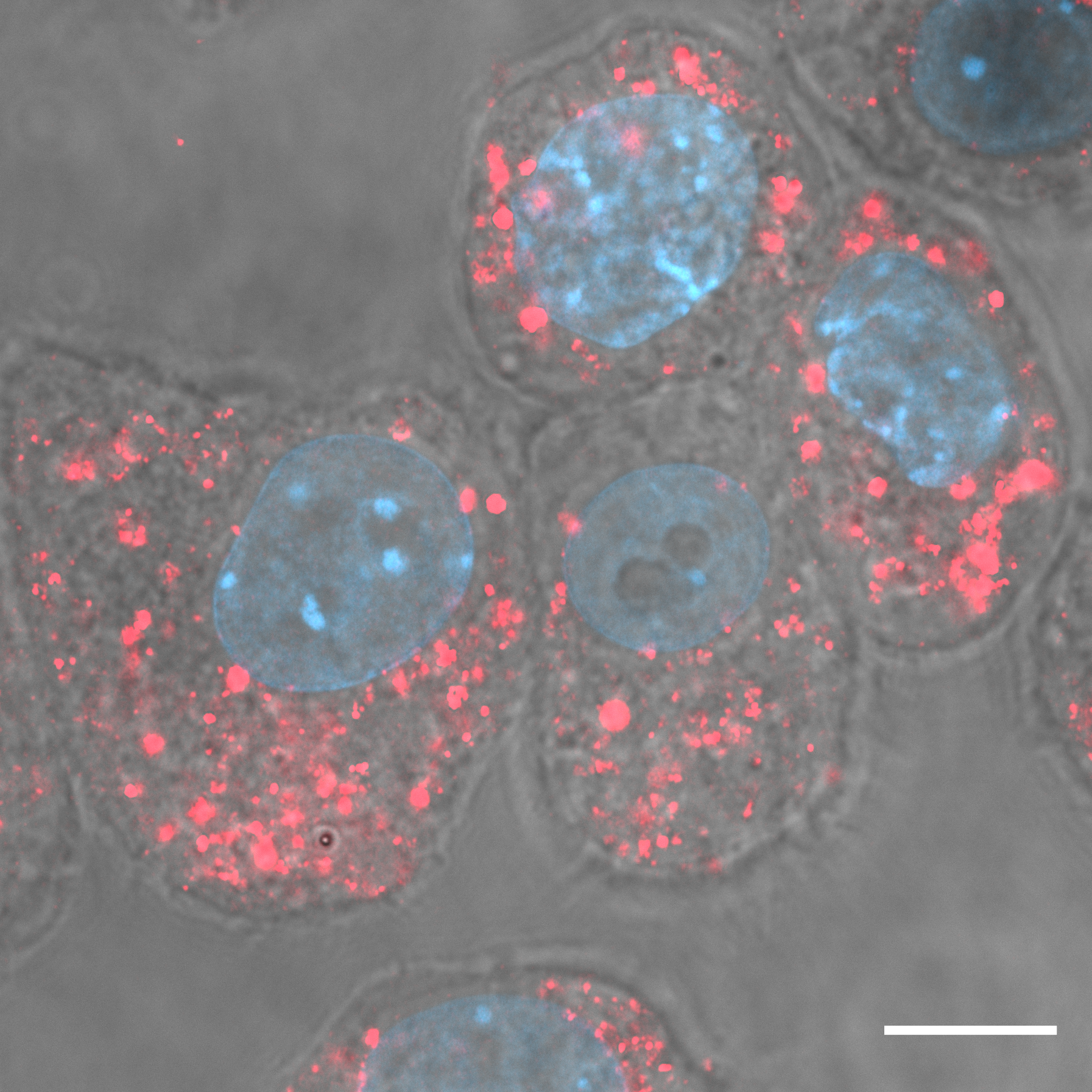

Supplement: Supplementary file 8 — Source data Fig. 3 [file 44319_2025_620_MOESM8_ESM.zip › Figure 3/3B/EMBOR-2025-61666V_RAW_Hb-rexWAGO_Merge.tif.tif]

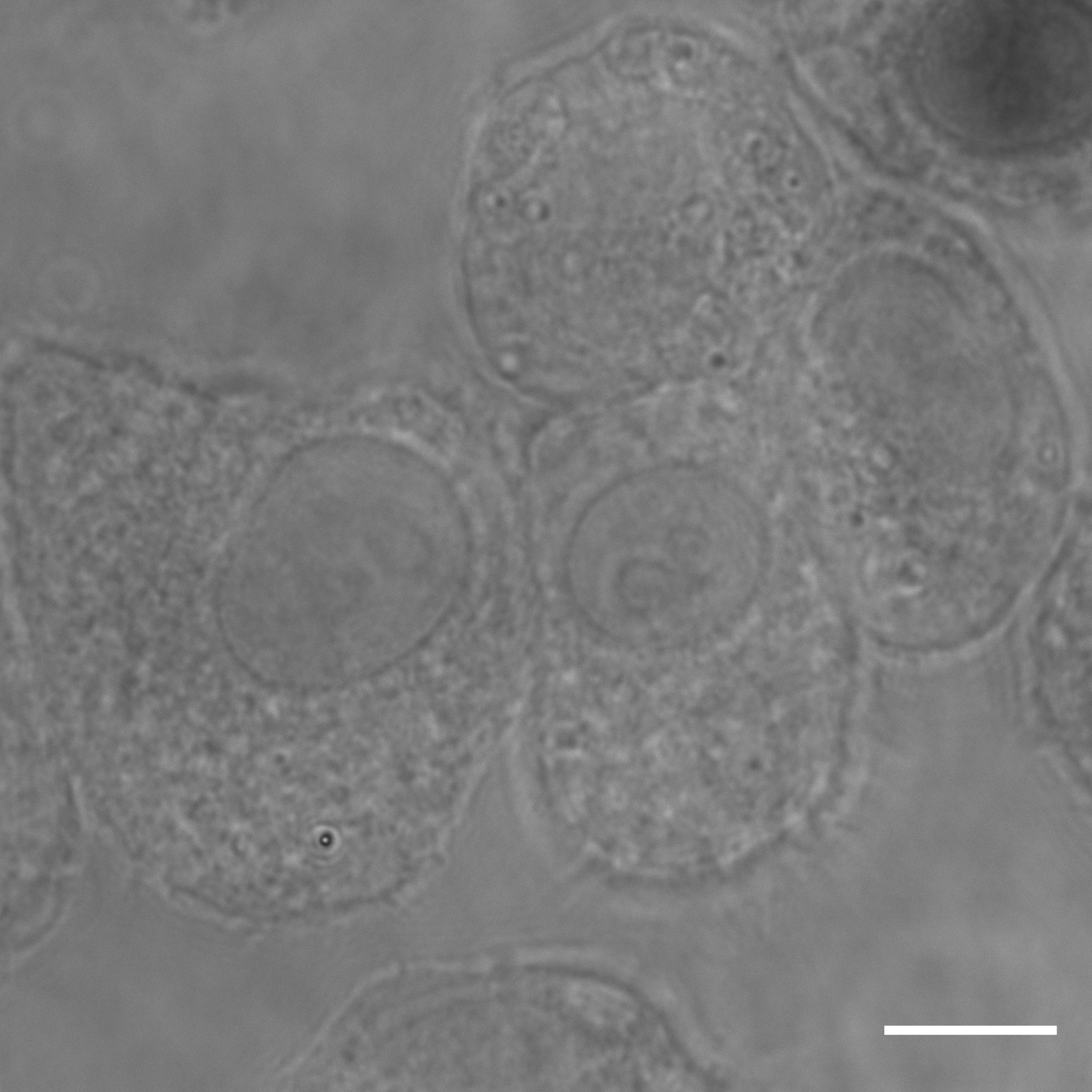

Supplement: Supplementary file 8 — Source data Fig. 3 [file 44319_2025_620_MOESM8_ESM.zip › Figure 3/3B/EMBOR-2025-61666V_RAW_Hb-rexWAGO_T-PMT-T4.tif.tif]

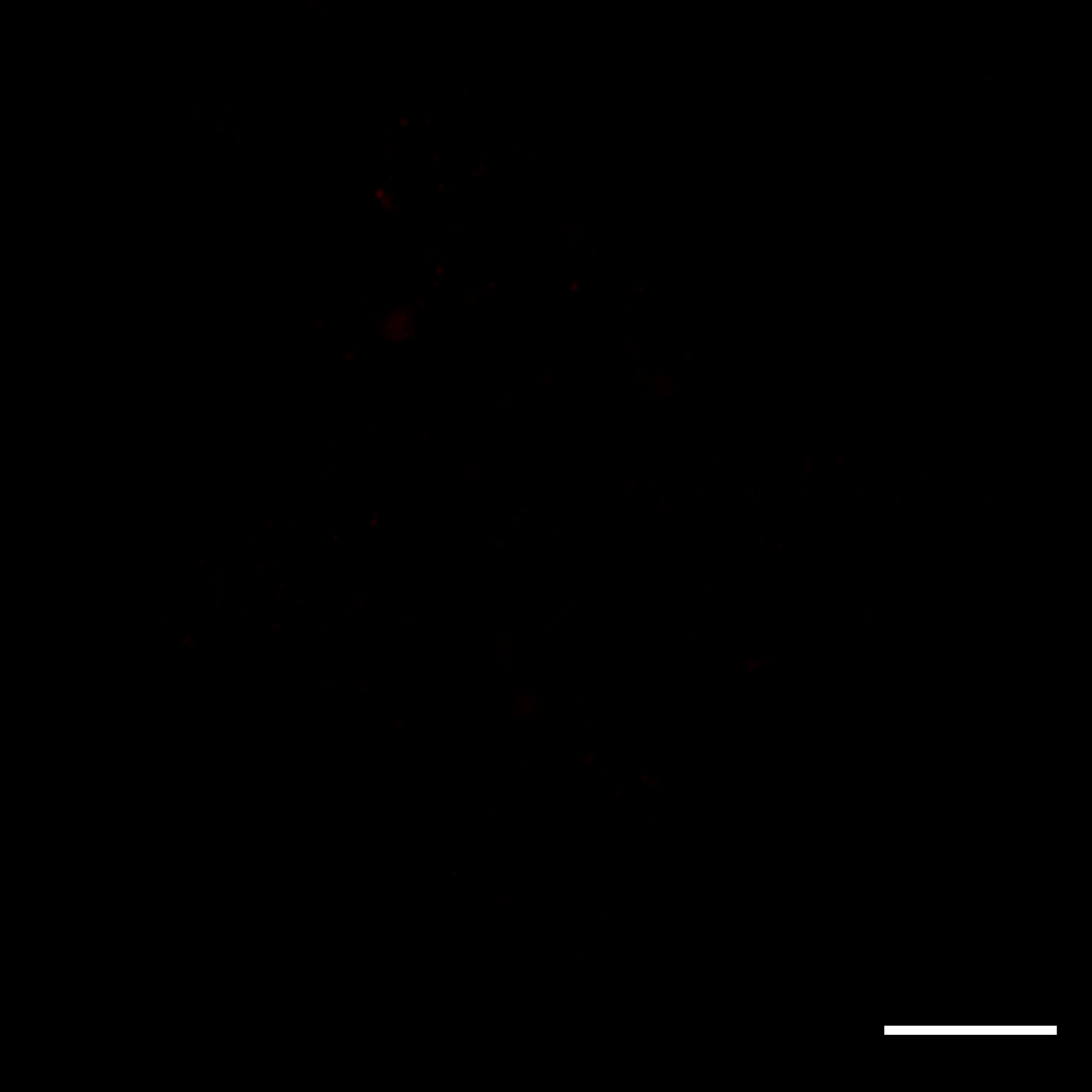

Supplement: Supplementary file 8 — Source data Fig. 3 [file 44319_2025_620_MOESM8_ESM.zip › Figure 3/3B/EMBOR-2025-61666V_RAW_PBS_AF647-T1.tif.tif]

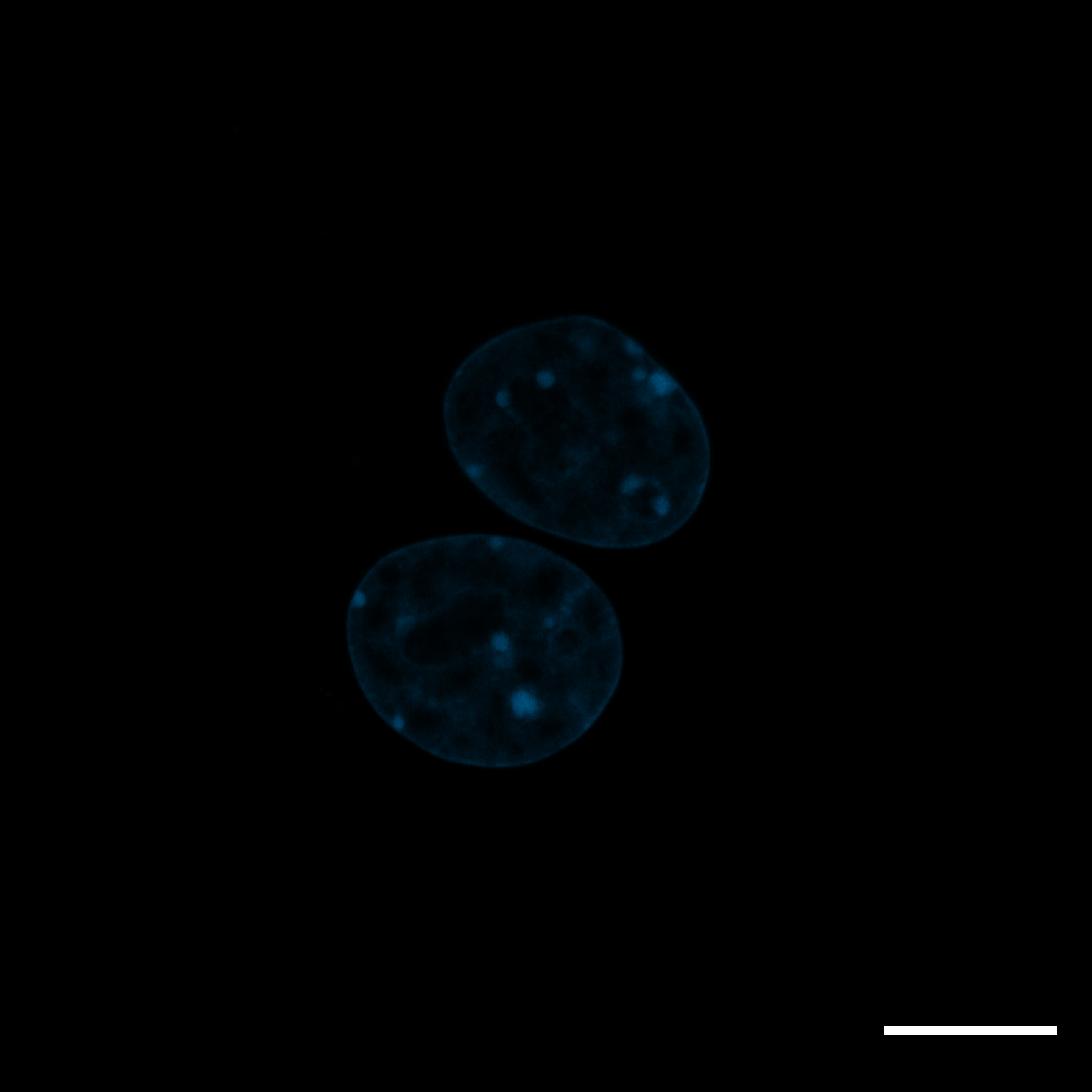

Supplement: Supplementary file 8 — Source data Fig. 3 [file 44319_2025_620_MOESM8_ESM.zip › Figure 3/3B/EMBOR-2025-61666V_RAW_PBS_DAPI-T3.tif.tif]

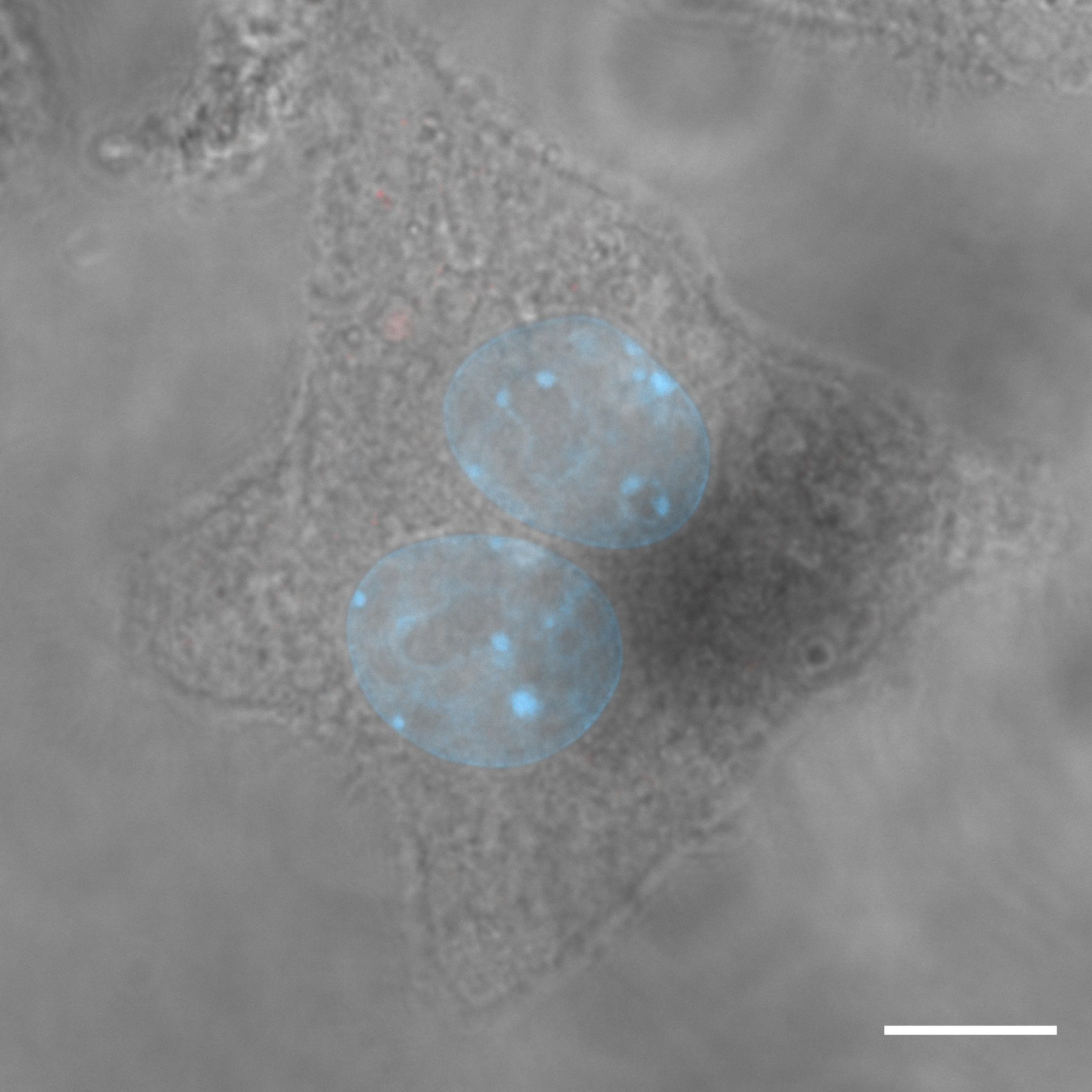

Supplement: Supplementary file 8 — Source data Fig. 3 [file 44319_2025_620_MOESM8_ESM.zip › Figure 3/3B/EMBOR-2025-61666V_RAW_PBS_Merge.tif.tif]

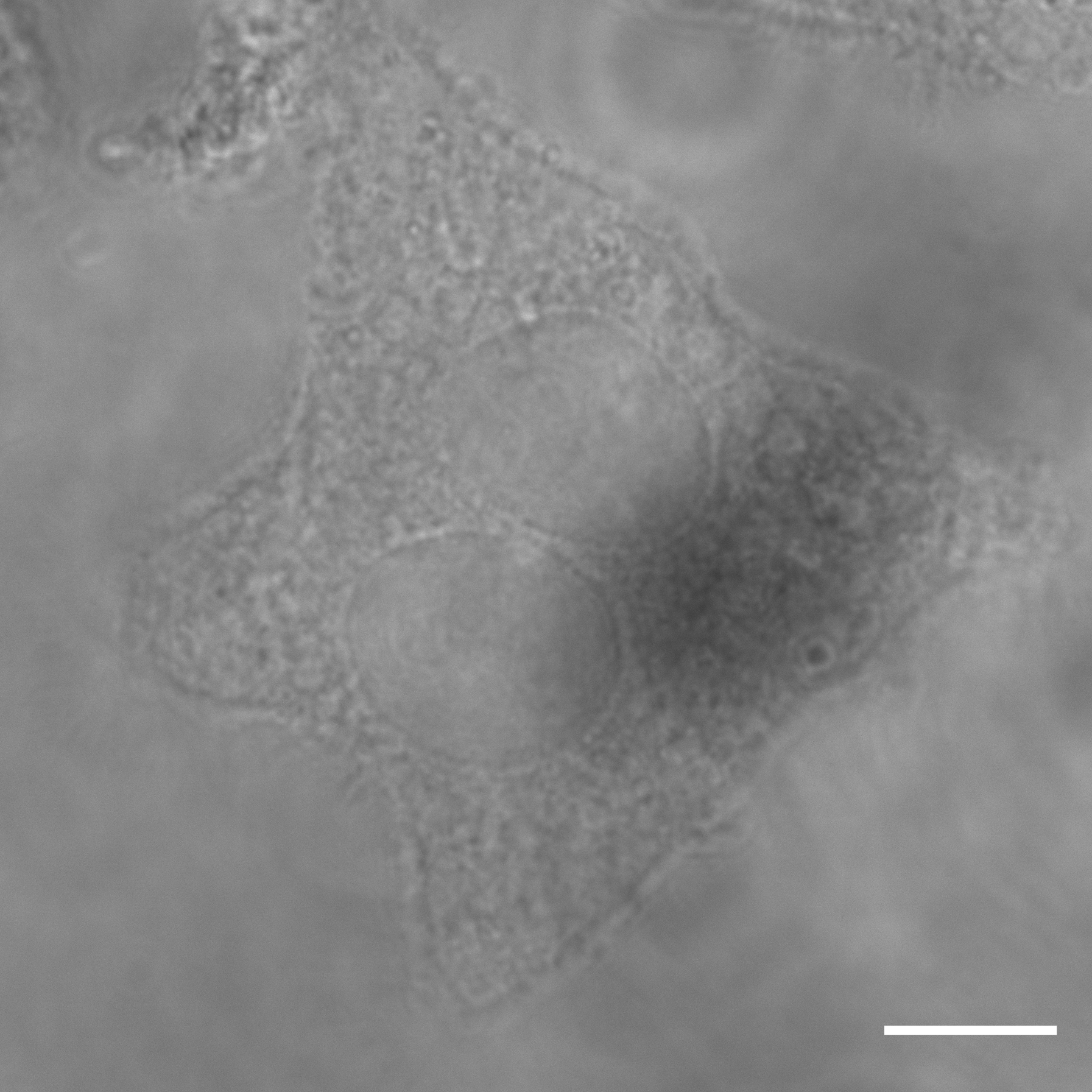

Supplement: Supplementary file 8 — Source data Fig. 3 [file 44319_2025_620_MOESM8_ESM.zip › Figure 3/3B/EMBOR-2025-61666V_RAW_PBS_T-PMT-T4.tif.tif]

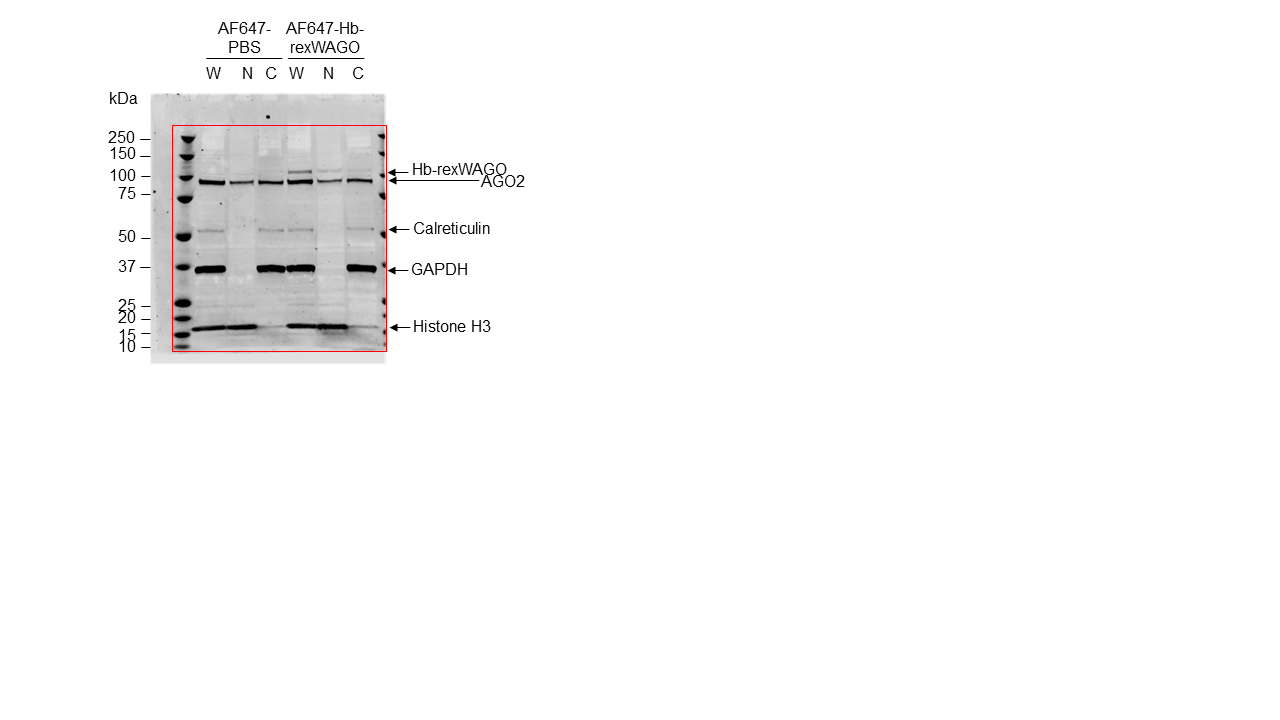

Supplement: Supplementary file 8 — Source data Fig. 3 [file 44319_2025_620_MOESM8_ESM.zip › Figure 3/3D/EMBO-2025-61666V_3D_western.tif.tif]

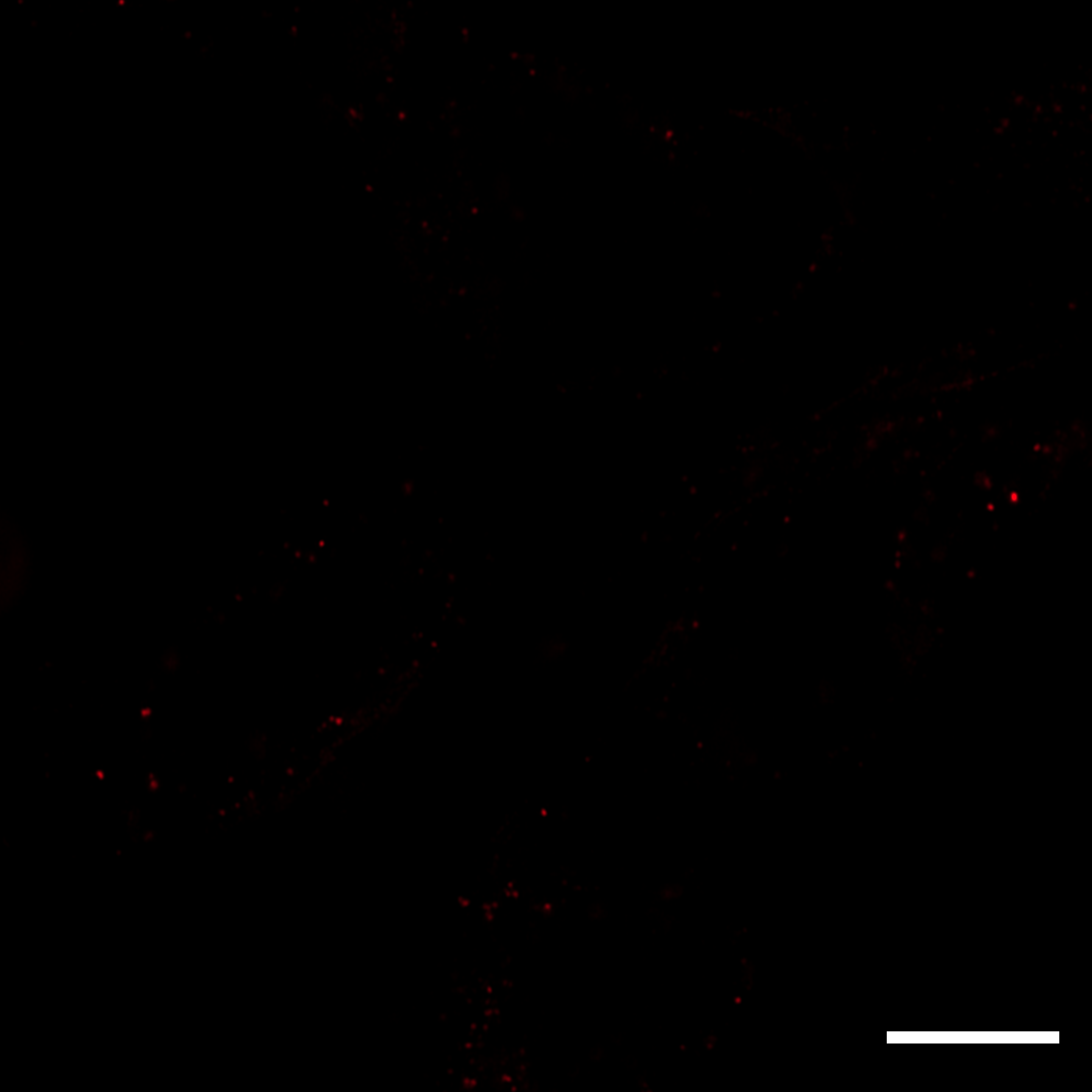

Supplement: Supplementary file 9 — Source data Fig. 4 [file 44319_2025_620_MOESM9_ESM.zip › Figure 4/4F/EMBOR-2025-61666V_anti-Hb-exWAGO serum_AF647.tif.tif]

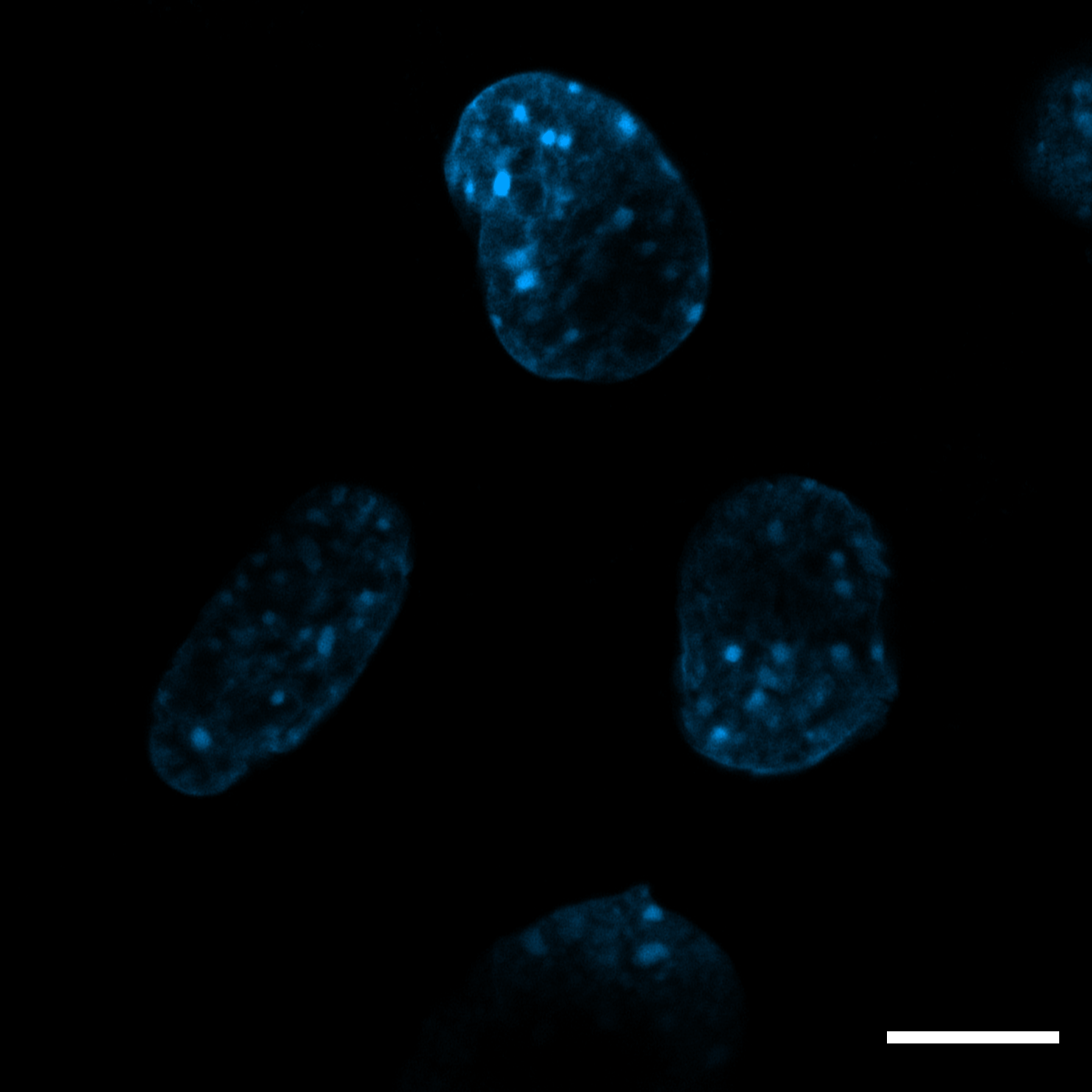

Supplement: Supplementary file 9 — Source data Fig. 4 [file 44319_2025_620_MOESM9_ESM.zip › Figure 4/4F/EMBOR-2025-61666V_anti-Hb-exWAGO serum_DAPI.tif.tif]

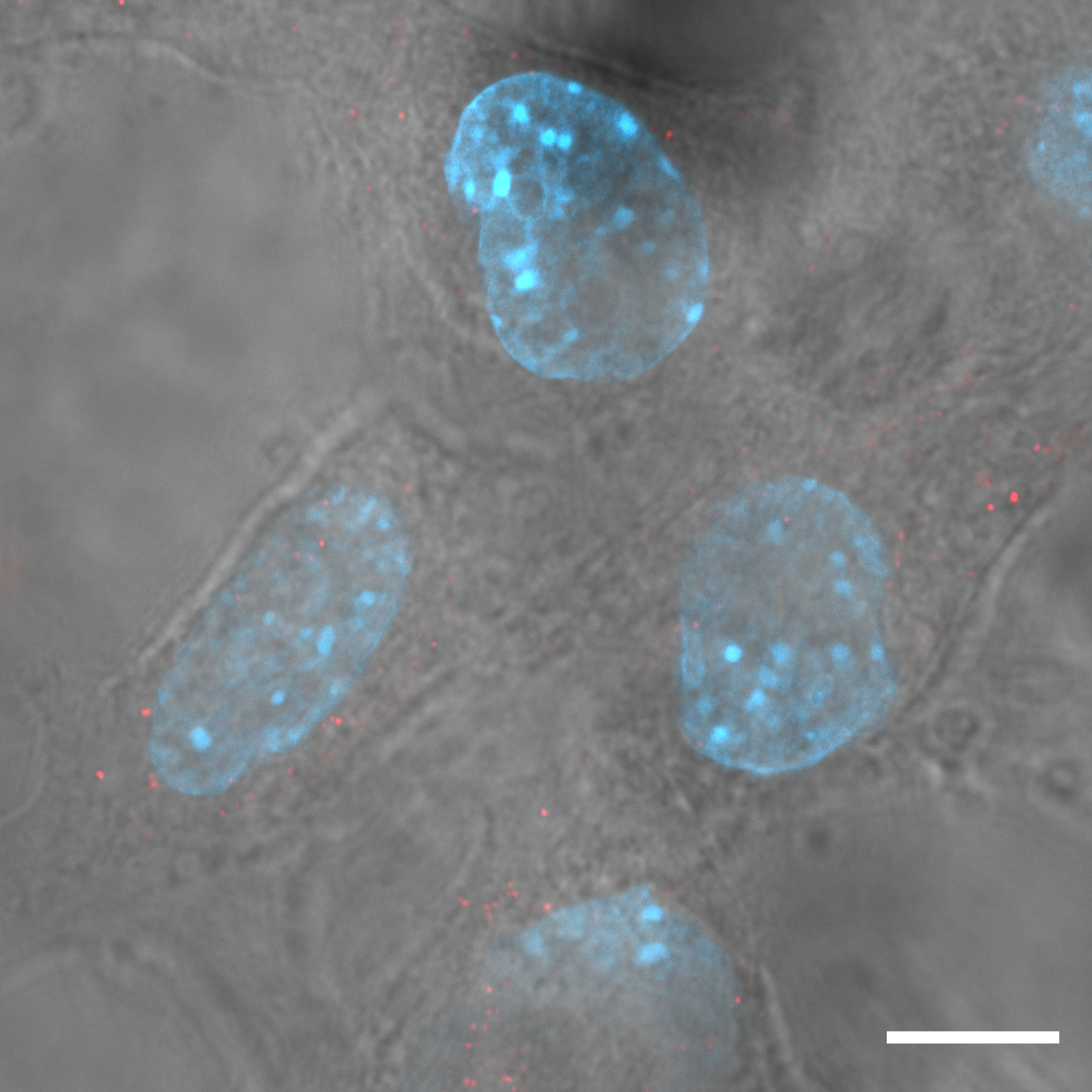

Supplement: Supplementary file 9 — Source data Fig. 4 [file 44319_2025_620_MOESM9_ESM.zip › Figure 4/4F/EMBOR-2025-61666V_anti-Hb-exWAGO serum_Merge.tif.tif]

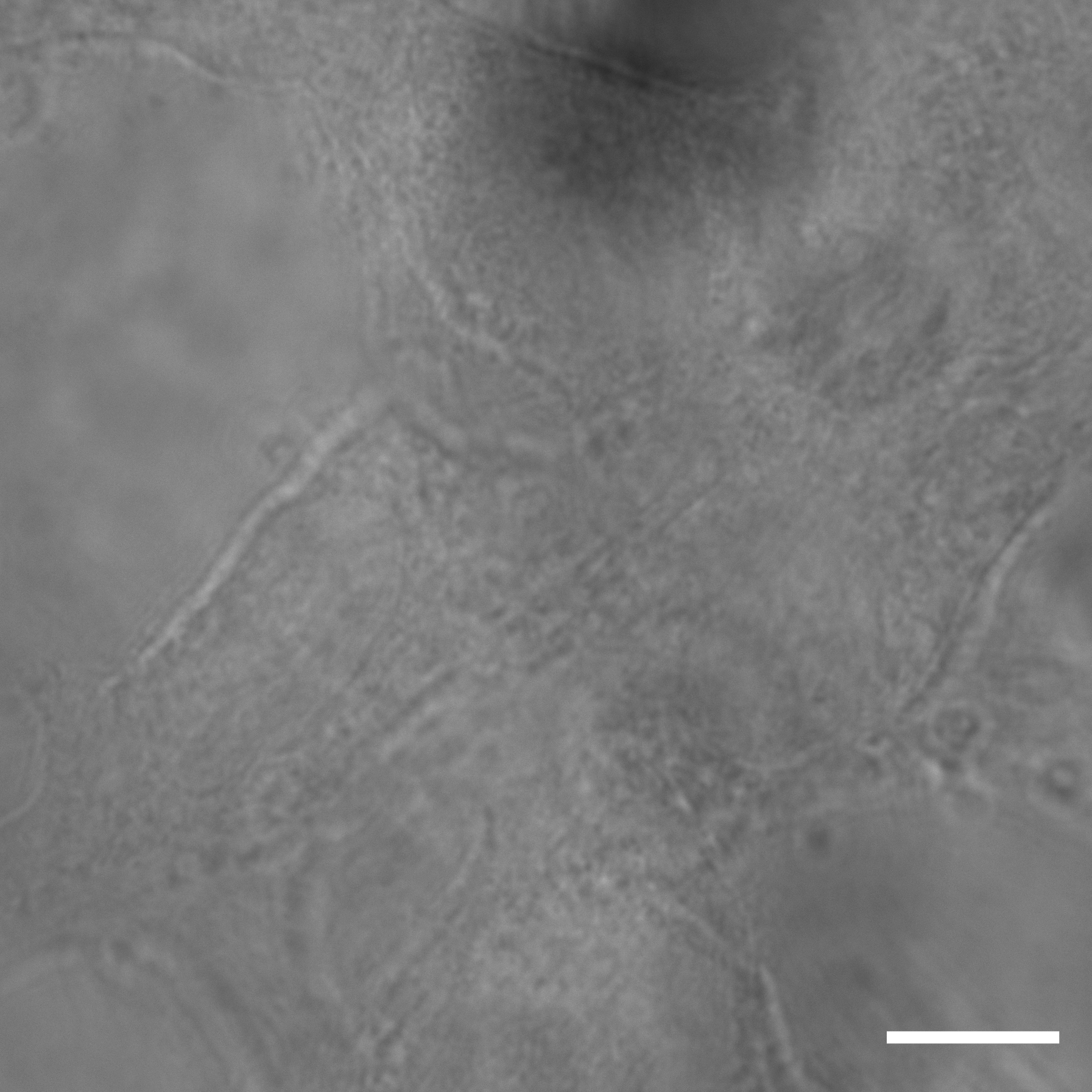

Supplement: Supplementary file 9 — Source data Fig. 4 [file 44319_2025_620_MOESM9_ESM.zip › Figure 4/4F/EMBOR-2025-61666V_anti-Hb-exWAGO serum_Transmitted light.tif.tif]

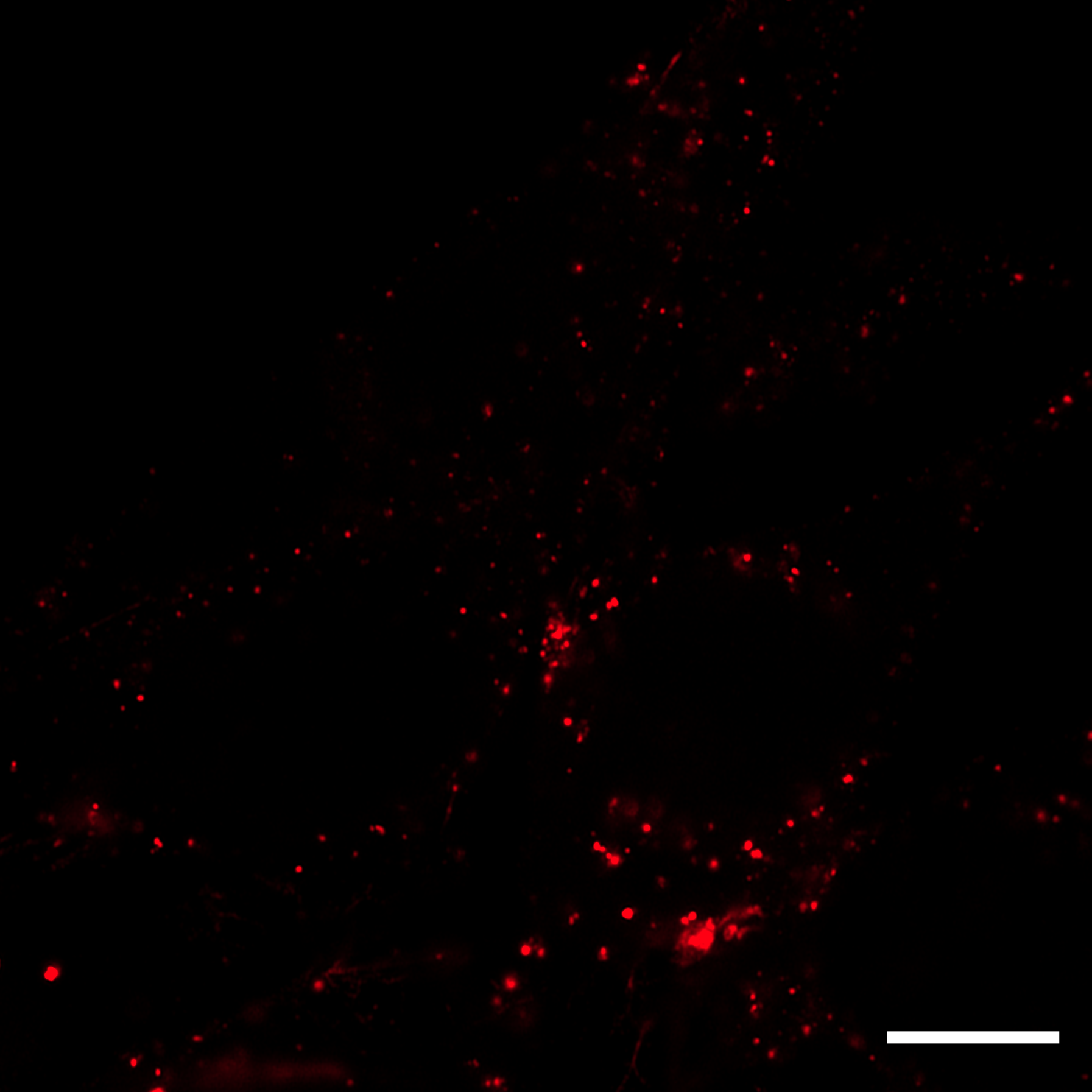

Supplement: Supplementary file 9 — Source data Fig. 4 [file 44319_2025_620_MOESM9_ESM.zip › Figure 4/4F/EMBOR-2025-61666V_naive serum_AF647.tif.tif]

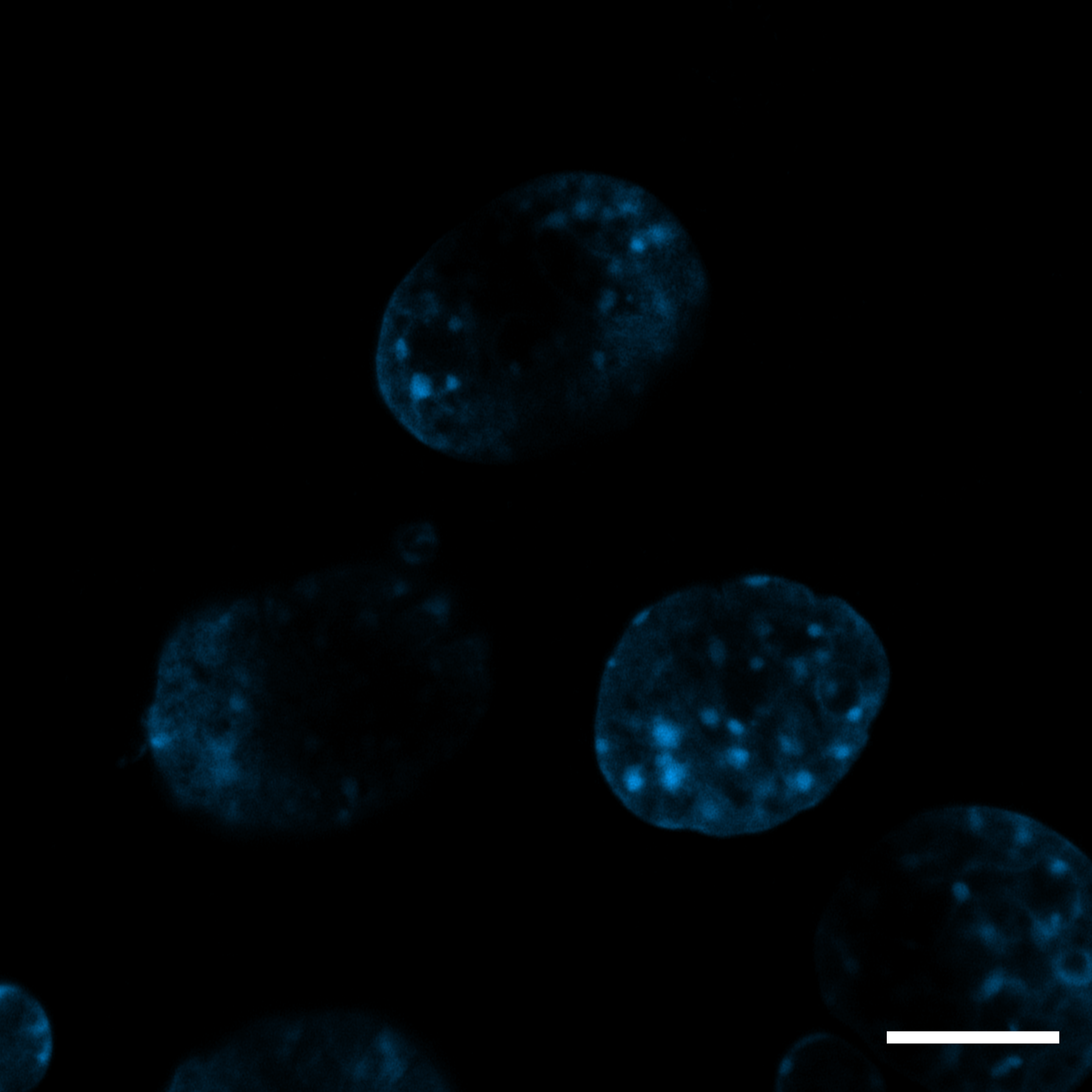

Supplement: Supplementary file 9 — Source data Fig. 4 [file 44319_2025_620_MOESM9_ESM.zip › Figure 4/4F/EMBOR-2025-61666V_naive serum_DAPI.tif.tif]

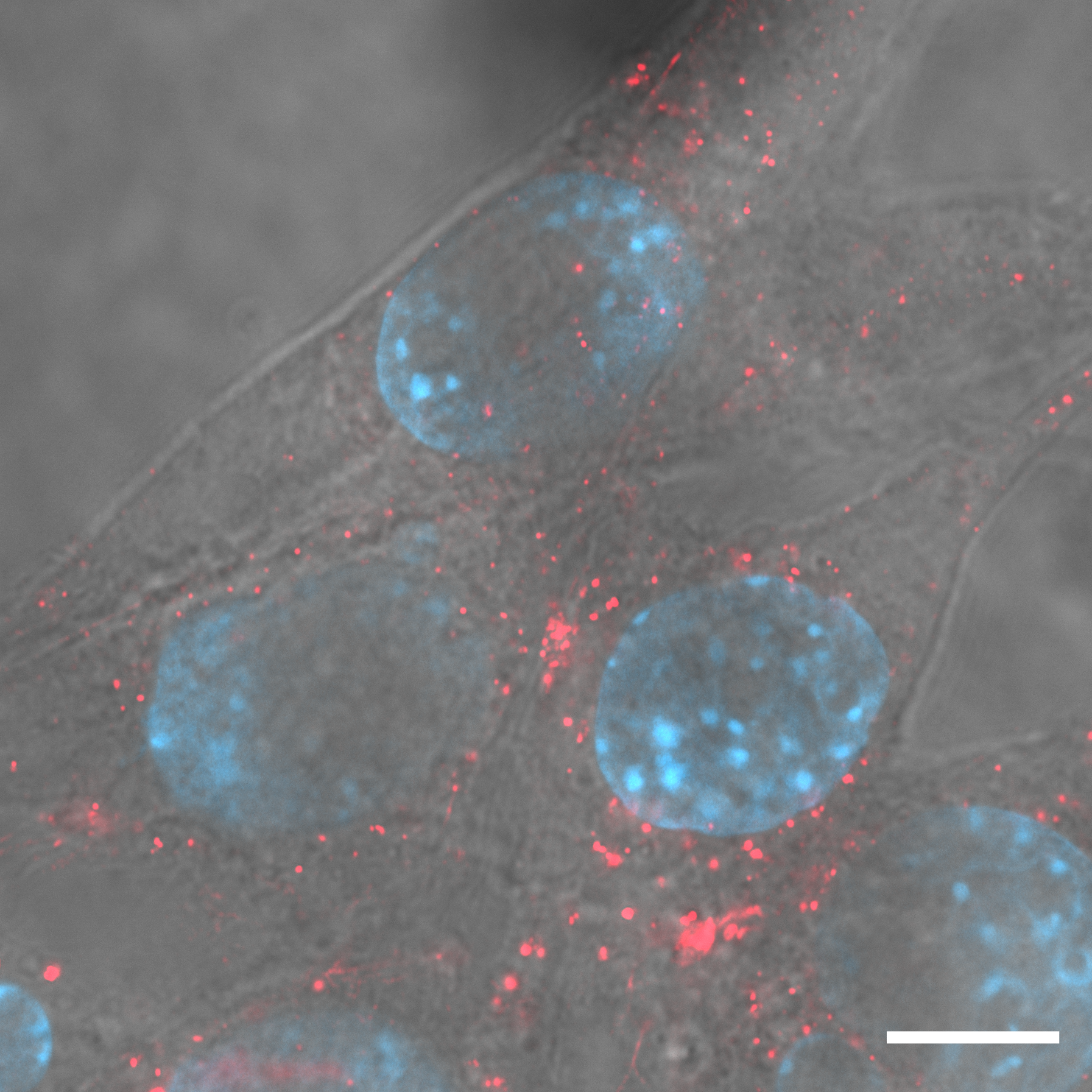

Supplement: Supplementary file 9 — Source data Fig. 4 [file 44319_2025_620_MOESM9_ESM.zip › Figure 4/4F/EMBOR-2025-61666V_naive serum_Merge.tif.tif]

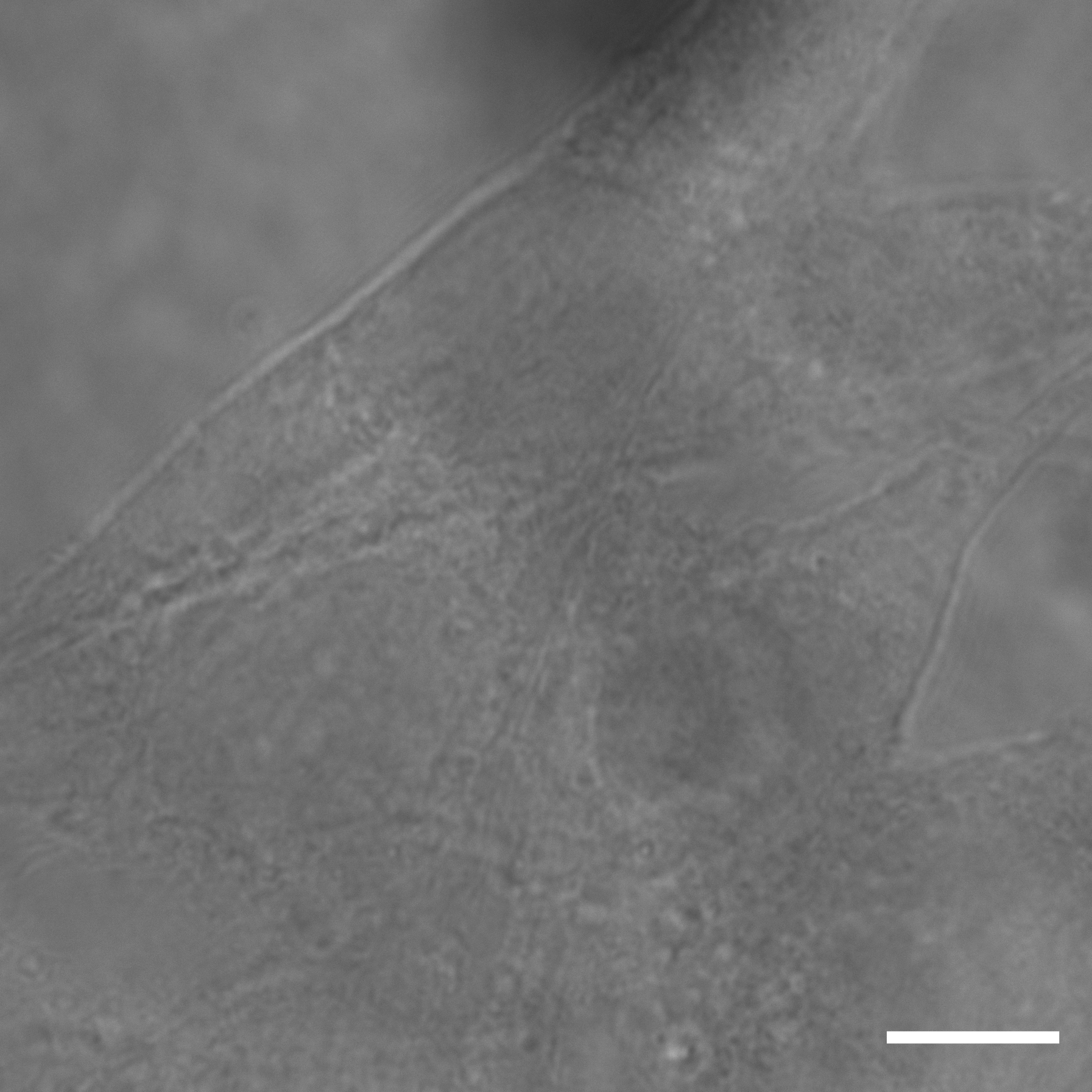

Supplement: Supplementary file 9 — Source data Fig. 4 [file 44319_2025_620_MOESM9_ESM.zip › Figure 4/4F/EMBOR-2025-61666V_naive serum_Transmitted light.tif.tif]

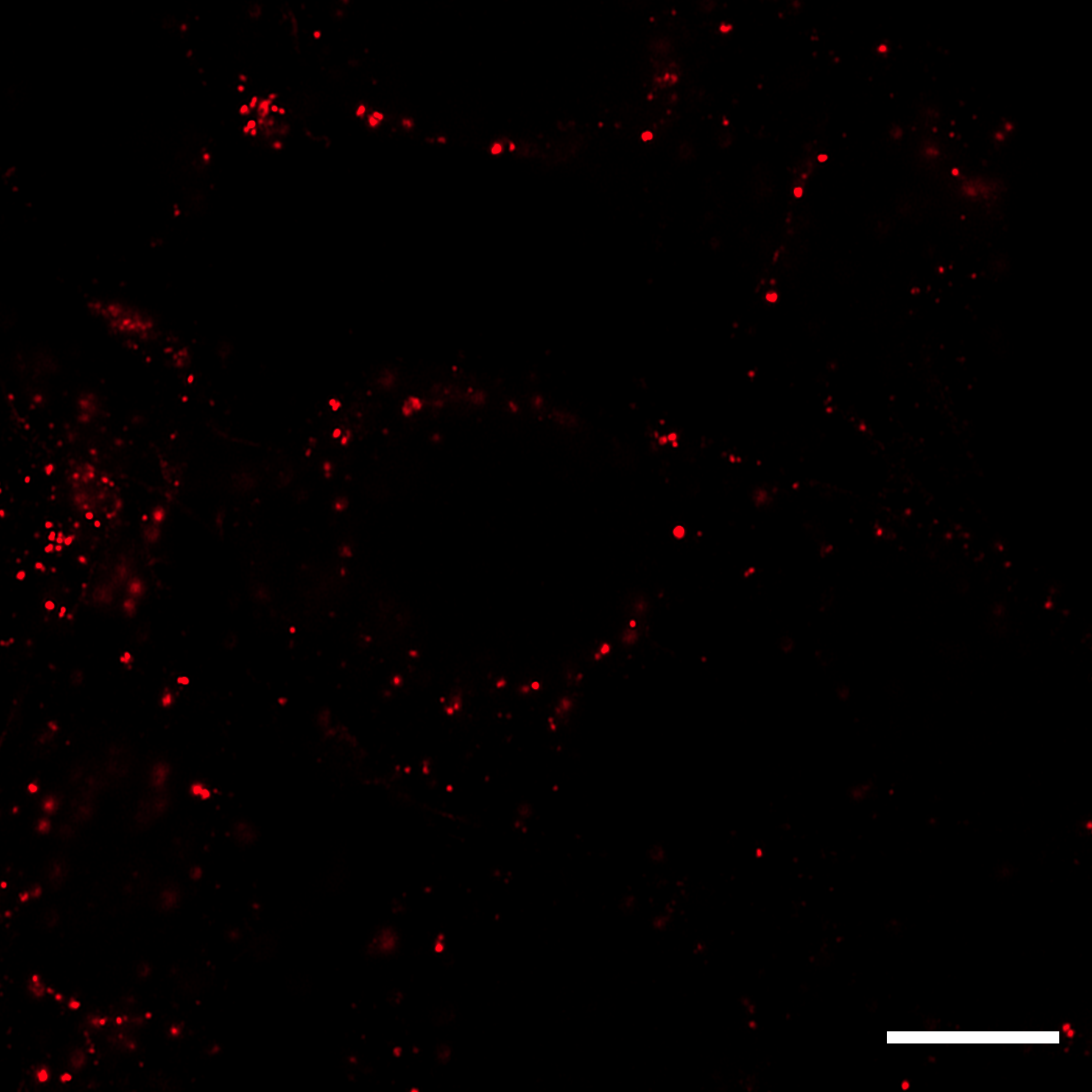

Supplement: Supplementary file 9 — Source data Fig. 4 [file 44319_2025_620_MOESM9_ESM.zip › Figure 4/4F/EMBOR-2025-61666V_no ab_AF647.tif.tif]

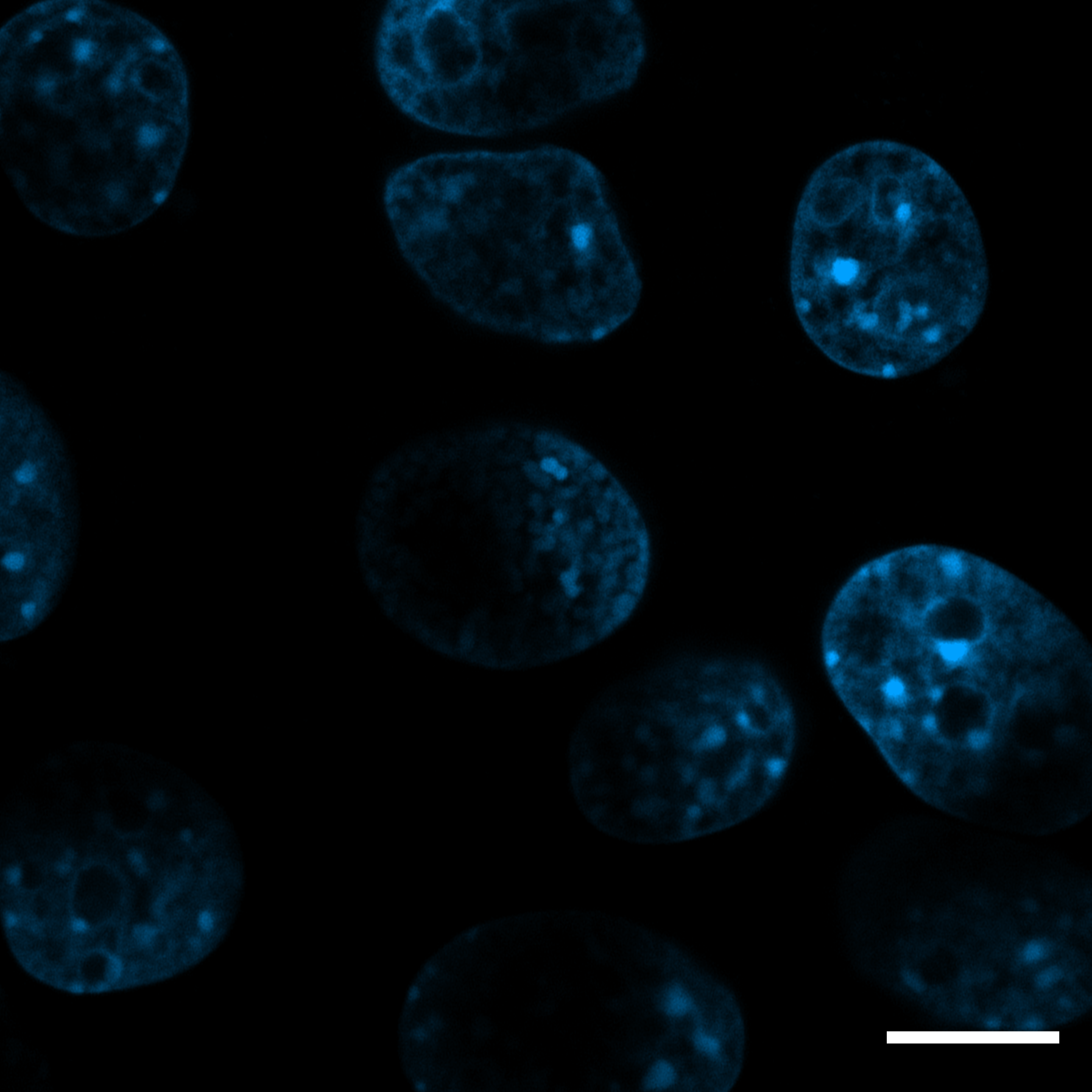

Supplement: Supplementary file 9 — Source data Fig. 4 [file 44319_2025_620_MOESM9_ESM.zip › Figure 4/4F/EMBOR-2025-61666V_no ab_DAPI.tif.tif]

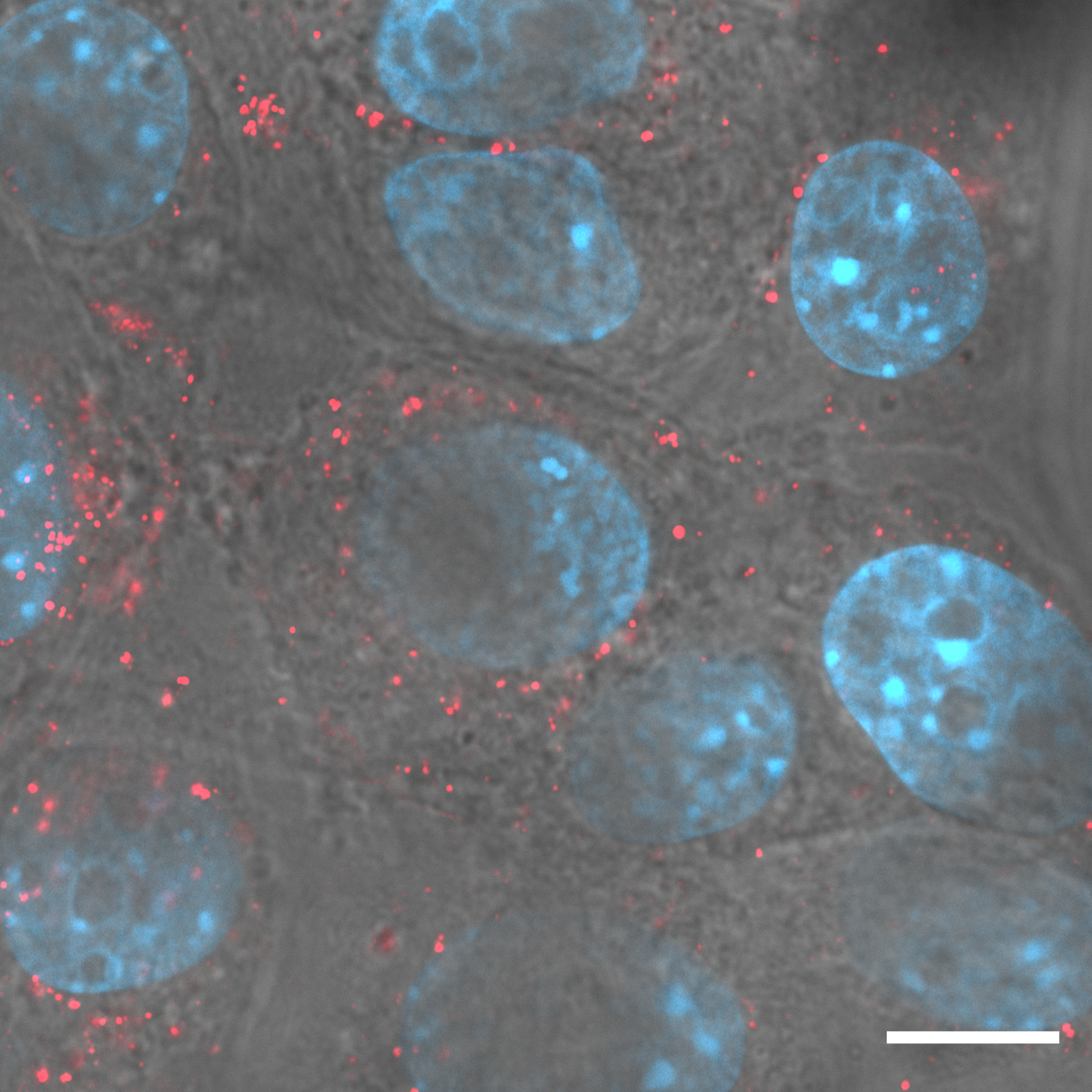

Supplement: Supplementary file 9 — Source data Fig. 4 [file 44319_2025_620_MOESM9_ESM.zip › Figure 4/4F/EMBOR-2025-61666V_no ab_Merge.tif.tif]

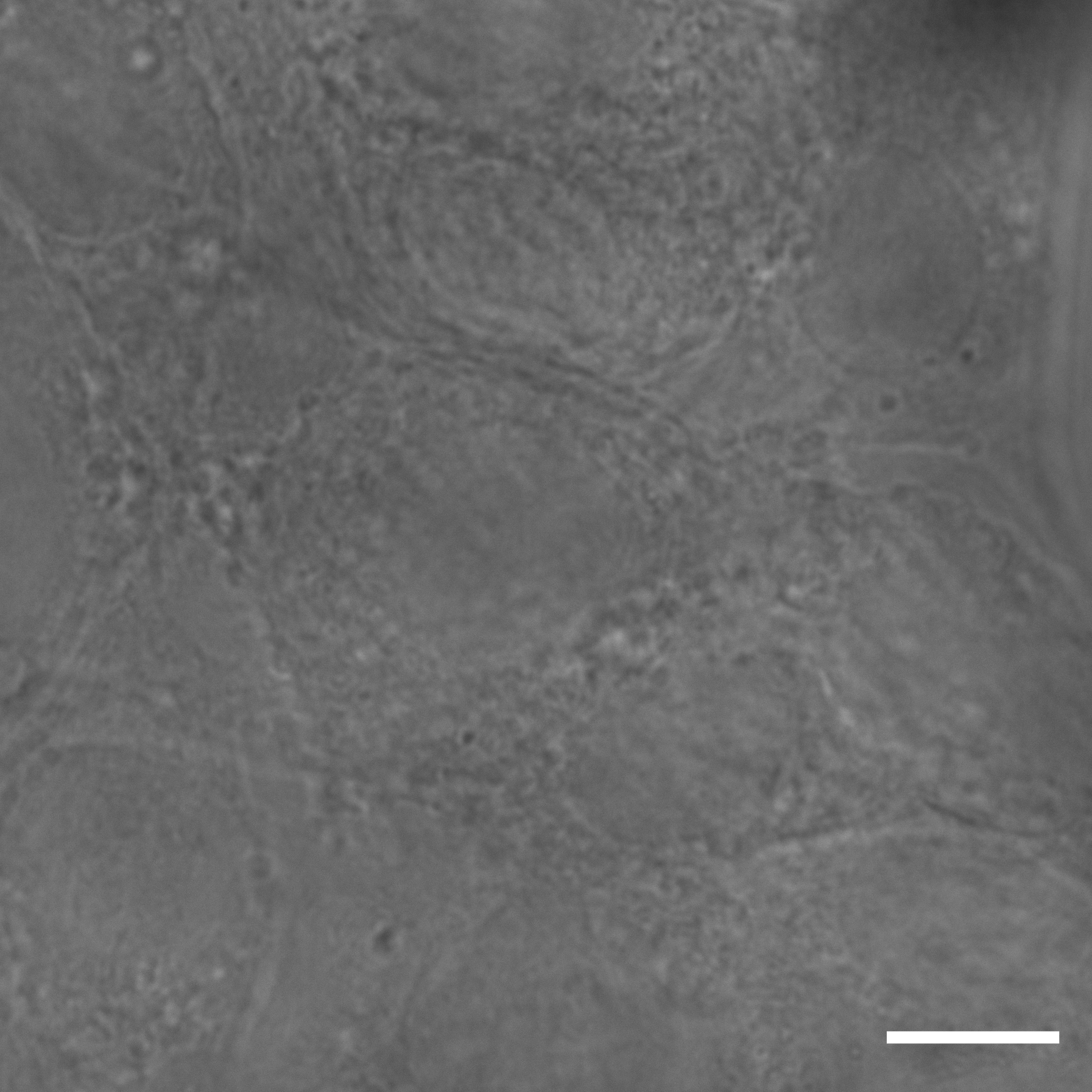

Supplement: Supplementary file 9 — Source data Fig. 4 [file 44319_2025_620_MOESM9_ESM.zip › Figure 4/4F/EMBOR-2025-61666V_no ab_Transmitted light.tif.tif]

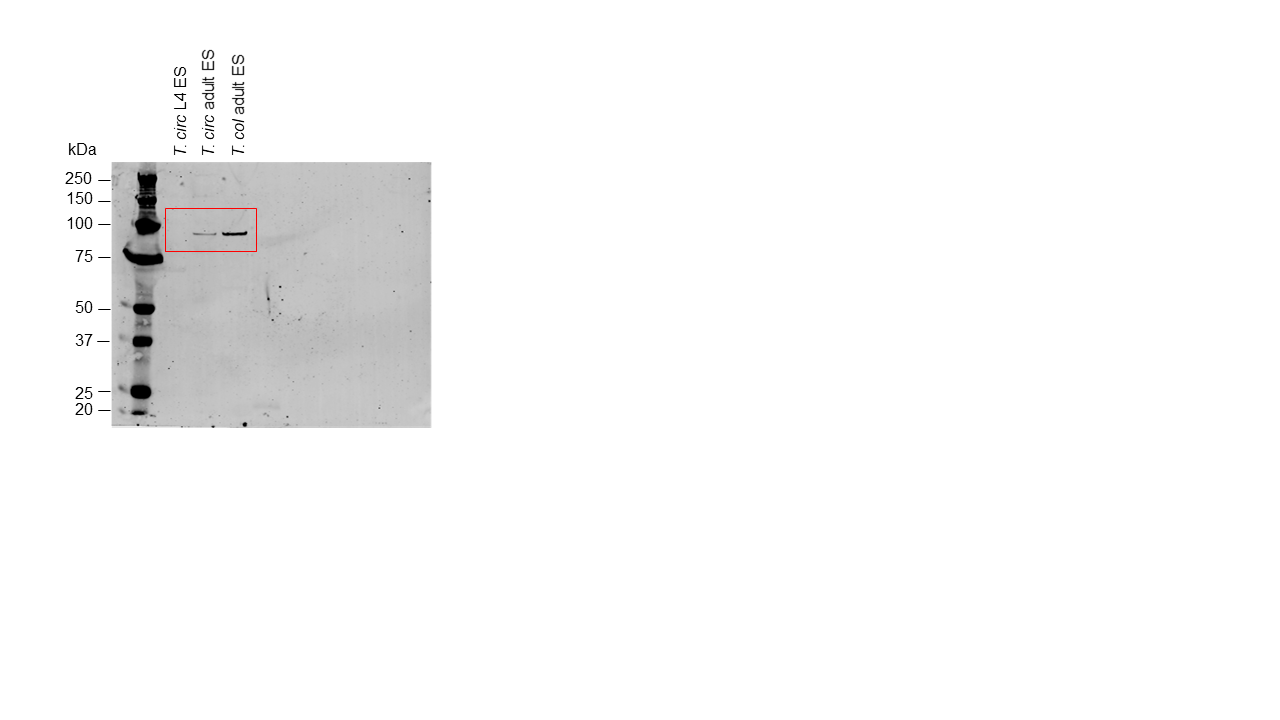

Supplement: Supplementary file 10 — Source data Fig. 5 [file 44319_2025_620_MOESM10_ESM.zip › Figure 5/5C/EMBOR-2025-61666V_5C-western.tif.tif]

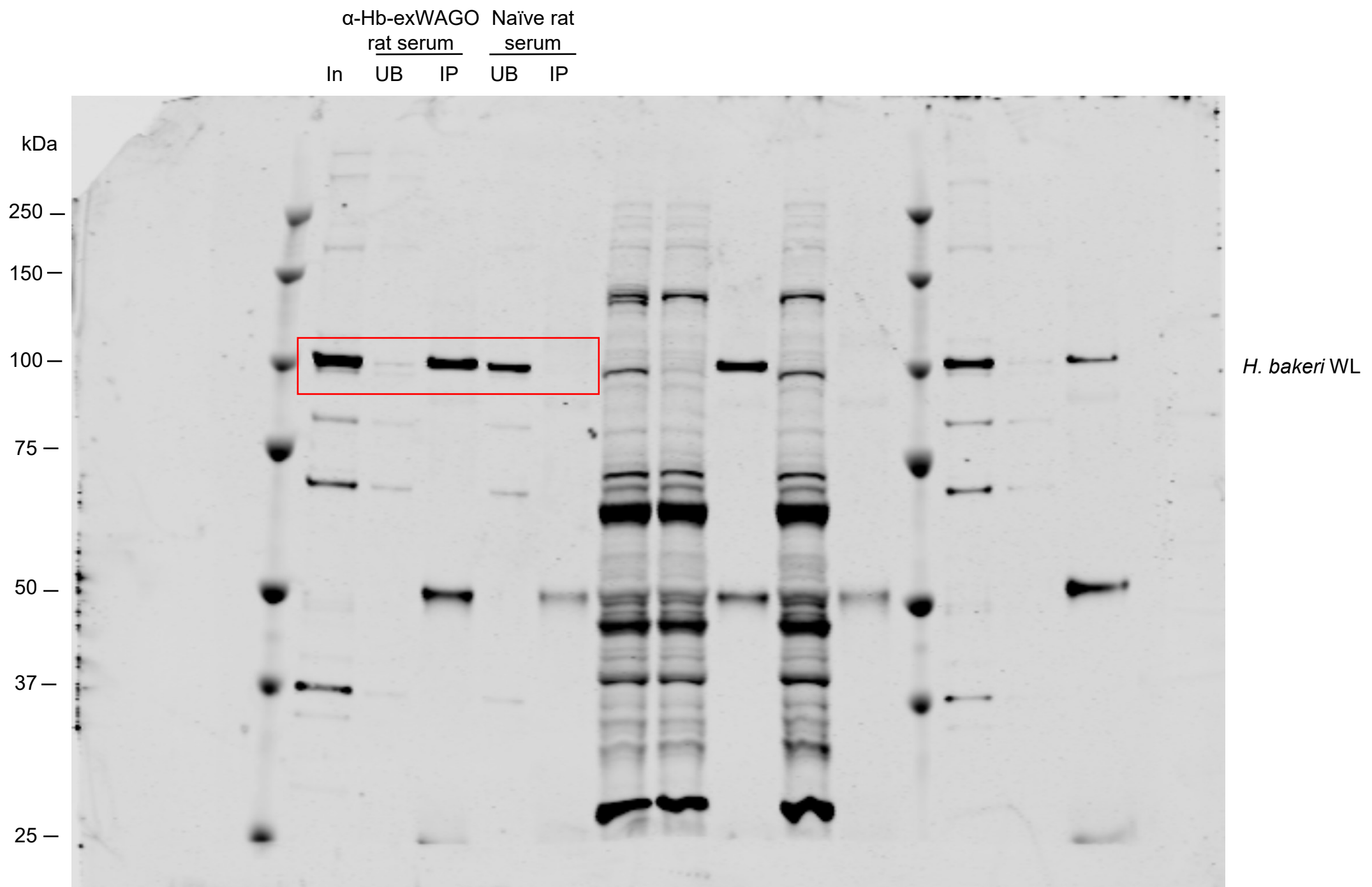

$\alpha$ -Hb-exWAGO    Naïve rat  
    rat serum      serum  
In    UB    IP    UB    IP

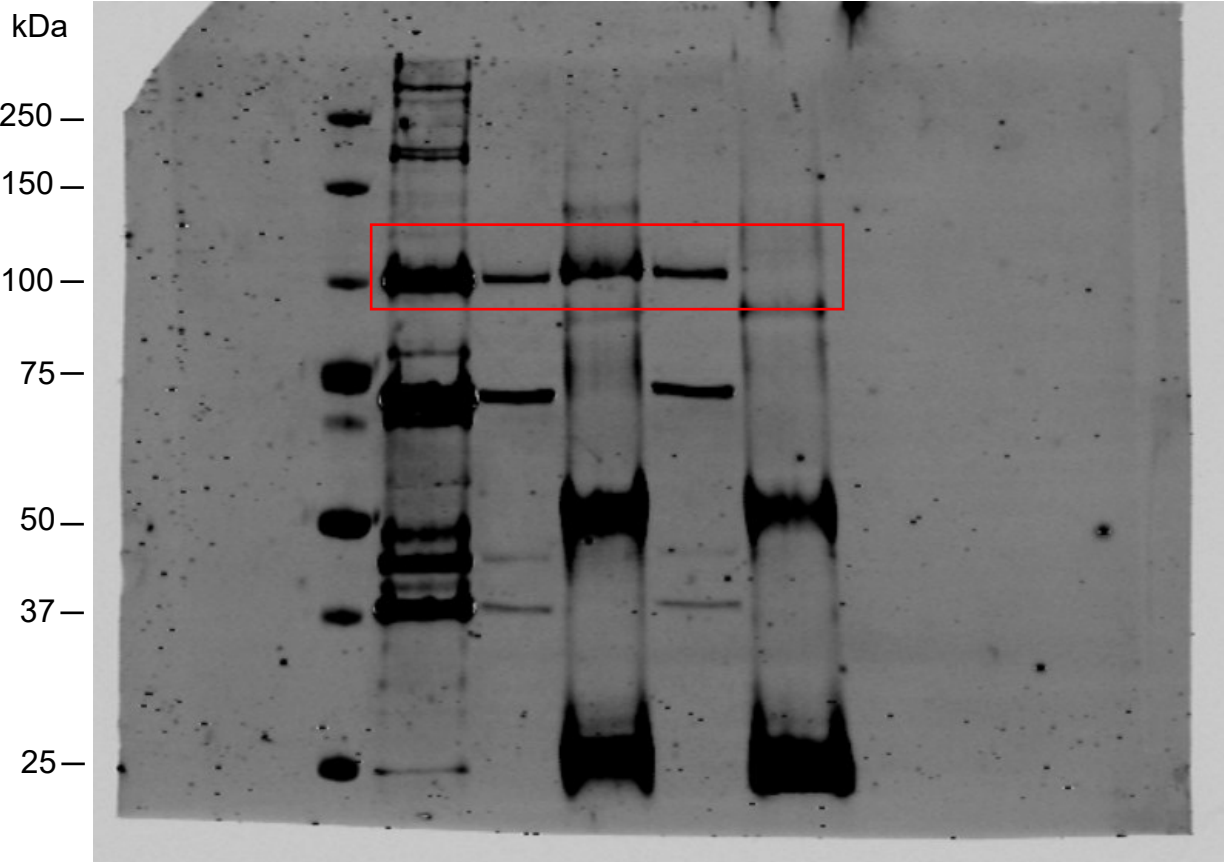

*N. brasiliensis* WL

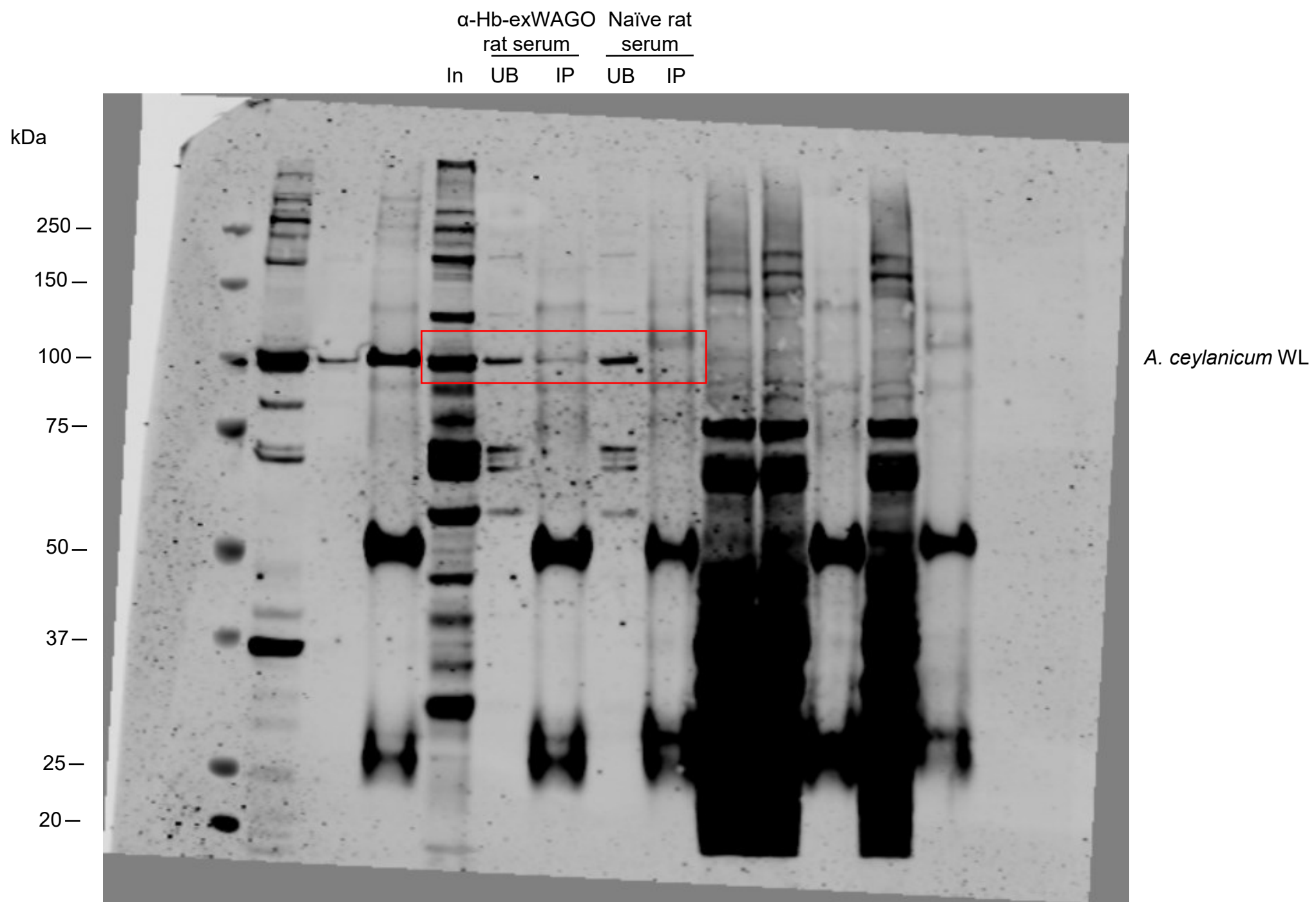

Supplement: Supplementary file 10 — Source data Fig. 5 [file 44319_2025_620_MOESM10_ESM.zip › Figure 5/5D/EMBOR-2025-61666V_5D-western.pdf.pdf]

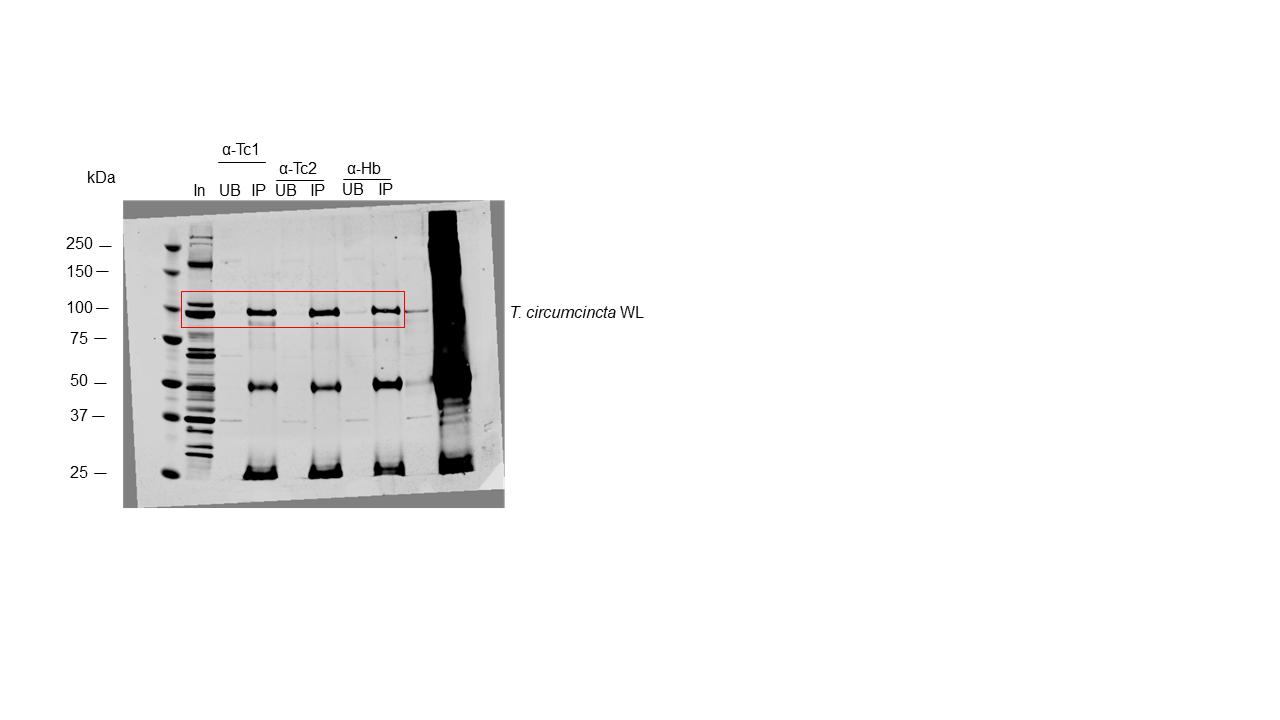

Supplement: Supplementary file 10 — Source data Fig. 5 [file 44319_2025_620_MOESM10_ESM.zip › Figure 5/5E/EMBOR-2025-61666V_5E-western.tif]
