# Supplementary material for: An Argonaute protein traffics from nematode to mouse and is a vaccine against parasitic nematodes
Source: EMBO Rep. 2025 Dec 9;27(2):311–40. doi: 10.1038/s44319-025-00620-4 (PMC12852730; doi:10.1038/s44319-025-00620-4)
Supplement: Supplementary file 11 — Expanded View Figures [file 44319_2025_620_MOESM11_ESM.pdf]

Expanded View Figures

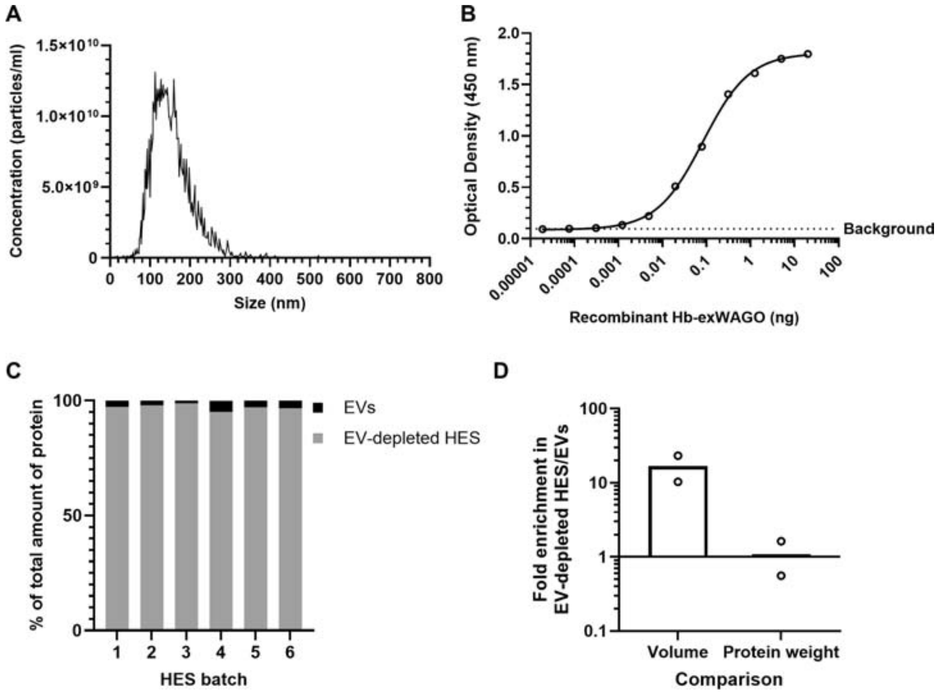

Figure EV1. Quantification of exWAGO in EVs and EV-depleted HES.

(**A**) Representative particle size distribution profile and quantification of *H. bakeri* EVs following ultracentrifugation. Data show the mean of $n = 3$ technical replicates. (**B**) Representative standard curve of Hb-exWAGO concentration based on ELISA using the recombinant Hb-exWAGO analysed using 4PL regression. Data show the mean of $n = 2$ technical replicates. (**C**) The amount of total protein quantified in EV-depleted HES and EVs following separation of HES by ultracentrifugation, shown as percentage of total protein of both fractions combined. Data are from biologically independent batches of HES collected from day 1–8 post-harvest of worms from mice ($n = 6$ biological replicates). (**D**) Fold-enrichment of Hb-exWAGO in EV-depleted HES compared to EVs following quantification of western blot band intensities. EV-depleted HES and EVs from the same starting material were analysed based on loading from equivalent starting sample volumes (left) or based on loading equivalent total protein (2.0 µg). Individual data points are shown with the mean ($n = 2$ biological replicates).

 © The Author(s)

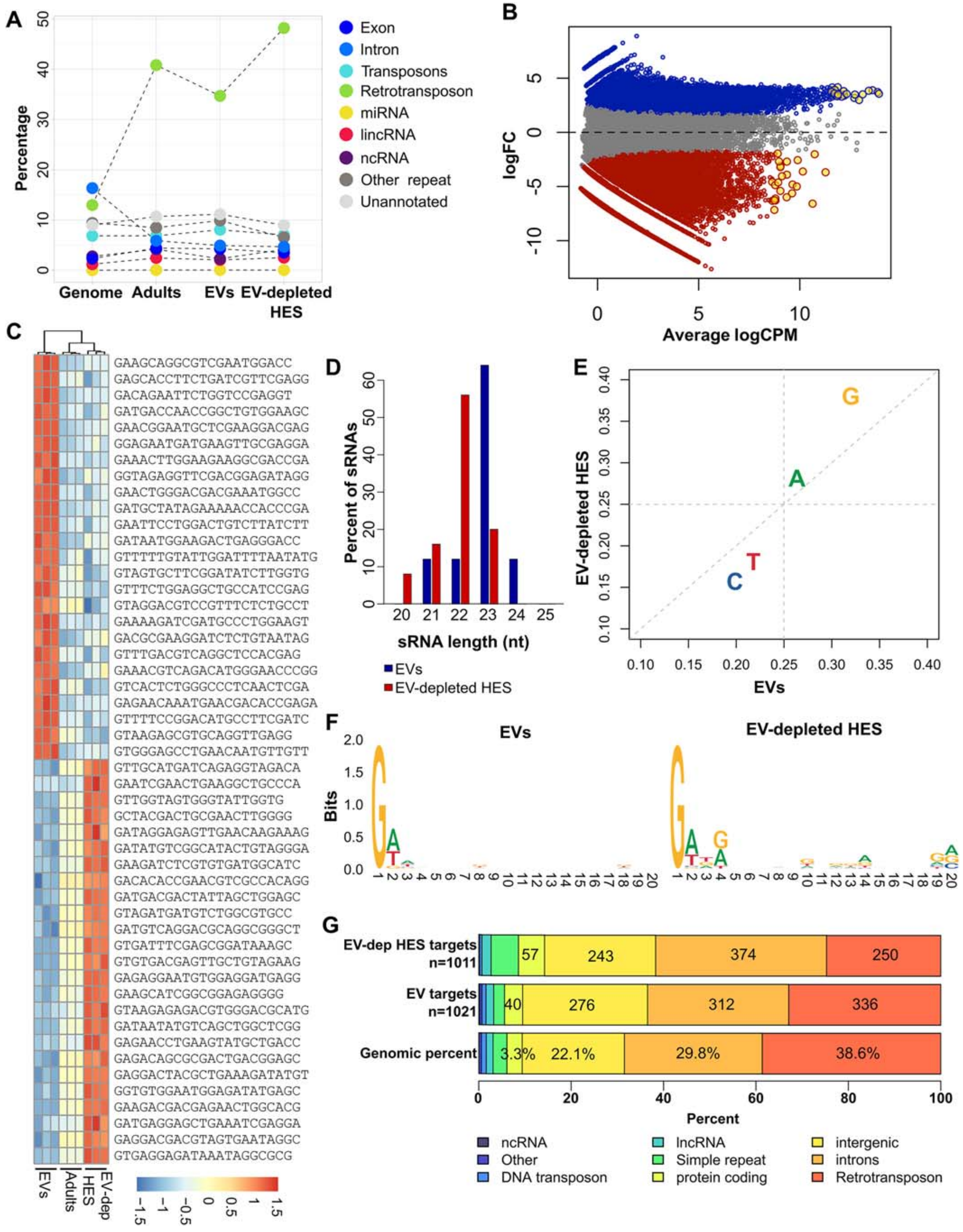
A
Percentage
0
10
20
30
40
50
Genome
Adults
EVs
EV-depleted HES
Exon
Intron
Transposons
Retrotransposon
miRNA
lincRNA
ncRNA
Other repeat
Unannotated
B
logFC
5
0
-5
-10
0
5
10
Average logCPM
C
GAAGCAGGCGTCGAATGGACC
GAGCACCTTCTGATCGTTCGAGG
GACAGAATTCTGGTCCGAGGT
GATGACCAACCGGCTGTGGAAGC
GAACGGAATGCTCGAAGGACGAG
GGAGAATGATGAAGTTGCGAGGA
GAAACTTGGAAGAAGGCGACCGA
GGTAGAGGTTCGACGGAGATAGG
GAACTGGGACGACGAAATGGCC
GATGCTATAGAAAAACCACCCGA
GAATTCCTGGACTGTCTTATCTT
GATAATGGAAGACTGAGGGACC
GTTTTTGTATTGGATTTTAATATG
GTAGTGCTTCGGATATCTTGGTG
GTTTCTGGAGGCTGCCATCCGAG
GTAGGACGTCCGTTTCTCTGCCT
GAAAAGATCGATGCCCTGGAAGT
GACGCGAAGGATCTCTGTAATAG
GTTTGACGTCAGGCTCCACGAG
GAAACGTCAGACATGGGAACCCGG
GTCACTCTGGGCCCTCAACTCGA
GAGAACAAATGAACGACACCGAGA
GTTTTCCGGACATGCCTTCGATC
GTAAGAGCGTGCAGGTTGAGG
GTGGGAGCCTGAACAATGTTGTT
GTTGCATGATCAGAGGTAGACA
GAATCGAACTGAAGGCTGCCCA
GTTGGTAGTGGGTATTGGTG
GCTACGACTGCGAACTTGGGG
GATAGGAGAGTTGAACAAGAAAG
GATATGTCGGCATACTGTAGGGA
GAAGATCTCGTGTGATGGCATC
GACACACCGAACGTCGCCACAGG
GATGACGACTATTAGCTGGAGC
GTAGATGATGTCTGGCGTGCC
GATGTCAGGACGCAGGCGGGCT
GTGATTTCGAGCGGATAAAGC
GTGTGACGAGTTGCTGTAGAAG
GAGAGGAATGTGGAGGATGAGG
GAAGCATCGGCGGAGAGGGG
GTAAGAGAGACGTGGGACGCATG
GATAATATGTCAGCTGGCTCGG
GAGAACCTGAAGTATGCTGACC
GAGACAGCGCGACTGACGGAGC
GAGGACTACGCTGAAAGATATGT
GGTGTGGAATGGAGATATGAGC
GAAGACGACGAGAACTGGCACG
GATGAGGAGCTGAAATCGAGGA
GAGGACGACGTAGTGAATAGGC
GTGAGGAGATAAATAGGCGCG
EVs
Adults
EV-dep HES
−1.5
−1
−0.5
0
0.5
1
1.5
D
Percent of sRNAs
0
10
20
30
40
50
60
20
21
22
23
24
25
sRNA length (nt)
EVs
EV-depleted HES
E
EV-depleted HES
EVs
0.10
0.15
0.20
0.25
0.30
0.35
0.40
G
A
T
C
F
EVs
EV-depleted HES
Bits
0.0
0.5
1.0
1.5
2.0
G
EV-dep HES targets n=1011
57
243
374
250
EV targets n=1021
40
276
312
336
Genomic percent
3.3%
22.1%
29.8%
38.6%
0
20
40
60
80
100
Percent
ncRNA
Other
DNA transposon
lncRNA
Simple repeat
protein coding
intergenic
introns
Retrotransposon

Figure EV2. In-depth analysis of sRNAs bound to non-vesicular and vesicular exWAGO.

(**A**) Average percentages of mapped reads assigned to each annotation category of the *H. bakeri* genome for adult, EV and EV-dep HES libraries. The first column shows the percentage of each annotation category in the genome, as a reference. (**B**) An MA plot representing the differential expression between vesicular and non-vesicular Hb-exWAGO forms. The *x* axis shows the average $\log_2$(CPM) and the *y* axis the $\log_2$(Fold-Change). Each dot represents an annotated region of the *H. bakeri* genome. The 10,778 regions with fold-changes significantly higher than 2 in the vesicular libraries are shown in dark blue; the 39,776 regions with fold-changes significantly higher than 2 in non-vesicular libraries are shown in dark red, all controlled for False-Discovery Rate < 1%; the 25 regions with highest CPM for each are highlighted in gold. (**C**) Heat map showing relative abundance (row-scaled Z-scores) for the sRNAs with the highest CPM from the 25 EV and 25 EV-dep HES regions highlighted in (**B**). (**D**) Length distribution, (**E**) total nucleotide frequency and (**F**) nucleotide preference across the first 20nt of the sRNAs shown in (**C**). (**G**) TargetFinder results using the first 20nt of the same sRNAs and the complete mouse genome, grouped according to annotation in the mouse genome of the predicted target sites. Genomic percent of the mouse genome is provided in the first column as a reference. There were 1021 target sites with penalty score <= 3 for the top 25 EV sRNAs, and 1011 for the top 25 EV-dep HES sRNAs. Data information: All figures use the data from the same $n = 3$ biological replicates.

 © The Author(s)

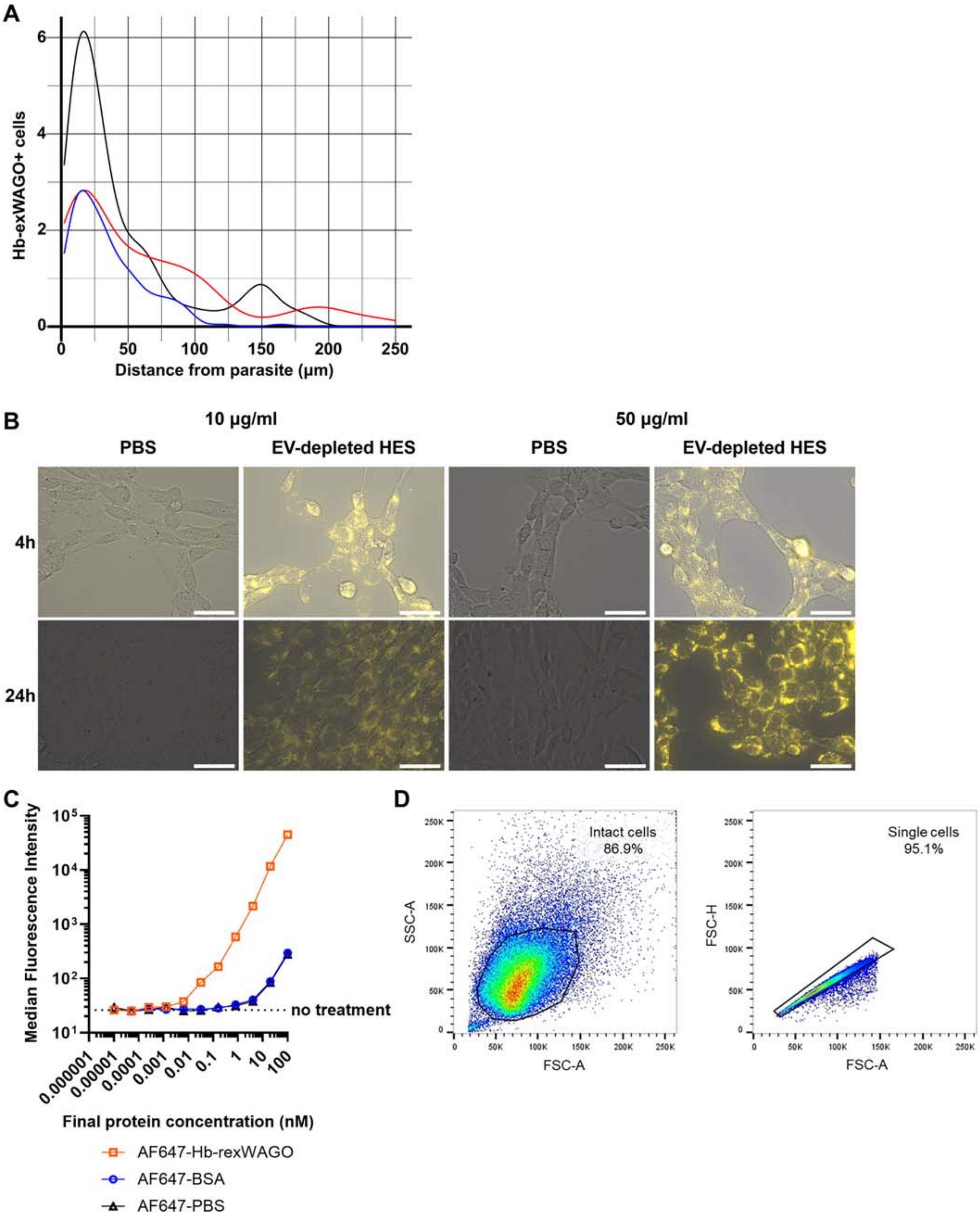
A
Hb-exWAGO+ cells
0
2
4
6
0 50 100 150 200 250
Distance from parasite (µm)
B
10 µg/ml
50 µg/ml
PBS
EV-depleted HES
PBS
EV-depleted HES
4h
24h
C
Median Fluorescence Intensity
10^5
10^4
10^3
10^2
10^1
no treatment
0.000001 0.00001 0.0001 0.001 0.01 0.1 1 10 100
Final protein concentration (nM)
AF647-Hb-rexWAGO
AF647-BSA
AF647-PBS
D
Intact cells
86.9%
SSC-A
FSC-A
Single cells
95.1%
FSC-H
FSC-A
0 50K 100K 150K 200K 250K

Figure EV3. Detection of exWAGO in vivo and fluorescently-labelled proteins internalised by MODE-K cells.

(**A**) Density plot of the distance calculated between Hb-exWAGO positive cells and the parasite (red and black lines are technical replicates; blue line is a biological replicate). (**B**) Brightfield and fluorescence composite microscopy images from MODE-K cells incubated with 10 or 50 μg/ml Cy-5-labelled EV-depleted HES or Cy-5-labelled PBS for 4 or 24 h. Scale bars = 50 μm. Data are representative of 2 biological replicates. (**C**) Dose response of AF647-labelled recombinant Hb-exWAGO (Hb-rexWAGO), BSA or PBS concentrations incubated with MODE-K cells for 4 h measured by flow cytometry ($n = 1$). (**D**) Gating strategy applied for selecting intact cells (left panel) followed by selection of single cells (right panel) applied for flow cytometry analysis.

© The Author(s)

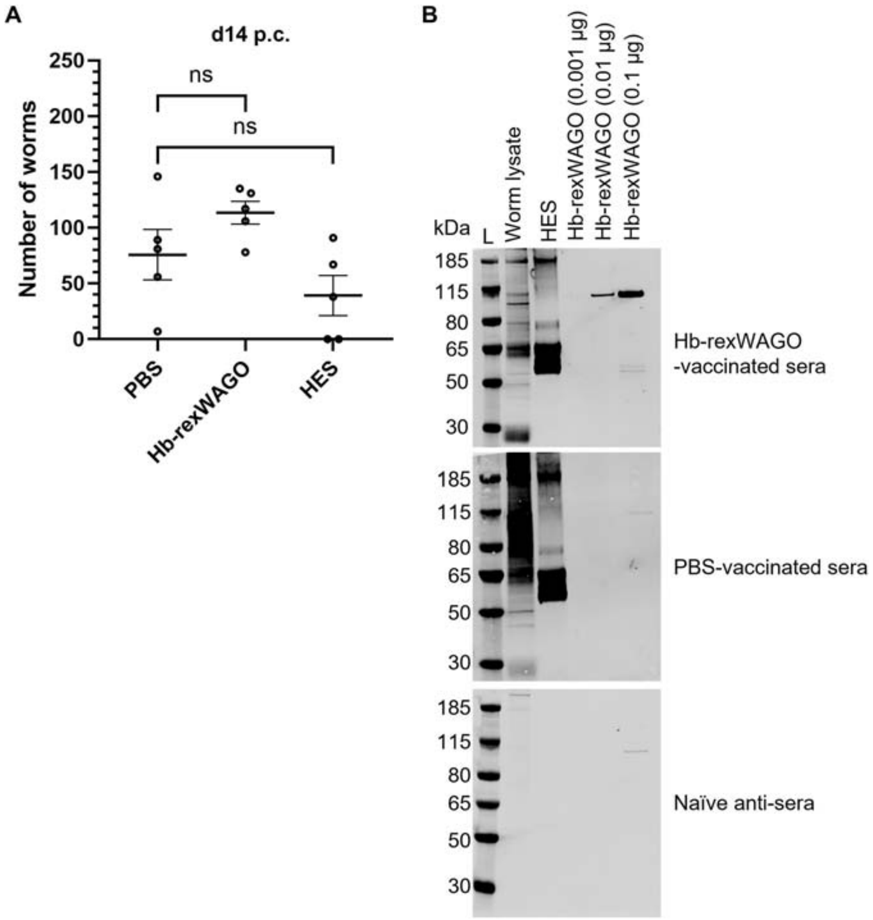

Figure EV4. Parasite and serological responses to vaccine.

(**A**) The number of adult worms 14 days post-challenge (p.c.) recovered in the small intestine following vaccination of mice with recombinant Hb-exWAGO (Hb-rexWAGO), HES, or PBS and challenge with 200 L3 stage larvae ($n = 5$ mice per vaccination group, from one experiment). (**B**) Western blot analysis of IgG responses using pooled sera from Hb-exWAGO- or PBS-vaccinated mice 28 days post-challenge (pool is from $n = 5$ mice in one experiment), or unvaccinated and uninfected mouse (referred to as naïve anti-sera, $n = 1$ mouse). Pooled sera were used as the primary antibody (1:1000 in 5% BSA/TBST) and blots were probed with goat anti-mouse IgG AF680 antibody (1:10,000 in 5% BSA/TBST). Adult worm lysate = 1 µg; HES = 1 µg; recombinant Hb-exWAGO = 0.001–0.1 µg, L = ladder. Data information: In (**A**), individual data points are shown with the mean ± S.E.M. Data were analysed using an unpaired Kruskal–Wallis test (PBS vs Hb-rexWAGO: $P = 0.3814154$; PBS vs HES: $P = 0.2045549$).

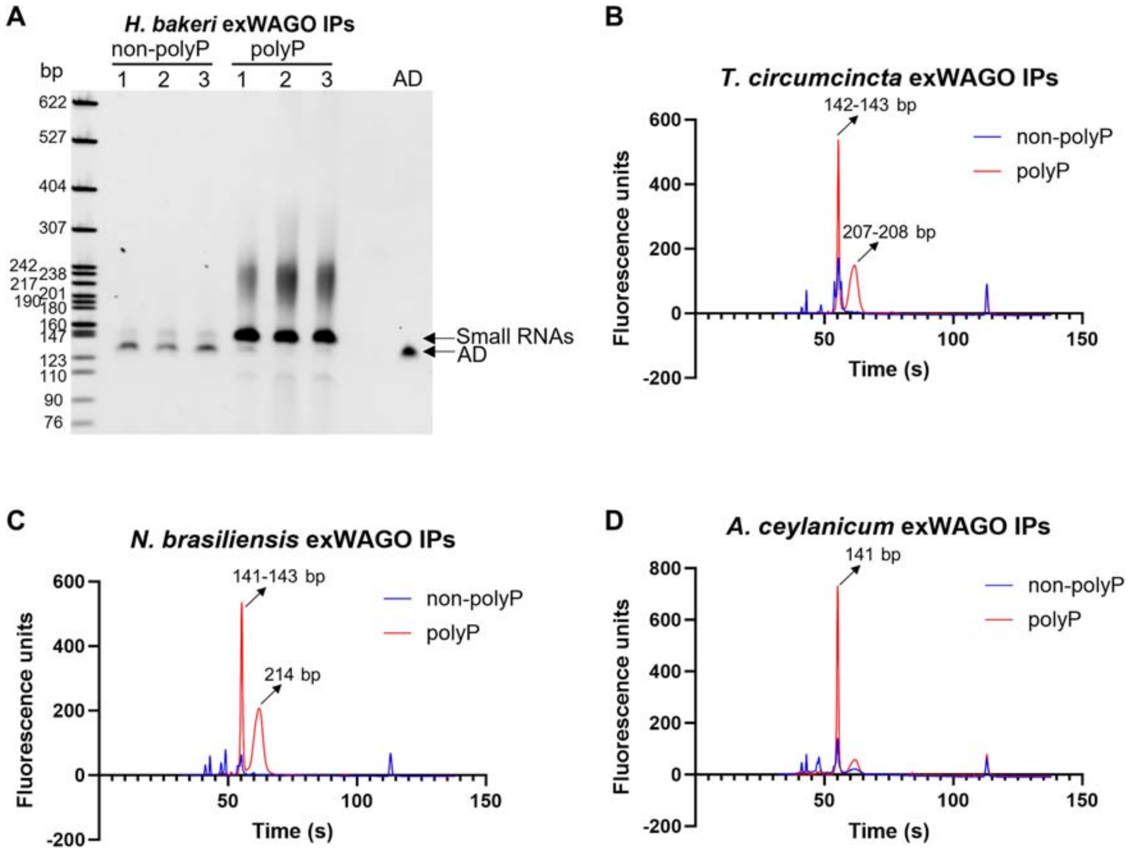

Figure EV5. Analysis of small RNA library profiles treated with or without 5′ RNA polyphosphatase indicates exWAGO guides are 5′ triphosphorylated.

(**A**) Size profile of the small RNA libraries generated following Hb-exWAGO immunoprecipitation from *H. bakeri* adult worms prior to size selection based on TBE 10% gel. 1–3 denote biological replicates. AD = adapter dimers; non-polyP = non-polyphosphatase treated; polyP = polyphosphatase treated. (**B–D**) Representative Bioanalyser High Sensitivity DNA analysis of the small RNA libraries following exWAGO immunoprecipitation from adult (**B**) *T. circumcincta* ($n = 1$ for non-polyP and $n = 2$ biological replicates for polyP), (**C**) *N. brasiliensis* ($n = 2$ biological replicates) and (**D**) *A. ceylanicum* ($n = 1$) worms. The data show the mean fluorescence units where replicates were available. The products enriched during polyphosphatase treatment are indicated. Non-polyP = non-polyphosphatase treated; polyP = polyphosphatase treated.

 © The Author(s)
