# Supplementary material for: An Argonaute protein traffics from nematode to mouse and is a vaccine against parasitic nematodes
Source: EMBO Rep. 2025 Dec 9;27(2):311–40. doi: 10.1038/s44319-025-00620-4 (PMC12852730; doi:10.1038/s44319-025-00620-4)
Supplement: Supplementary file 1 — Peer Review File [file 44319_2025_620_MOESM1_ESM.pdf]

EMBO reports

An Argonaute protein traffics from nematode to mouse and is a vaccine against parasitic nematodes

Kyriaki Neophytou, Isaac Martinez-Ugalde, Thomas Fenton, Elaine Robertson, Lewis Strachan, Vignesh Jayaraman, Yvonne Harcus, Chanel Naar, David Wright, Daniel Price, Ruby White, Michael Evans, Jose Bermudez-Barrientos, Hanchen Li, Rick Maizels, Raffi Aroian, Alasdair Nisbet, Cei Abreu-Goodger, and Amy Buck

Corresponding author(s): Amy Buck (a.buck@ed.ac.uk)

Review Timeline:

Submission Date:	3rd Apr 25
Editorial Decision:	29th Apr 25
Revision Received:	31st Aug 25
Editorial Decision:	30th Sep 25
Revision Received:	18th Oct 25
Accepted:	22nd Oct 25

Editor: Achim Breiling

Transaction Report: No Peer Review Process File is available with this article, as the authors have chosen not to make the review process public.
